# Health behavior interventions among people with lower socio-economic position: a scoping review of behavior change techniques and effectiveness

**DOI:** 10.1080/21642850.2024.2365931

**Published:** 2024-06-18

**Authors:** Loes van den Bekerom, Laurens C. van Gestel, Jan W. Schoones, Jet Bussemaker, Marieke A. Adriaanse

**Affiliations:** a Health Campus The Hague/Department of Public Health and Primary Care, Leiden University Medical Center, The Hague/Leiden, the Netherlands; b Health, Medical and Neuropsychology Unit, Leiden University, Leiden, the Netherlands; c Directorate of Research Policy, Leiden University Medical Center, Leiden, the Netherlands; d The Institute of Public Administration, Leiden University, Leiden, the Netherlands

**Keywords:** health behavior interventions; behavior change techniques (BCTs); socio-economic position (SEP); healthy eating; scoping review

## Abstract

**Background:**

Behavior change interventions can unintendedly widen existing socio-economic health inequalities. Understanding why interventions are (in)effective among people with lower socio-economic position (SEP) is essential. Therefore, this scoping review aims to describe what is reported about the behavior change techniques (BCTs) applied within interventions and their effectiveness in encouraging physical activity and healthy eating, and reducing smoking and alcohol consumption according to SEP.

**Methods:**

A systematic search was conducted in 12 electronic databases, and 151 studies meeting the eligibility criteria were included and coded for health behavioral outcomes, SEP-operationalization, BCTs (type and number) and effectiveness.

**Results:**

Findings suggest that approaches for measuring, defining and substantiating lower SEP vary. Current studies of behavior change interventions for people of different SEP do not systematically identify BCTs, making systematic evaluation of BCT effectiveness impossible. The effectiveness of interventions is mainly evaluated by overall intervention outcomes and SEP-moderation effects are mostly not assessed.

**Conclusion:**

Using different SEP-operationalizations and not specifying BCTs hampers systematic evidence accumulation regarding effective (combinations of) BCTs for the low SEP population. To learn which BCTs effectively improve health behaviors among people with lower SEP, future intervention developers should justify how SEP is operationalized and must systematically describe and examine BCTs.

## Introduction

Being healthy is crucial to function optimally, since it is important for long-term well-being and prevention of disease burden and mortality. This, however, is not a given for everyone. Health outcomes are closely associated with individuals’ socio-economic position (SEP), as indicated by factors such as educational attainment, income and occupational status. More specifically, lower SEP is related to a higher risk for chronic diseases, disability and death (e.g. Adler et al., 1993; Bartley, 2004; Drewnowski et al., 2014; Marmot, 2004). In the Netherlands, for example, individuals with lower SEP live on average six years less, and live 15 years longer in poor health than people with higher SEP (Pharos, 2022). As health outcomes are unevenly distributed across different social groups within countries, one of the international Sustainable Development Goals includes ensuring healthy lives and promoting the well-being for all at all ages (United Nations, n.d.). Hence, tackling SEP-differences in health is of special importance.

Several factors contribute to these differences in health outcomes in different social groups. Besides socio-economic factors (e.g. dealing with low literacy and poverty), a substantial proportion of health disparities can be attributed to SEP-differences in health behaviors (for reviews, see Kraft & Kraft, 2021; Petrovic et al., 2018). Overall, people with lower SEP are more prone to having unhealthy lifestyles than people with higher SEP (Stringhini et al., 2010). Specifically, they are less likely to meet existing guidelines of healthy diet and physical activity and rates of smoking and binge drinking are higher among them (for reviews, see Pampel et al., 2010; Probst et al., 2020). These unhealthy behaviors (i.e. physical inactivity, unhealthy diet, smoking and heavy drinking) contribute to global disease burden and premature death (e.g. Bauer et al., 2014; Kontis et al., 2014; Ministry of Health, Welfare and Sport [MHWS], 2019) and explain the majority of the link between SEP and mortality rates (Gruer et al., 2009; Hart et al., 2011; Nandi et al., 2014; Stringhini et al., 2010; Whitley et al., 2014).

Hence, in order to tackle health inequalities, it is essential to target health behaviors dependent on individual’s socio-economic context (Marteau et al., 2021). Changing them may increase life expectancy, and more importantly, *healthy* life expectancy. That is why European governments, for example, are aiming to improve the health of their citizens by tackling their excess weight, smoking habits and problematic drinking (e.g. The Dutch National Prevention Agreement; MHWS, 2019; The United Kingdom’s consultation document; Department of Health & Social Care, 2019). Despite increasing efforts to develop effective preventive public health interventions (Gitlin & Czaja, 2015; Mohr et al., 2013), however, health inequity has not been reduced during the last decades (Eikemo et al., 2017; Marmot, 2020). In fact, in several cases interventions have even been found to unintentionally widen this gap by benefiting advantaged groups more than disadvantaged groups (McGill et al., 2015; White et al., 2009), due to differences in the efficacy of the intervention, but also to reduced accessibility to, adoption of and adherence to interventions (Bukman et al., 2014; Estabrooks et al., 2003; Van Oort et al., 2005; Veinot et al., 2018). It is thus evident that we need to improve our understanding of how health behaviors may be effectively promoted in low SEP populations. The present scoping review aims to address this issue by describing what is reported about the effectiveness of current behavior change interventions in lower (versus higher) SEP groups aiming to encourage physical activity and healthy eating and reduce tobacco use and alcohol consumption. Aggregating knowledge on behavioral interventions requires an analysis on the level of the active ingredients that are applied and tested within these interventions (i.e. behavior change techniques; BCTs), including an examination of the question of whether this information is specified and how SEP is operationalized.

### Operationalization of SEP

Examining what behavior change interventions report about their techniques and effectiveness among people with lower SEP may depend on how SEP is operationalized within the current literature. SEP is a multi-dimensional construct that can be perceived as a set of socio-economic conditions, and represents an individual’s possession of normatively valued social and economic resources (Antonoplis, 2023). It is generally considered to ‘encompass not only income but also educational attainment, occupational prestige, and subjective perceptions of social status and social class’ (American Psychological Association (APA), 2022). Within behavioral research, various terms are used to describe SEP, such as ‘socio-economic status’, ‘social class’, ‘social inequality’, ‘social inequity’ and ‘socio-economic disparity’ (Berzofsky et al., 2014; Braveman et al., 2005). Within this paper, SEP will be used broadly, encompassing individual’s absolute level of resources (i.e. objective indicators, such as education, income, occupation or place of residence; APA, 2022; Berzofsky et al., 2014; Braveman et al., 2005; Cirino et al., 2002; Shavers, 2007) as well as subjective perceptions of an individual’s standing in the socio-economic hierarchy compared to others (e.g. a rank- or ladder-based assessment of subjective status; Adler et al., 2000; Kluegel et al., 1977; Kraft & Kraft, 2021). While SEP can reflect diverse theoretical matters and its components are distinct, non-interchangeable features (Antonoplis, 2023; Duncan & Magnuson, 2012; Pampel et al., 2010), it is often conveniently used as an umbrella term for various indicators of someone’s position within society.

There is no standard method for measuring SEP, as health researchers can use many different approaches depending on the conceptual model being employed, the study design and the data available, seemingly without justification (Antonoplis, 2023; Braveman et al., 2005). Such variation in SEP has influenced the understanding of the associations between SEP characteristics and health outcomes (Braveman et al., 2005; Shavers, 2007). Specifically, using different indicators of SEP can result in contradictory results about how SEP relates to an outcome (Antonoplis, 2023), which could also lead to different conclusions regarding the effectiveness of behavior change interventions (and their techniques) among people from divergent SEP groups. The current review focuses on frequently studied core-components of SEP that are related to health inequalities, such as income, poverty, occupation, education, place of residence and subjectively experienced social status. The current review therefore does not include other characteristics along which one can stratify health outcomes, such as age, race, sex, gender (identity) or religion (O'Neill et al., 2014). Within this particular focus, the first aim of the present study is to map how SEP is operationalized within behavioral intervention research.

### Health behavior change: techniques and effectiveness

Health interventions make use of techniques that guide behavior change. These BCTs (e.g. goal setting, using prompts) are the observable, replicable and irreducible components of an intervention that foster behavior change, by reinforcing factors that facilitate behavior change or by addressing barriers to behavior change. Such techniques are the active ingredients of interventions that actively alter or redirect causal processes that regulate behavior (see BCT taxonomy v1; Michie et al., 2013). Behavior change interventions are typically complex, as they may involve many interacting BCTs (Carey et al., 2019; Craig & Petticrew, 2013; Michie et al., 2013). They can be implemented in different formats and can be used alone or in combination (Michie et al., 2013). To illustrate, interventions can incorporate up to 20 BCTs (e.g. for reviews see Michie et al., 2009; Bull et al., 2018) and could, for example, involve combinations of providing information, setting specific goals and self-monitoring.

Behavior is suggested to be part of an interacting system involving different underlying determinants such as one’s capability (e.g. decision processes or skills), opportunity (e.g. the physical or social environment) and motivation (e.g. goals or emotions), and interventions (and their different BCTs) can target one or more of these behavioral determinants to stimulate a specific behavioral target (Michie et al., 2011). Scholars have been putting effort into getting a thorough understanding of the processes underlying BCT effectiveness, by identifying the links between BCTs and the Mechanisms of Action (MoAs) they target, i.e. the theoretical constructs that represent the processes through which BCTs are proposed to have their behavioral effects, such as knowledge, beliefs about capabilities, and social influence (Carey et al., 2019; Connell et al., 2019; Human Behaviour Change, n.d; Johnston et al., 2018; Michie et al., 2018). However, this line of research – where BCTs are directly associated with MoAs – assumes that the links between these components are generalizable across different populations, while behavior change may depend on SEP (e.g. Adams & White, 2007). Specifically, behavioral determinants and the way they interact with behavior may depend on SEP (e.g. Adams & White, 2007; Pampel et al., 2010; Schüz et al., 2017), and due to such differences, the effectiveness of BCTs themselves may be different for lower and higher SEP groups. To elaborate, previous research demonstrates that the effectiveness of BCTs can be moderated by age (e.g. French et al., 2020), motivation (e.g. Prestwich & Kellar, 2014), personality traits (e.g. Prestwich & Kellar, 2014), and executive functioning (Hall et al., 2014; Pfeffer & Strobach, 2020). Among people with lower SEP, lower levels of executive functioning can be observed (Hackman & Farah, 2009; Hackman et al., 2015), as well as different motives for food selection (Konttinen et al., 2021). Importantly, such relationships can likely be explained by differences in access to both tangible and intangible resources. For example, executive functioning can be impaired by experiences of stress (Liston et al., 2009) and financial scarcity (O'Neill et al., 2021). Since people with lower and higher SEP may experience differences in access to such resources, it can be assumed that BCTs may have divergent effects among them.

Thus, before assessing whether BCTs have their effects through different MoAs according to SEP, a conditional step should be to examine whether BCTs are effective across different SEP groups. However, relatively little attention has been paid within behavior change research on the effectiveness of specific BCTs within intervention programs for different SEP groups. This mainly provides evidence about ‘what works’ across different SEP groups, while for the further development of behavior change interventions evidence about how interventions work for specific SEP groups (i.e. ‘when’ and ‘for whom’) is crucial (Michie et al., 2009; Rothman & Sheeran, 2020). This highlights the need to understand whether the current behavior change literature provides evidence about the distinct BCTs that are applied within existing interventions and their effectiveness in stimulating health outcomes *according to* SEP.

### Present scoping review

The primary aim of the current scoping review is to map the current behavior change intervention literature, and specifically to describe what is known about the application of BCTs within behavior change interventions and their effectiveness in encouraging physical activity and healthy eating, and reducing smoking and alcohol consumption among people with lower (versus higher) SEP. As we attempt to scope a broad body of literature on the available evidence to examine how research is conducted on this topic, a scoping review is the most valid approach (Munn et al., 2018). Obviously, this is not the first review describing behavioral interventions and techniques according to SEP, but previous reviews targeted specific low SEP samples (as indicated by demographic characteristics (e.g. children or adolescents specifically) and SEP operationalizations (e.g. low income only)), specific modes of delivery (e.g. e- and m-health or school-based interventions) and/or some distinct health behaviors (e.g. energy balance, healthy eating, soft drink intake, physical activity, alcohol consumption and/or smoking; Anselma et al., 2020; Bull et al., 2014; Bull et al., 2018; Harakeh et al., 2024; Michie et al., 2009; Moore et al., 2015; Ronteltap et al., 2022; Shagiwal et al., 2020; Western et al., 2021). Thus, despite previous attempts to describe BCTs within behavior change interventions according to SEP, current literature is fragmented and a clear and comprehensive overview is lacking.

The current scoping review addresses this knowledge gap by describing what scholars report about the specific BCTs of behavior change interventions and their effectiveness across multiple health outcomes *and* according to SEP, as characterized by multiple objective and subjective SEP-indicators. This review focuses on interventions that aim to alter physical activity, healthy eating, smoking and/or alcohol consumption, as these four health behaviors are the most widely targeted and most commonly communicated in policy and health campaigns. In addition, the current review focuses on the core objective indicators of SEP, such as income, poverty, occupation, education and place of residence, as well as subjectively experienced social status. Within this focus, we will outline (1) how SEP is operationalized, (2) whether BCTs are specified (and if so, how many and what type of BCTs are used), (3) what is reported about the effectiveness of behavior change interventions and techniques, (4) whether SEP-moderations are examined (comparing effects between lower and higher SEP groups), and (5) whether explanations are provided for SEP-differences in health outcomes (in terms of efficacy, accessibility, adoption and adherence). In doing so, we will describe what is known but also identify knowledge gaps within the current literature to guide future research into the potential of BCTs that also benefit people from more disadvantaged groups. This is crucial to improve health at the population level.

## Materials and methods

The present scoping review follows the guidance document for the conduct of scoping reviews published by the Joanna Briggs Institute (JBI; Peters et al., 2017). The reporting follows the guideline of the Preferred Reporting Items for Systematic reviews and Meta-Analyses extension for Scoping Reviews (PRISMA-ScR; Tricco et al., 2018). Ethics/Institutional Review Board approval was not required to review published/publicly reported literature.

### Protocol and registration

In line with the JBI and PRISMA-ScR guidelines, our protocol for the scoping review was registered prospectively and made public a priori on the Open Science Framework (https://osf.io/25eyg/).

### Eligibility criteria

To explore different types of research within this review, quantitative, qualitative and mixed-method empirical research published in English or Dutch within articles of peer-reviewed journals, (chapters of) books, doctoral dissertations and (bachelor and master) theses were eligible for inclusion. Meta-analyses and reviews were excluded.^1^ In line with previous reviews that included literature over a period of about 20 years (e.g. Anselma et al., 2020; Ronteltap et al., 2022), we included sources that were published from January 2000 onwards. This time span allowed us to address the aims of our review while focusing on interventions that are generalizable to the current understanding and operationalizations of interventions among lower SEP groups (which have been increasingly developed in recent times). Sources were eligible when the following inclusion criteria were met: (1) the intervention aimed to promote behavioral outcomes relevant to at least one of the four health behaviors targeted (i.e. physical activity, healthy eating, reduction in tobacco use and reduction in alcohol consumption), regardless of whether they were the primary or secondary outcomes, (2) the intervention was in any case targeted at participants from lower SEP groups, possibly supplemented with participants from higher SEP groups as a comparison group, (3) the intervention was targeted at a general adult population (18+ years). Regarding the second criterion, no predetermined definitions or strict cut-off points were used and papers were included if the authors described that the sample consisted of people with low SEP, regardless of how they defined or substantiated low SEP (this was coded and analyzed in this review).

To limit heterogeneity and thus increase feasibility, research papers were excluded from this review if interventions were administered completely or mainly via electronic tools (e-health), mobile tools (m-health) and schools. E- and m-health interventions encompass a specific niche within behavioral intervention research that has already received considerable attention in existing reviews on behavior change in low SEP populations (e.g. Ronteltap et al., 2022; Western et al., 2021), and studies have concluded that the effectiveness of these interventions is frequently negatively influenced due to problems with implementation (e.g. reach, adoption; Al-Dhahir et al., 2022; Pederson et al., 2019; Veinot et al., 2018). We also focused on adult populations and therefore excluded school-based interventions. During abstract screening, papers that described any form of e- or m-health were excluded, as we deemed it to be a substantial part of the intervention at this stage. During full-text screening, papers that described interventions with follow-up or booster components delivered through means of e- or m-health (e.g. follow-up telephone calls) that involved a repetition of components provided during the main (face-to-face) intervention, or only a check-up, were included in this phase. Papers that described interventions that used e- or m health tools only for measurement purposes (e.g. measuring step count with a pedometer) were included, while papers that used such tools as active intervention components aimed to promote behavior change (e.g. encouraging pedometer use for self-monitoring) were excluded. As scoping reviews are exploratory assessments of the available evidence, there were no restrictions regarding country of origin, study design or intervention characteristics (e.g. type, duration).

### Selection process

#### Strategy

The search strategy was developed in collaboration with an experienced librarian (JS). Initially, the principal researchers provided key articles from previous literature and corresponding keywords. During the literature search, an iterative process was used to build, refine and optimize the strategy (e.g. identifying additional keywords and sources, narrowing or widening the search strategy). First, an initial limited search of at least two appropriate databases was performed on pre-defined keywords to identify additional relevant keywords and index terms. After that, a search using all identified keywords and index terms was undertaken across all relevant databases.^2^

#### Information sources and search strategy

The final systematic search (step 2) was performed on 07-10-2022 in the following bibliographic databases: PubMed, Medline (OVID), Embase (OVID), Emcare (OVID), Web of Science (Core Collection), Cochrane Library (Wiley), PsycINFO (EbscoHOST), PsycArticles (EbscoHOST), Psychology and Behavioral Sciences Collection (EbscoHOST), Academic Search Premier (EbscoHOST), ProQuest Dissertations and Theses, and Google Scholar. An example of the search strategy for PubMed is presented in Table 1. The final search strategy for all databases can be found in Supplementary file 1. The final search results were exported to EndNote and duplicates were removed (automatically and manually).

**Table 1.** Example of the search strategy (PubMed).

Building block	Search phrase
Low SEP	(("Economic Status"[mesh] OR "Educational Status"[mesh] OR "Employment"[mesh] OR "Unemployment"[mesh] OR "Income"[mesh] OR "Salaries and Fringe Benefits"[mesh] OR "Occupations"[mesh] OR "Poverty"[mesh] OR "Poverty Areas"[mesh] OR "Social Class"[mesh] OR "Social Conditions"[mesh] OR "Economic Status"[tiab] OR "Educational Status"[tiab] OR "Employment"[tiab] OR "Income"[tiab] OR "Occupation"[tiab] OR "Occupations"[tiab] OR "Poverty"[tiab] OR "Poverty Area"[tiab] OR "Poverty Areas"[tiab] OR "Salaries"[tiab] OR "Salary"[tiab] OR "Social Class"[tiab] OR "Social Classes"[tiab] OR "Social Condition"[tiab] OR "Social Conditions"[tiab] OR "Unemployment"[tiab] OR "socioeconomic position"[tiab] OR "socioeconomic positions"[tiab] OR "socioeconomic posit*"[tiab] OR "socio economic position"[tiab] OR "socio economic positions"[tiab] OR "socio economic posit*"[tiab] OR "social economic position"[tiab] OR "social economic posit*"[tiab] OR "socioeconomic status"[tiab] OR "socio economic status"[tiab] OR "social economic status"[tiab] OR "deprived urban area"[tiab] OR "deprived suburban area"[tiab] OR "deprived area"[tiab] OR "blue collar"[tiab] OR "blue-collar"[tiab] OR "deprive*"[tiab] OR "deprived"[tiab] OR "disadvantaged"[tiab] OR "education level*"[tiab] OR "educational level"[tiab] OR "employment*"[tiab] OR "income*"[tiab] OR "job"[tiab] OR "jobs"[tiab] OR "low education"[tiab] OR "low income"[tiab] OR "low-educat*"[tiab] OR "low-income"[tiab] OR "social class*"[tiab] OR "social disparity"[tiab] OR "social disparities"[tiab] OR "social disparit*"[tiab] OR "social inequalit*"[tiab] OR "social inequalities"[tiab] OR "social inequality"[tiab] OR "social inequit*"[tiab] OR "social inequities"[tiab] OR "social inequity"[tiab] OR "Health Inequities"[Mesh] OR "Health inequity"[tiab] OR "Health inequality"[tiab] OR "Health inequities"[tiab] OR "Health inequalities"[tiab] OR "Health inequit*"[tiab] OR "Health inequalit*"[tiab] OR "Healthcare Disparities"[Mesh] OR "Healthcare Disparities"[tiab] OR "Healthcare Disparity"[tiab] OR "Health care Disparities"[tiab] OR "Health care Disparity"[tiab] OR "social position"[tiab] OR "social standing"[tiab] OR "social status"[tiab] OR "social strata"[tiab] OR "socioeconomic"[tiab] OR "socio-economic"[tiab] OR "socio-economic"[tiab] OR "socioeconomic factor"[tiab] OR "socioeconomic factors"[tiab] OR "Socio-economic factors"[tiab] OR "Socio-economic factor"[tiab] OR "socio-economic status"[tiab] OR "socioeconomically"[tiab] OR "socio-economically"[tiab] OR "underprivileged"[tiab] OR "unemployed"[tiab] OR "working class"[tiab] OR "working-class "[tiab] OR "years of education"[tiab] OR "years of schooling"[tiab] OR "job status"[tiab] OR "occupational status"[tiab] OR "occupation status"[tiab])
Health behaviors	AND ("Exercise"[Mesh] OR "Physical activity"[tiab] OR "Physical activities"[tiab] OR "Exercise"[tiab] OR "Exercises"[tiab] OR "Exercising"[tiab] OR "Exercis*"[tiab] OR "Strength training"[tiab] OR "Aerobic"[tiab] OR "Aerobics"[tiab] OR "Resistance training"[tiab] OR "Walking"[tiab] OR "Endurance Training"[tiab] OR "Exergaming"[tiab] OR "Gymnastics"[tiab] OR "Interval Training"[tiab] OR "Jogging"[tiab] OR "Nordic Walking"[tiab] OR "Physical Conditioning"[tiab] OR "Running"[tiab] OR "Stair Climbing"[tiab] OR "Swimming"[tiab] OR "Sitting"[tiab] OR "Sedentary Behavior"[Mesh] OR "Sedentary behaviour"[tiab] OR "Sedentary behavior"[tiab] OR "Activity"[tiab] OR "activities"[tiab] OR "Inactivity"[tiab] OR "inactivities"[tiab] OR "Inactivit*"[tiab] OR "Diet"[mesh] OR "diet"[tiab] OR "diets"[tiab] OR "diet*"[tiab] OR "nutrition"[tiab] OR "nutritional"[tiab] OR "nutrition*"[tiab] OR "Eating"[mesh] OR "eat"[tiab] OR "eating"[tiab] OR "consumption"[tiab] OR "food intake"[tiab] OR "food pattern"[tiab] OR "food habit"[tiab] OR "food patterns"[tiab] OR "food habits"[tiab] OR "food intake"[tiab] OR "Food"[mesh] OR "food"[tiab] OR "foods"[tiab] OR "food-related"[tiab] OR "vegetable"[tiab] OR "vegetables"[tiab] OR "fruit"[tiab] OR "fruits"[tiab] OR "wholegrain"[tiab] OR "wholegrains"[tiab] OR "legume*"[tiab] OR "nut"[tiab] OR "nuts"[tiab] OR "dairy"[tiab] OR "fish"[tiab] OR "tea"[tiab] OR "fat"[tiab] OR "fats"[tiab] OR "oil"[tiab] OR "oils"[tiab] OR "coffee"[tiab] OR "red meat"[tiab] OR "processed meat"[tiab] OR "Food and Beverages"[Mesh] OR "Beverages"[Mesh] OR "sweetened beverage*"[tiab] OR "juice*"[tiab] OR "Drinking Behavior"[mesh] OR "Drinking Behavior"[tiab] OR "Drinking Behaviour"[tiab] OR "Alcohol Drinking"[mesh] OR "Ethanol"[Mesh] OR "Ethanol"[tiab] OR "alcohol"[tiab] OR "Alcohol consumption"[tiab] OR "Alcohol drinking"[tiab] OR "alcohol use"[tiab] OR "Energy Intake"[Mesh] OR "Weight Gain"[Mesh] OR "Weight gain"[tiab] OR "gain weight"[tiab] OR "gaining weight"[tiab] OR "poor diet"[tiab] OR "poor diets"[tiab] OR "poor dietary"[tiab] OR "healthy eating"[tiab] OR "Smoking"[mesh] OR "Smoking"[tiab] OR "Smoking Cessation"[mesh] OR "Smoking Reduction"[mesh] OR "Tobacco Use Cessation"[mesh] OR "Smoking cessation"[tiab] OR "Tobacco"[mesh] OR "Tobacco Products"[mesh] OR "Tobacco use"[mesh] OR "Tobacco use"[tiab])
Behavior change (intervention)	AND ("behavior change"[tiab] OR "behavior changes"[tiab] OR "behavior chang*"[tiab] OR "behavioral change"[tiab] OR "behavioral changes"[tiab] OR "behavioral chang*"[tiab] OR "behaviour change"[tiab] OR "behaviour changes"[tiab] OR "behaviour chang*"[tiab] OR "behavioural change"[tiab] OR "behavioural changes"[tiab] OR "behavioural chang*"[tiab] OR "behavior change interventions"[tiab] OR "behavior change intervention"[tiab] OR "behaviour change interventions"[tiab] OR "behaviour change intervention"[tiab] OR "behavioral change interventions"[tiab] OR "behavioral change intervention"[tiab] OR "behavioural change interventions"[tiab] OR "behavioural change intervention"[tiab] OR "behavior change techniques"[tiab] OR "behavior change technique"[tiab] OR "behaviour change techniques"[tiab] OR "behaviour change technique"[tiab] OR "behavioral change techniques"[tiab] OR "behavioral change technique"[tiab] OR "behavioural change techniques"[tiab] OR "behavioural change technique"[tiab] OR "Behavior Change"[tiab] OR "Behavior Changes"[tiab] OR "Behavior experiment"[tiab] OR "Behavior experiments"[tiab] OR "Behavior Intervention"[tiab] OR "Behavior Interventions"[tiab] OR "Behavior Modification"[tiab] OR "Behavior Modifications"[tiab] OR "Behavior Program"[tiab] OR "Behavior Programme"[tiab] OR "Behavior Programmes"[tiab] OR "Behavior Programs"[tiab] OR "Behavior Promotion"[tiab] OR "Behavior Promotions"[tiab] OR "Behavior Trial"[tiab] OR "Behavior Trials"[tiab] OR "Behavioral Change"[tiab] OR "Behavioral Changes"[tiab] OR "Behavioral experiment"[tiab] OR "Behavioral experiments"[tiab] OR "Behavioral Intervention"[tiab] OR "Behavioral Interventions"[tiab] OR "Behavioral Modification"[tiab] OR "Behavioral Modifications"[tiab] OR "Behavioral Program"[tiab] OR "Behavioral Programme"[tiab] OR "Behavioral Programmes"[tiab] OR "Behavioral Programs"[tiab] OR "Behavioral Promotion"[tiab] OR "Behavioral Promotions"[tiab] OR "Behavioral Trial"[tiab] OR "Behavioral Trials"[tiab] OR "Behaviors Change"[tiab] OR "Behaviors Changes"[tiab] OR "Behaviors experiment"[tiab] OR "Behaviors experiments"[tiab] OR "Behaviors Intervention"[tiab] OR "Behaviors Interventions"[tiab] OR "Behaviors Modification"[tiab] OR "Behaviors Modifications"[tiab] OR "Behaviors Program"[tiab] OR "Behaviors Programme"[tiab] OR "Behaviors Programmes"[tiab] OR "Behaviors Programs"[tiab] OR "Behaviors Promotion"[tiab] OR "Behaviors Promotions"[tiab] OR "Behaviors Trial"[tiab] OR "Behaviors Trials"[tiab] OR "Behaviour Change"[tiab] OR "Behaviour Changes"[tiab] OR "Behaviour experiment"[tiab] OR "Behaviour experiments"[tiab] OR "Behaviour Intervention"[tiab] OR "Behaviour Interventions"[tiab] OR "Behaviour Modification"[tiab] OR "Behaviour Modifications"[tiab] OR "Behaviour Program"[tiab] OR "Behaviour Programme"[tiab] OR "Behaviour Programmes"[tiab] OR "Behaviour Programs"[tiab] OR "Behaviour Promotion"[tiab] OR "Behaviour Promotions"[tiab] OR "Behaviour Trial"[tiab] OR "Behaviour Trials"[tiab] OR "Behavioural Change"[tiab] OR "Behavioural Changes"[tiab] OR "Behavioural experiment"[tiab] OR "Behavioural experiments"[tiab] OR "Behavioural Intervention"[tiab] OR "Behavioural Interventions"[tiab] OR "Behavioural Modification"[tiab] OR "Behavioural Modifications"[tiab] OR "Behavioural Program"[tiab] OR "Behavioural Programme"[tiab] OR "Behavioural Programmes"[tiab] OR "Behavioural Programs"[tiab] OR "Behavioural Promotion"[tiab] OR "Behavioural Promotions"[tiab] OR "Behavioural Trial"[tiab] OR "Behavioural Trials"[tiab] OR "Behaviours Change"[tiab] OR "Behaviours Changes"[tiab] OR "Behaviours experiment"[tiab] OR "Behaviours experiments"[tiab] OR "Behaviours Intervention"[tiab] OR "Behaviours Interventions"[tiab] OR "Behaviours Modification"[tiab] OR "Behaviours Modifications"[tiab] OR "Behaviours Program"[tiab] OR "Behaviours Programme"[tiab] OR "Behaviours Programmes"[tiab] OR "Behaviours Programs"[tiab] OR "Behaviours Promotion"[tiab] OR "Behaviours Promotions"[tiab] OR "Behaviours Trial"[tiab] OR "Behaviours Trials"[tiab] OR (("promotion"[ti] OR "promote"[ti] OR "promoting"[ti]) AND ("intervention"[ti] OR "interventions"[ti])))
Exclusion criteria	NOT ("Telemedicine"[majr] OR "e-health"[ti] OR "m-health"[ti] OR "ehealth"[ti] OR "mhealth"[ti] OR "telehealth"[ti] OR "schoolbased"[ti] OR "school based"[ti] OR "School Health Services"[majr] OR "digital intervention"[ti] OR "digital interventions"[ti] OR "digital"[ti] OR "online"[ti] OR "internet"[ti] OR "smartphone"[ti] OR "smartphones"[ti] OR "phone"[ti] OR "phones"[ti]) AND ("2000/01/01"[PDAT] : "3000/12/31"[PDAT]) NOT (("Infant"[mesh] OR "infant"[ti] OR "infants"[ti] OR "Child"[mesh] OR "child"[ti] OR "children"[ti] OR "childhood"[ti] OR "girl"[ti] OR "girls"[ti] OR "girlhood"[ti] OR "boy"[ti] OR "boys"[ti] OR "boyhood"[ti] OR "Adolescent"[mesh] OR "adolescent"[ti] OR "adolescents"[ti] OR "adolescence"[ti]) NOT ("Adult"[mesh] OR "adult"[ti] OR "adults"[ti] OR "adulthood"[ti] OR "elderly"[ti])))

#### Selection of sources of evidence

After the literature search was completed, all identified papers were collated to screen the titles and abstracts within ASReview (v 1.0; Van de Schoot et al., 2021, 2022), a free open-source software program that uses active learning techniques for literature screening. Two reviewers (LvdB and LvG) independently screened papers on title and abstract for assessment against the eligibility criteria. Both reviewers screened the papers until they screened at least 36% of all uploaded papers *and* screened 25 consecutive non-relevant papers (our stop criterion, conservatively derived from previous research demonstrating the efficiency of the software; Ferdinands et al., 2020; Van de Schoot et al., 2021).

The screening resulted in 314 papers that were indicated as eligible for retrieval and full-text screening by both reviewers. Cases where the two reviewers had made a different decision regarding in- or exclusion (*n* = 310) were discussed. Hereby, the titles and abstracts were screened again and a joint conclusion was reached, resulting in an additional 167 eligible papers. Additionally, titles and abstracts that were only screened by one of the reviewers due to different algorithms and were included by that reviewer (*n* = 40), were also screened by the other reviewer and were discussed. This resulted in an additional 29 eligible papers, bringing about a total of 510 eligible papers. Hereafter, the full-text articles were retrieved, read and assessed in detail against the eligibility criteria by one reviewer (LvdB) and uncertainties were checked by or discussed with the second reviewer (LvG). At this stage, papers were excluded if both reviewers agreed that they deviated from the inclusion criteria. Reasons for exclusion of papers at full text are recorded in Supplementary file 2 (sheet ‘Full-text screening’). Any disagreement on study selection between the two reviewers was discussed, and an additional reviewer (MA) was involved if discussion did not lead to conclusive agreements. Full-text articles that were not freely available, were requested from the corresponding authors. We, however, did not receive any response resulting in exclusion of these papers (*n* = 8) due to unavailability.

#### Data charting: process and form

Before data extraction, a predefined data charting form was determined by the research team. For an extensive description of all variables extracted, see Table 2. Data of all included studies were extracted by one independent reviewer (LvdB) regarding article characteristics (e.g. title of publication, author(s), year of publication), study characteristics (e.g. origin, study design, intervention details) and main outcome variables relevant for the objectives (e.g. operationalization SEP, health behaviors, BCTs, effectiveness intervention). Data extraction for BCTs was slightly changed during the data charting process (see Table 2). In addition to coding whether specific BCTs were mentioned in the paper, it was coded whether all BCTs in the intervention were specified in the paper, whether only some BCTs were mentioned (e.g. as an example or to specify the most important BCTs) or whether BCTs were not specified at all. If all or at least some BCTs were specified it was coded whether this was done according to the BCT taxonomy (v1; Michie et al., 2013) or not. We also listed which BCTs were specified for papers that completely described an intervention on technique-level. If this was not done according to the BCT taxonomy in the papers (*n* = 59), LvdB used the available information in the paper to code the techniques according to the BCT taxonomy. Any uncertainties during data charting were checked by or discussed with the second reviewer (LvG). For the final data charting form, see Supplementary file 2 (sheet ‘Data extraction’).

**Table 2.** Description of variables and data extracted.

Category	Type of data	Description (*Data extracted*)
1. Article characteristics	ID	Number of paper
	ID ASReview	ID number from ASReview
	Title	Title of publication
	Author(s)	Names of all authors (*Surname author(s), Initials author(s)*)
	Journal	The journal where the paper is published
	Year	Year of publication
	Type	Type of publication (*Choose: Original research, Commentary, Other: namely*)
	Language	The language of the publication
	Study number	If the article contains multiple studies, number of the study
2. Study characteristics	Origin	The country in which the intervention was carried out or the country from which the sample was collected (for example in online experiments)
	Study method	Type of method used (*Choose: Quantitative, Qualitative or Mixed-methods*)
	Study design	Description of the design of the study (*E.g. pre-post, RCT, field experiment, laboratory experiment*)
	Study population	Description of the participants, specified for all groups included
	Sample size	Number of units in the sample that were used for the analysis in the study for all groups included (*n = … , after exclusion, specified for all groups*)
	Eligibility: Inclusion criteria Exclusion criteria	Inclusion criteria for the sample Exclusion criteria for the sample
3. Intervention characteristics	Type	Description of the intervention (including all components) as described in the paper, as detailed as possible
	Aim	Aim of the intervention as described in the paper
	Duration	Duration of the total intervention
	Measurements	Description of the number of outcome measurements and when they are performed (e.g. multiple measurements for a pre-post design or longitudinal studies)
4. Main outcome variables relevant for the objectives	Health behavior: Domain Main outcome variable Secondary outcomes	Four categories are predefined: healthy eating, physical activity, tobacco use or alcohol consumption (*Choose: healthy eating, physical activity, tobacco use or alcohol consumption*) Description of the health behavior targeted (*Specify: Specific main outcome targeted*) Description of other outcomes targeted besides main outcome variable (*Specify: Specific secondary outcome(s) targeted*)
	Behavior Change Techniques: Separately mentioned? Type of BCTs used? How many used?	Are the specific BCTs mentioned in the paper? *(Was: Yes/no, Changed into: Yes, according to taxonomy, Yes, but not according to taxonomy (possible to code), Yes, but not according to taxonomy (not possible to code), Yes (some), according to taxonomy, Yes (some), but not according to taxonomy)* Description in the paper of the type of BCTs used in the intervention, using the taxonomy of BCTs *(Was: Specify: cluster and number according to taxonomy as described by authors, Changed into: Specify: cluster and number according to taxonomy as described by authors, at least for categories ‘Yes, according to taxonomy’, ‘Yes, but not according to taxonomy (possible to code)’)* Number of BCTs in the intervention as mentioned by the authors *(Was: Specify: number as mentioned by authors, Changed into: Specify: number (per intervention-arm) as mentioned by authors, at least for categories ‘Yes, according to taxonomy’, ‘Yes, but not according to taxonomy (possible to code)’)*
	Effectiveness: Low SEP groups? High SEP groups? What reported? Ineffectiveness (if applicable): what reasons are provided (Efficacy, Accessibility, Adoption, Adherence)?	Is the effectiveness of the intervention described among low SEP groups? (*Yes (eventually specifying if not exclusively low-SEP)/no*) Is a comparison made with a high SEP group? (*Yes (eventually specifying if not exclusively high-SEP)/no*) The information that is provided in the paper about the effectiveness among low (and eventually also high) SEP group (*Specify: copy description from paper (and indicate for which group) or NA*) If applicable and described, the reasons that are provided to explain why the intervention was not effective (*Specify: copy description from paper or NA*)
	Operationalization SEP: Objective indicators: Indicator(s) How specified for low SEP groups? If applicable, how specified for high SEP groups? Subjective indicators: Indicator(s) How specified for low SEP groups? If applicable, how specified for high SEP groups?	The objective indicator(s) used to describe SEP (*E.g.* *income, occupation, education, NA*) How is low SEP specified? (*E.g. (Grouping) score, level*) If included, how is high SEP specified? (*E.g. (Grouping) score, level*) The subjective indicator(s) used to describe SEP (*E.g.* *what scale(s) used, NA*) How is low SEP specified? (*E.g.* *cut-off score*) If included, how is high SEP specified? (*E.g.* *cut-off score*)
5. SEP-differences	Explanations SEP-differences in behavior change	If described, the reasons that are provided to explain why the intervention was differently effective for low and high SEP groups (*Specify: copy description from paper or NA*)
6. Other	Comments	Additional important information may be added here
7. Inclusion	Decision Reason for exclusion	Decision regarding in- or exclusion after reading full text (*Choose: Include or Exclude*) If exclusion, what is the reason in terms of eligibility criteria? (*Specify: reason in terms of eligibility criteria)*

Note: Descriptions of the extracted data (i.e. *specifications or examples in brackets*) are only provided when clarification is deemed necessary.

#### Synthesis of results

An overarching impression of the extracted data of all health behaviors is provided to address the research questions. That is, the operationalization of SEP and the (effectiveness of) applied BCTs according to SEP within the behavioral intervention literature are described across the four health behaviors. The results are presented visually (tables) and descriptively (narrative synthesis), and comprise frequencies, proportions, examples and citations.

## Results

### Selection of sources of evidence

**Figure 1.**
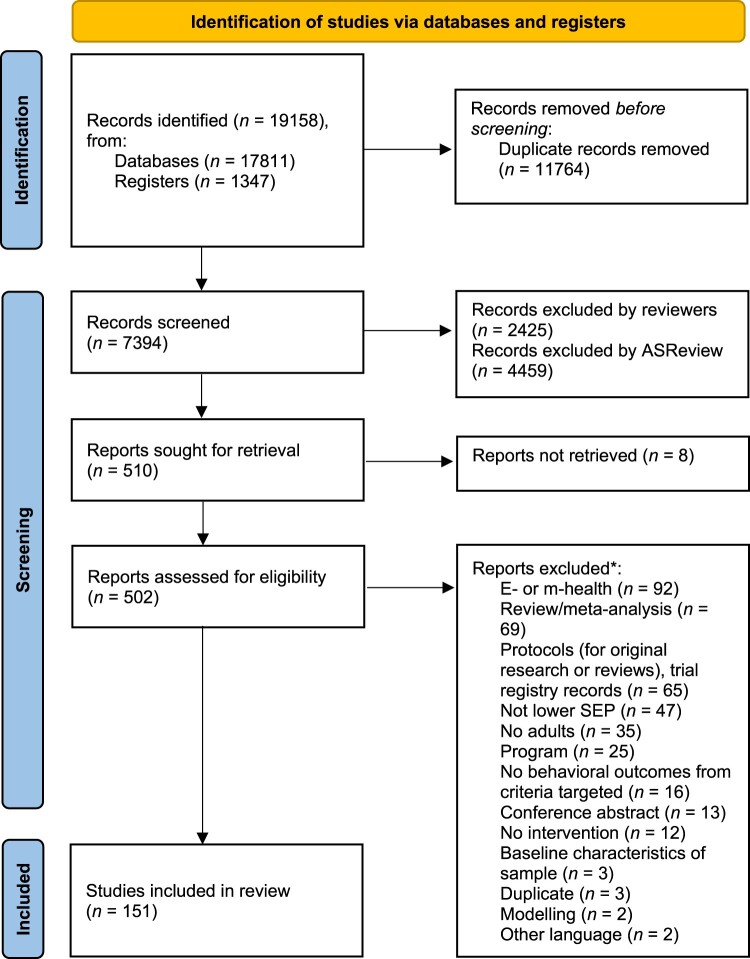
PRISMA flow diagram of the study selection process (*Page et al., 2021*). Note: Duplicates that were excluded during screening involved theses and dissertations that were also published in peer-reviewed journal articles, and were therefore missed during the process of identification. *Numbers do not tally, since some reports were excluded for multiple reasons.

The initial search yielded a total of 19,158 studies (see Figure 1). After removing duplicates and screening the titles and abstracts of initial records (*n* = 7439), the full-texts of 510 records were checked on availability and screened against the eligibility criteria. In total, 151 studies were identified for data extraction and included in this review (see ‘*’ in reference section, and for references of all included papers see Supplementary file 3).

### Characteristics of sources of evidence

Table 3 presents the key components of the included studies. Details of all papers can be found in Supplementary file 2 (sheet ‘Data extraction’).

**Table 3.** Key characteristics of sources of evidence.

Characteristic		*n* (%)
Study type	Original empirical research	143 (94.7)
	Secondary analyses	8 (5.3)
Country of origin (>1)	United States of America	83 (55.0)
	United Kingdom	22 (14.6)
	Australia	6 (4.0)
	The Netherlands	6 (4.0)
	Korea	4 (2.6)
	Brazil	3 (2.0)
	South Africa	3 (2.0)
	Canada	2 (1.3)
	Japan	2 (1.3)
	Norway	2 (1.3)
	Spain	2 (1.3)
	Other (included ones)	16 (10.6)
Publication year	2000–2005	14 (9.3)
	2006–2010	18 (11.9)
	2011–2015	35 (23.2)
	2016–2020	57 (37.7)
	2021–2022	27 (17.9)
Method	Quantitative	117 (77.4)
	Qualitative	17 (11.3)
	Mixed-Methods	17 (11.3)
Study design^a^	Experimental	
	Randomized controlled trial	29 (19.2)
	Randomized crossover design	2 (1.3)
	Randomized open-label clinical trial	1 (0.7)
	Randomized controlled pre-post	1 (0.7)
	Quasi-experimental	
	Pre-post with control	12 (7.9)
	Longitudinal with control	4 (2.6)
	Pre-post lag control group	1 (0.7)
	Open-label controlled study	1 (0.7)
	Intervention vs. control	1 (0.7)
	Serial cross-sectional surveys with control	1 (0.7)
	Pseudo-experimental	
	Pre-post without control	45 (29.8)
	Randomized trial	10 (6.6)
	Longitudinal	10 (6.6)
	Cohort study	4 (2.6)
	Non-randomized trial	1 (0.7)
	Open label study	1 (0.7)
	2×2×2 (without control)	1 (0.7)
	2×2 (without control)	1 (0.7)
	Cross-sectional	
	Interview	20 (13.2)
	Focus group	11 (7.3)
	Survey	8 (5.3)
	Field notes, logs and observations	8 (5.3)
Number of measurements^a^	1	31 (20.5)
	2	75 (49.7)
	3	35 (23.2)
	4	12 (7.9)
	5	3 (2.0)
	8	1 (0.7)
	Unknown	4 (2.6)
Target group^a^	Adults (excluding older adults and families)	138 (91.4)
	Resident of disadvantaged area	47 (31.1)
	Women	26 (17.2)
	Receiving or eligible for federal services or assistance (programs)	26 (17.2)
	Patients (e.g. at clinics)	24 (15.9)
	Customers or main household shopper	16 (10.6)
	Older adults	9 (6.0)
	Men	7 (4.6)
	Parents or caregivers	6 (4.0)
	Pregnant women	6 (4.0)
	Workers	5 (3.3)
	Clients (e.g. of homeless shelter)	5 (3.3)
	Families/households	5 (3.3)
	Mothers	3 (2.0)
	Experiencing food poverty	3 (2.0)
	Fathers	2 (1.3)
Ethnicity target group^^^	African American	18 (11.9)
	Multi-ethnic	11 (7.3)
	Hispanic	5 (3.3)
	Latino	3 (2.0)
	Asian	3 (2.0)
	Mexican American	1 (0.7)
Intervention duration	One occasion – 2 months	58 (38.4)
	> 2 months – 6 months	43 (28.5)
	> 6 moths – 7 years	28 (18.5)
	Unknown	22 (14.6)
Targeted health behavior^a^	Healthy eating	110 (72.8)
	Physical activity	66 (43.7)
	Tobacco use	29 (19.2)
	Alcohol consumption	10 (6.6)
Number of health behaviors targeted	1	98 (64.9)
	2	43 (28.5)
	3	6 (4.0)
	4	4 (2.6)

Notes: When interventions targeted families, only the results regarding parents were abstracted. For longitudinal studies, number of measurements includes baseline measure.

^a^ Numbers and percentages of study design, number of measurements, target group and targeted health behavior do not tally (up to 151 papers, 100% in total), as their categories could be present more than once per study (e.g. for mixed-methods or interventions targeting women living in disadvantaged areas).

^^^ Not all studies targeted their intervention at a specific ethnic group, hence these numbers and percentages do not add up to 100%.

#### Study characteristics

Most studies were performed in Western countries (mainly United States of America, followed by United Kingdom, Australia and the Netherlands), described original empirical research (*n* = 143, 94.7%), and used a quantitative study design (*n* = 117, 77.4%, mainly pre–post measure designs and randomized controlled trials).

#### Participant characteristics

Participants (*n* ranging from 5 to 132,586) that were targeted by the interventions were mainly residents of disadvantaged areas and predominantly of African American ethnicity (if reported). Most studies (*n* = 121, 80.1%) were exclusively targeted at participants with lower SEP. A small proportion of studies compared participants with lower SEP to those with a relatively higher SEP (i.e. 24 to high SEP groups and six to relatively higher SEP, such as medium and high SEP groups grouped together; Parks et al., 2017; Pettigrew et al., 2016). For more information regarding participants’ SEP, see the section ‘The operationalization of SEP’ below.

#### Intervention characteristics

The type, duration and intensity of interventions varied greatly between studies. Interventions consisted, for example, (both solely or combined) of classes or workshops (e.g. Dickin et al., 2014; Hand et al., 2014; Nieves et al., 2022), policy implementations (e.g. tobacco price increases or sugar-content-based taxes; Essman et al., 2021; Kim & Cho, 2022; Tabuchi et al., 2017), cognitive or behavioral counseling (e.g. Jenum et al., 2009; Steptoe et al., 2004), therapy (e.g. pharmacotherapy, cognitive behavioral therapy or nicotine replacement therapy; Darker et al., 2022; Landais et al., 2021; Webb Hooper et al., 2017) or environmental changes (e.g. nudges or neighborhood walking route; Huitink et al., 2020; Prins et al., 2019). Most interventions had a relatively short duration of one occasion up to two months and interventions differed in the number of contact moments provided (e.g. 6-week interventions consisting of weekly one-hour education classes versus sessions of 15 hours in total; Backman et al., 2011; West et al., 2020). Generally, the included studies covered a great variety of these intervention characteristics.

#### Behavioral outcome characteristics

Most interventions promoted one (*n* = 98, 64.9%) or two health behaviors (*n* = 43, 28.5%). Overall, interventions mainly targeted healthy eating (*n* = 110, 72.8%, e.g. daily fruit and vegetable or fat intake; Anjali, 2013; West et al., 2020), followed by physical activity (*n* = 66, 43.7%, e.g. self-reported weekly activity or objectively measured step count; Bopp et al., 2009; Whitehead et al., 2007). The reduction of tobacco use (*n* = 29, 19.2%, e.g. number of cigarettes smoked or smoking status; Landais et al., 2021; Tabuchi et al., 2017) and especially alcohol consumption (*n* = 10, 6.6%, e.g. daily alcoholic beverage consumption or weekly glasses of alcohol consumed; Klassen et al., 2008; Waller et al., 2016) were promoted less often.

### Synthesis of results

#### The operationalization of SEP

SEP was operationalized in divergent ways. All studies used objective indicators for SEP, but a common approach to objectively identify SEP groups could not be identified within the literature. That is, various objective SEP-indicators were used, with income, education, neighborhood characteristics and occupation being used most frequently (see Table 4). Within these clusters of indicators, different operationalizations were identified. For example, various measures were used as a proxy for neighborhood characteristics, such as index scores (e.g. Ball et al., 2016; Huitink et al., 2020), the median household income of residents of selected communities and poverty levels (e.g. Einterz, 2017; Walker et al., 2018), household car ownership (e.g. Klassen et al., 2008), limited education or high unemployment rates among residents (e.g. Mier et al., 2011) or lack of medical services (e.g. Mier et al., 2011). The indicators also differed in terms of their measurement level. Income, for example, was described by individual (e.g. net income or weekly income; Jenum et al., 2009; Perkins-Porras et al., 2005), household (e.g. equivalent household income or monthly household income; Tabuchi et al., 2017; Whitehead et al., 2007) or community levels (e.g. Essman et al., 2021). Only five studies (3.3%) also included subjective SEP-indicators (see Table 4), such as the MacArthur scale of subjective social status (Voigt et al., 2022). Thus, different objective indicators were used to operationalize SEP and, even if similar indicators were used, operationalization and measurement approaches varied.

**Table 4.** Operationalization SEP.

Characteristic	Category	*n* (%)
Objective indicators^a^	Income	92 (60.9)
	Education	75 (49.7)
	Neighborhood characteristics	52 (34.4)
	Occupation	37 (24.5)
	Eligible for or receiving assistance (program, prescriptions)	11 (7.3)
	Food poverty/security	4 (2.6)
	Material possessions and lifestyle	3 (2.0)
	Composite score	2 (1.3)
	Living situation/standard	2 (1.3)
	Class (social or socio-economic)	2 (1.3)
	Housing	2 (1.3)
	Health insurance status	2 (1.3)
	Meeting basic expenses	1 (0.7)
	Unknown	1 (0.7)
Subjective indicators^a^	MacArthur scale of subjective social status	1 (0.7)
	Quebec Health and Social Survey question ‘perceived economic situation compared to people of similar age’	1 (0.7)
	Perceived household economic status	1 (0.7)
	Perceived financial strain	1 (0.7)
	Perceived current financial situation*	1 (0.7)
	Perceived job strain*	1 (0.7)
	Perceived social capital at work*	1 (0.7)
	Perceived neighborhood safety*	1 (0.7)

Note: ^a^ These numbers and percentages do not tally, since multiple (both objective and subjective) indicators could be used within one study.

* These subjective indicators were all measured within one study (Sorensen et al., 2007).

Regardless of what indicator was used to operationalize SEP, studies varied widely in whether they defined ‘low’ SEP and, if so, in how such definitions were applied. Within a substantial proportion of studies it was not clearly defined what was perceived as ‘low’ SEP. Often, the target group or setting was described in terms related to lower SEP, such as ‘(socio-economically) disadvantaged’ (e.g. Realmuto et al., 2018; Visram et al., 2014; Petersen et al., 2013), ‘low-income’ (e.g. Hamilton, 2016) or ‘deprived’ (e.g. Forde & Solomon-Moore, 2019), while no definition was provided of what these terms mean. In such cases, it was unclear what criteria participants or settings exactly meet (e.g. when is someone perceived as ‘socio-economically disadvantaged’?). Also, if definitions of ‘lower SEP’ were provided, they varied a lot between studies. To describe low-income participants, studies for example applied a threshold of <100% (e.g. Nieves et al., 2022), ≤185% (e.g. Bird & McClelland, 2017; Dollahite et al., 2014; Seguin-Fowler et al., 2021; Skalka, 2020), <200% (e.g. Hayashi et al., 2010; Ritten et al., 2016) or <250% (e.g. Gray et al., 2021) of the federal poverty line. In addition to these different thresholds, studies also varied what proportion of the included sample fell into the definition of ‘lower SEP’ in order to justify labeling a sample overall as (relatively) low SEP. To illustrate, the percentage of the sample that involved people with low educational level was 22% in one study (Jenum et al., 2009), but included 32% (Opie et al., 2020) or 44% (Hankonen et al., 2009) in other papers, while all of these papers referred to this sample as a ‘low SEP sample’. Thus, definitions of ‘low’ SEP were often lacking and, if provided, their application varied considerably between studies.

Finally, often no data was provided that substantiated the definition or description of a ‘low’ SEP group. Studies that applied an eligibility criterion of having an income at or below a certain percentage of the federal poverty line, for instance, often did not report participants’ actual incomes (e.g. Dollahite et al., 2014; Nieves et al., 2022). Other studies also depicted the target group in terms of certain SEP-indicators (e.g. ‘low-income families eligible for or receiving food stamps’ or ‘low-income older adults living in a senior housing facility’; Townsend, 2005, p. 21; Resnick et al., 2009, p. 353 respectively), while it was not supported with any numbers. In addition, studies regularly described the sample in terms of one indicator (e.g. low-income parents or residents of a low-income neighborhood), while (also) other indicators were measured (e.g. educational level; Nieves et al., 2022; Dickin et al., 2014). Thus, it remains unclear what levels of certain indicators participants exactly have. Taken together, SEP is predominantly described with objective indicators, but approaches for measuring, defining and substantiating ‘low’ SEP vary within the literature.

#### The specification of BCTs

Descriptions of interventions varied extremely in their level of detail and the extent to which techniques were specified. Less than half of studies (*n* = 63, 41.7%) described techniques for the entire intervention. Of these, only four (6.3%, of which two studies described the same intervention; Ball et al., 2016; Opie et al., 2020) specified techniques according to the BCT taxonomy v1 (Michie et al., 2013) and reported details regarding the BCTs (i.e. when or how they are applied or the determinants they target; Ball et al., 2016; Coupe et al., 2022; Gray et al., 2021; Opie et al., 2020)^3^. For example, the application of the BCTs ‘social support (emotional and practical)’ was described as ‘community health workers provided social support to participants and encouraged family members and other social network members to help participants by supporting lifestyle changes and medication taking, attending clinic visits, and providing emotional support’ (Gray et al., 2021, p. 2146). Some interventions described all BCTs that were applied within each intervention component, thereby also outlining how many times techniques were used (Ball et al., 2016; Coupe et al., 2022; Opie et al., 2020). Besides, some studies reported the reasons for applying certain BCTs specifically among the target group of people with lower SEP (Ball et al., 2016; Coupe et al., 2022; Opie et al., 2020). It is mentioned, for example, that efforts were made to incorporate various BCTs particularly regarding goal setting as it appeared promising in previous nutrition interventions (Ball et al., 2016; Opie et al., 2020). Thus, for less than half of the studies, interventions are described on specific technique-level and only four studies used the BCT taxonomy to systematically report which, how often and/or why specific BCTs are implemented within the intervention.

Of the 59 studies that described interventions on technique-level but not according to the BCT taxonomy, the review team was able to code all techniques according to the taxonomy of 32 of these studies. This resulted in 33 different BCTs identified within the interventions described in 36 studies (with four studies using the taxonomy and 32 studies for which BCTs were coded by the review team, see Table 5). The number of techniques applied within interventions varied. The lowest number of BCTs used in an intervention was one (single-component interventions: e.g. Ni Mhurchu et al., 2012; Giles et al., 2016), while the largest number of different BCTs used within multi-component interventions was 20 (Ball et al., 2016; Opie et al., 2020). BCTs from 15 different categories were implemented, with techniques from the categories ‘Goals and planning’, ‘Antecedents’, ‘Reward and threat’, ‘Social support’ and ‘Shaping knowledge’ being the most frequently applied. The most frequently reported BCTs were ‘Goal setting (behavior)’ (*n* = 12), ‘Restructuring the physical environment’ (*n* = 12) and ‘Instruction on how to perform the behavior’ (*n* = 10). Thus, less than half of studies described techniques for the entire intervention, and among those where all techniques were identified according to the BCT taxonomy various BCTs were applied.

**Table 5.** Identification of BCTs used within interventions.

BCT cluster and number	BCT number	*n*
**1. Goals and planning**		**36**
	1.1. Goal setting (behavior)	12
	1.2 Problem solving	6
	1.4 Action planning	9
	1.5 Review behavior goal(s)	6
	1.6 Discrepancy between current behavior and goal	2
	1.8 Behavioral contract	1
**2. Feedback and monitoring**		**5**
	2.2 Feedback on behavior	1
	2.3 Self-monitoring of behavior	4
**3. Social support**		**12**
	3.1 Social support (unspecified)	7
	3.2 Social support (practical)	4
	3.3 Social support (emotional)	1
**4. Shaping knowledge**		**11**
	4.1 Instruction on how to perform the behavior	10
	4.2 Information about antecedents	1
**5. Natural consequences**		**6**
	5.1 Information about health consequences	4
	5.3 Information about social and environmental consequences	2
**6. Comparison of behavior**		**5**
	6.1 Demonstration of the behavior	4
	6.2 Social comparison	1
**7. Associations**		**3**
	7.1 Prompts/cues	3
**8. Repetition and substitution**		**9**
	8.1 Behavioral practice/rehearsal	6
	8.2 Behavior substitution	3
**9. Comparison of outcomes**		**2**
	9.1 Credible source	2
**10. Reward and threat**		**14**
	10.1 Material incentive (behavior)	7
	10.4 Social reward	2
	10.7 Self-incentive	2
	10.9 Self-reward	3
**11. Regulation**		**4**
	11.1 Pharmacological support	1
	11.3 Conserving mental resources	3
**12. Antecedents**		**20**
	12.1 Restructuring the physical environment	12
	12.2 Restructuring the social environment	2
	12.5 Adding objects to the environment	6
**13. Identity**		**2**
	13.1 Identification of self as role model	2
**14. Scheduled consequences**		**1**
	14.3 Remove reward	1
**15. Self-belief**	-	-
**16. Covert learning**		**1**
	16.2 Imaginary reward	1

Notes: This table demonstrates how many times BCTs (Michie et al., 2013) are applied at least ones within interventions described in the studies where BCTs were identified (described by the authors or coded by the review team) for the complete intervention. Some of those 36 studies described multiple interventions, hence the BCTs of 41 interventions are outlined in this table. When a BCT is applied multiple times within an intervention, *n* is coded as one. BCTs that do not occur within interventions are left out of this table.

Still, the majority of studies did not describe the intervention (completely) at technique-level. Of those, most studies (*n* = 72, 47.4%) described some techniques for parts of the intervention (regardless of whether this was done according to the BCT taxonomy or not). In these cases, examples of techniques were provided but their application was not further specified (i.e. how or how many times) or the applications of only some techniques were mentioned, while a specific description for all components of the intervention was lacking. One study, for example, provided a description of a BCT applied within the intervention: ‘participants were also encouraged to self-monitor smoking habits … ’ (Katz et al., 2008, p. 4). Here, it remains unknown how the technique was exactly applied (e.g. which self-monitoring techniques were promoted). In a similar vein, goal setting was often mentioned as one of the components (e.g. ‘participants were asked to establish individual goals after the completion of each session’; Gallegos et al., 2021, p. 86), but the type of goal setting could not be identified (e.g. goal setting regarding a behavior or outcome; Michie et al., 2013). As only some components of interventions are specified in terms of techniques, the total package of components with which interventions aim to foster behavior change remains unclear.

These interventions that did not specify all techniques were in some cases described by the theoretical basis that guided the development of the intervention, such as the transtheoretical model (or ‘stages of change’; Prochaska & Velicer, 1997), self-determination theory (SDT; Ryan & Deci, 2008), social cognitive theory (Bandura, 1986) and socio-ecological models (e.g. Contento, 2010; McLeroy et al., 1988). Such theoretical basis was often used to describe the content of different activities, sessions or materials. The sessions of a physical activity intervention, for example, were based on need-supportive strategies according to the tenets of SDT, such as relatedness (e.g. group-based sessions), competence (e.g. providing activities with different levels and intensity) and autonomy (e.g. facilitating decision-making) supportive strategies (Sanz-Remacha et al., 2023). In line with such theoretical underpinnings, studies also reported intervention content in terms of the determinants proposed to target. For example, ‘ … a personal letter tailored to their attitude, self-efficacy, social norm and stage of change’ (Siero et al., 2000, p. 638). Taken together, almost half of the papers do not provide a complete overview of the BCTs used in the intervention, but rather describe some BCTs possibly along with the overarching theoretical basis of the intervention.

Finally, a small proportion of studies did not specify any techniques at all (*n* = 16, 10.6%). Such papers mainly described interventions in general terms and information regarding specific intervention content and strategies was lacking. Hence, these interventions were described according to the activities carried out, the materials used and the topics addressed. Examples of such descriptions include ‘the necessary training to improve the behaviors of health promotion … using lecture, slides, questions & answers, group discussion every other day in the afternoon in six sessions for 60-60 [*sic*] minutes’ (Majlesi et al., 2018, p. 334), and

the mass media campaign … was centered around energy balance. Two campaign waves were scheduled between the four measurements … All campaign efforts were based on energy intake, energy expenditure and caloric compensation strategies. Each campaign wave, however, was designed to be targeted to a different target group. As a result, the campaign waves differed in terms of content and style (Verheijden et al., 2012, pp. i76-i77).

In such cases, the actual content of the different intervention components was not described and no techniques were specified.

#### The effectiveness of interventions and BCTs

In total, 144 (95.4%) studies reported the effectiveness of 175 intervention arms (i.e. this involves studies testing the effectiveness of multiple interventions). The majority described the effectiveness of intervention arms regarding interventions involving multiple components (*n* = 139 multi-component intervention arms). Hereby, it is described whether an intervention was effective in improving the health behavioral outcomes among the target group, while no distinctions were made between the techniques and their unique effectiveness. This also held for studies that clearly described how many and frequently techniques were applied within an intervention. For example, the effectiveness of the ‘ShopSmart 4 Health’ intervention that involves 20 BCTs (of which some are applied multiple times during the intervention), was described as ‘no significant intervention effects on vegetable or fruit purchasing … Participants in the behavior change intervention arm reported consumption of significantly more vegetables … there was no intervention effect on reported fruit consumption’ (Ball et al., 2016, p. 436). Thus, most papers that report on effectiveness do so based on a combination of techniques aggregated together.

Some interventions, however, consisted of a single BCT and therefore can report on effectiveness of specific techniques among the target group (*n* = 36 arms; e.g. Armitage & Arden, 2010; DeBiasse, 2016; Evoy et al., 2019). While for all these single-component interventions it was clear what techniques were evaluated, not all could be labeled according to the BCT taxonomy by the review team (e.g. price discounts, taxes and providing vouchers). Of the interventions that clearly tested the unique effect of a BCT according to the taxonomy, the effectiveness of ‘Restructuring the physical environment’ was tested within eight intervention arms and the effectiveness of ‘Material incentive (behavior)’ in five^4^. ‘Restructuring the physical environment’ had mixed effects, as it significantly increased behavioral outcomes in the proposed, desired direction eight times, in the undesired direction one time and not at all 14 times^5^. To illustrate, a neighborhood walking route significantly increased weekly time spent walking over nine months in total and for reasons of utility, but not for recreational walking among older adults residing in deprived neighborhoods (Prins et al., 2019) and switching to a supercenter due to the closing of a local grocery store desirably increased dietary intakes such as fruit and vegetables, but also undesirably increased intake of alcohol among residents of a rural high-poverty county (Gillespie et al., 2022). Also ‘Material incentives (behavior)’ had mixed effects on behavioral outcomes (all regarding tobacco use), as it significantly increased outcomes in a desired direction four times, but had no effects at all three times^6^. For example, integrating draws from a prize bowl for each negative carbon monoxide sample into standard smoking cessation care significantly increased the number of negative samples submitted and the duration of consecutive abstinence at four weeks post-intervention, but not at follow-up measures after four weeks among homeless smokers (Rash et al., 2018). For details of all effects on behavioral outcomes, see Supplementary file 2 (sheet ‘Data extraction’).

Few studies compared the effectiveness of interventions among different SEP groups, and – if tested at all – the direction of the effects varied. In total, 29 (19.2%) studies reported the moderating effects of SEP 225 times (i.e. this involves studies testing SEP-moderation effects of multiple interventions, behavioral outcomes and SEP-indicators). Of these, about 70% of time (*n* = 156) behavioral outcomes were similar for both SEP groups (121 compared to high-SEP, 35 compared with a relatively higher SEP group). Furthermore, slightly less than 20% of time (*n* = 41) the behavioral outcomes increased more among people with relatively lower SEP (32 outcomes compared with high-SEP, nine with relatively higher SEP). Lastly, over 10% of time (*n* = 28) behavioral outcomes increased more among relatively higher SEP groups (23 outcomes compared with high-SEP, five with relatively higher SEP). Reasons provided for the diverging effects in favor of lower SEP groups were, for example, ‘our study encouraged social capital and exercises that could be conducted near participants’ homes without causing a financial burden’ (Saito et al., 2021, p. 5) and ‘low-SEP individuals potentially respond to cigarette excise taxes in a disproportionately strong manner’ (Parks et al., 2017, p. 213). The increased effectiveness among higher SEP groups was explained by some studies in terms of financial disparities: e.g.‘healthy foods are more costly and less available in low-income communities, thus limited financial resources can be a significant barrier to making diet-related lifestyle changes’ (Williams et al., 2021, p. 5) and ‘because financial concerns make life even more stressful, activities other than those related to basic needs are not likely to be a priority for low-income participants’ (Nour et al., 2006, p. 10). Thus, limited studies examine the moderating effect of SEP and when tested, studies report diverging effects and various explanations in terms of tailoring, access and adoption.

## Discussion

### Summary of evidence

This scoping review aimed to describe what is known in the current literature about the BCTs applied within behavior change interventions and their effectiveness to encourage healthy eating and physical activity, and reduce tobacco use and alcohol consumption among individuals with lower SEP or when comparing lower versus higher SEP groups. By scoping 151 studies, we aimed to outline how SEP is operationalized and which objective and subjective indicators are used to measure SEP, how BCTs are studied within the current behavior change literature according to SEP and – if specified – how many and what type of BCTs are used, what is reported about the effectiveness of behavior change interventions and their BCTs, and whether studies are targeted at low SEP groups alone or if a comparison is made with a relatively higher SEP group and what the results are of such comparison.

There are four main findings that arise from this scoping review. The first is that a common approach to operationalize SEP is lacking within the current behavior change literature. In line with previous reviews, we were unable to identify a standard method for operationalizing SEP within current interventions. That is, SEP was assessed with various (mainly objective) indicators (Bull et al., 2014; Harakeh et al., 2024; Moore et al., 2015; Ronteltap et al., 2022; Western et al., 2021), measured on various levels (e.g. individual, household or neighborhood; Harakeh et al., 2024; Shagiwal et al., 2020), not always specifically defined (Anselma et al., 2020; Harakeh et al., 2024) and if so, defined in various ways and by varying thresholds (Bull et al., 2014). On top of that, we found that often no data was provided to substantiate the description of a ‘low’ SEP group. Overall, scholars use many different approaches, often without or with little justification for the choice of a particular SEP-measure or its validity (Antonoplis, 2023; Braveman et al., 2005; Moore et al., 2015). It therefore remains unclear what is actually perceived as ‘lower’ SEP, and regardless of the reported effectiveness and level of specificity regarding BCTs, this in and of itself already limits our ability to build evidence regarding effective interventions particularly for lower SEP groups. Such variation in deriving SEP can influence the understanding of the associations between SEP characteristics and health outcomes and may result in contradictory results about these relationships (Antonoplis, 2023; Braveman et al., 2005; Shavers, 2007). In addition, it is remarkable that subjective SEP-indicators are used seldomly, since they are found to robustly predict health outcomes and to be associated with health even after controlling for objective SEP-indicators (e.g. Adler et al., 2000; Operario et al., 2004; and for a review, see Kraft & Kraft, 2021).

Secondly, most intervention content is described on a general, overarching level, since no or only some techniques are described for the interventions. This finding is consistent with previous reviews indicating that intervention descriptions are brief and not always detailed, due to underreporting of the active BCT components (Anselma et al., 2020; Bull et al., 2014; 2018; De Bruin et al., 2021). Besides, if interventions are completely described on technique-level, only a limited number of studies uses a systematic approach such as the behavior change taxonomy (Michie et al., 2013) and descriptions about how frequently BCTs are applied within an intervention are often lacking (e.g. goal setting appeared to be one of the most frequently applied BCTs, but it is often not mentioned whether it is applied multiple times). Logically, this is partly because we included studies that were published before the BCT taxonomy was published. Still, it remains unclear for most interventions what exactly is attempted to perform among the target group as it is often not reported which, how often and/or why specific BCTs are implemented.

Thirdly, and related to the second main finding, the effectiveness of interventions is mainly described for interventions as a whole, involving all components. This is in line with previous reviews reporting that most studies test the effectiveness of highly diverse interventions, incorporating multiple techniques (e.g. Anselma et al., 2020; Harakeh et al., 2024; Michie et al., 2009; Ronteltap et al., 2022; Shagiwal et al., 2020). Despite this being understandable in terms of practical feasibility of performing research, it does limit our ability to build evidence regarding which techniques work specifically among lower SEP groups. To illustrate, if the combination of multiple techniques (up to 20; e.g. Ball et al., 2016; Opie et al., 2020) is tested, it can only be concluded whether this specific combination of techniques works or not. One cannot learn which techniques contributed most to the intervention success or which techniques were negligible or even redundant. It could be that interventions effectiveness is associated with the number of techniques (other reviews are inconclusive; e.g. Harakeh et al., 2024; Michie et al., 2009; Ronteltap et al., 2022), and that effectiveness may be driven primarily by the presence or combination of specific BCTs (as demonstrated among the general population; Dusseldorp et al., 2014).

The fourth main finding is that relatively few studies compare the effects of interventions (and their techniques) across different SEP groups, which was also indicated by previous reviews (Moore et al., 2015; Ronteltap et al., 2022). Our review indicates that many studies test the effectiveness of behavioral interventions targeting low SEP groups specifically, which importantly contributes to knowledge about how to effectively promote health behavior among the people that need it most (e.g. Pampel et al., 2010; Probst et al., 2020). However, to learn how we can combat intervention-generated health inequalities, comparing the effectiveness across different SEP groups is required within interventions aimed at the general population level. As most studies were targeted at lower SEP groups only and limited studies targeted a broader population, the divergent effects for people with relatively lower and higher SEP are mostly not compared. It therefore remains unclear whether interventions increase, decrease or do not alter existing health inequalities. Nevertheless, the limited studies within this review that did include SEP as a moderator reported a range of SEP gradients in effects, which is in line with previous reviews demonstrating mixed results (Moore et al., 2015). Thus, researchers should be aware that interventions, if effective at all, may have diverging effects for people among different SEP groups.

Beside these main findings, it is noteworthy that most interventions stimulated healthy eating (73%) or physical activity (44%). Interventions stimulating smoking cessation and lower alcohol consumption are scarce (19% and 7% respectively). This is remarkable, as all four health behaviors follow a socio-economic gradient with people from relatively lower SEP groups engaging more often in unhealthy behaviors and being more negatively affected by their effects than people with higher SEP (Collins, 2016; Pampel et al., 2010; Probst et al., 2020). In addition, all four health behaviors substantially explain the relationship between SEP and mortality (Gruer et al., 2009; Hart et al., 2011; Nandi et al., 2014; Stringhini et al., 2010; Whitley et al., 2014). As rates of smoking and binge drinking are still higher among lower SEP groups, these behaviors similarly deserve attention from intervention developers and scholars.

### Implications for research and practice

Together, this all seems to limit our ability to accumulate evidence as it can lead to divergent conclusions regarding the effectiveness of interventions and their techniques according to SEP. It therefore remains unclear what actually works for people from different SEP groups in promoting health behavior. To build systematic evidence, we propose several recommendations for future research and practice that originate from our findings. Regarding the reporting of behavior change interventions in general, we first recommend scholars to deliberately include a justification in research reports of how (lower or higher) SEP is conceptualized (i.e. measured, defined and substantiated). It is also important to systematically operationalize SEP (or broader dimensions of health inequity) in future studies according to existing frameworks for health inequalities (e.g. PROGRESS-Plus; O'Neill et al., 2014, or Conceptual Framework for Action on the Social Determinants of Health; Solar & Irwin, 2010) or by developing new and refining existing frameworks for SEP more specifically. In a similar vein, intervention content should be described more systematically and in greater detail (for an overview, see Johnston, 2014), by following existing reporting guidelines (e.g. CONSORT 2010 or TIDieR; Hoffmann et al., 2014; Schulz et al., 2010) and behavior change taxonomies (e.g. BCT taxonomy or intervention mapping approach; Michie et al., 2013; Kok et al., 2016). We agree with the recommendation of a previous review (Shagiwal et al., 2020) that providing a checklist of the BCTs used in future studies would help to identify active BCTs and to develop effective and replicable interventions.

We also have specific recommendations for future studies to address existing knowledge gaps. Firstly, we recommend that future research could consider the unique and combined effectiveness of BCTs (in line with Ronteltap et al., 2022). One way to build such evidence, is by empirically testing the effectiveness of single-component interventions. However, as achieving and maintaining health behavior change is difficult, and especially for people with lower SEP, interventions involving multiple BCTs are required. Therefore, meta-analyses could be performed to examine which unique BCTs or particular combinations of BCTS are effective. A prerequisite for meta-analyses is that BCTs have been extensively reported using standardized methods in individual studies (one of our recommendations), an important practice that in current meta-analyses is often referred to as insufficiently performed (e.g. Shagiwal et al., 2020). Secondly, to learn what works for whom (Michie et al., 2009; Rothman & Sheeran, 2020), it is essential that scholars do not only test the effects of interventions and BCTs solely among relatively lower or – what is mostly done in current psychological research – higher SEP groups, but that they also examine SEP-moderation effects. This can be achieved by empirical studies that are powered for detecting moderation effects or by re-analyzing representative samples of existing intervention data (e.g. Czwikla et al., 2021). Building further on such insights, future research could examine whether interventions and their BCTs affect the hypothesized MoAs in similar ways among lower and higher SEP groups (Michie et al., 2009). Besides intervention efficacy, differences in access, adoption and tailoring should also be considered (in both future empirical studies and reviews) as these may depend on SEP and may influence inequalities (e.g. Bukman et al., 2014; Estabrooks et al., 2003; Szinay et al., 2023; Van Oort et al., 2005; Veinot et al., 2018). Lastly, future interventions among different SEP groups can extend their focus by stimulating a broader range of health behaviors. This may all help to gain knowledge about how health behavior can be effectively promoted according to SEP, hence to combat existing health inequalities and improve health for all.

### Strengths and limitations

This scoping review has several strengths. We believe that a comprehensive overview was provided of the current literature that is relevant for today’s society. That is, an elaborate search strategy warranted that many studies were included that were published from 2000 to 2022, ranged in their methodology (both quantitative, qualitative and mixed-methods) and described interventions promoting (at least one of) four health behaviors among people with lower (eventually compared with higher) SEP, regardless of how SEP was operationalized within studies. In this sense, it was possible to detect a wide range of sources of evidence, to include different types of research (e.g. with different study designs) and to warrant an extensive synthesis of the literature, which is important for broadly scoping relevant empirical evidence on (the effectiveness of) BCTs of health behavior interventions among people with lower SEP.

Nevertheless, the current findings need to be interpreted considering some limitations. First, this review focused on behavior change interventions and techniques aiming to foster individual health behavior change. Although such interventions may encompass contextual strategies, research regarding structural changes in the distribution of resources and power (e.g. improving social security or basic income) has not been included while such policy interventions are deemed necessary to tackle health inequalities (e.g. Dijkstra & Horstman, 2021; McCartney et al., 2013). Secondly, the current review addressed socio-economic health inequalities, and specifically addressed studies focusing on the core objective indicators of SEP (e.g. income, education, occupation and place of residence) and subjectively experienced social status. It thereby did not include studies that focus on other factors along which one can stratify health outcomes, such as ethnicity, gender (identity) or age. Current conclusions regarding the operationalization of SEP should therefore be perceived within the context of these specific objective and subjective indicators of SEP. However, health equity goes beyond (these indicators of) SEP and a broader approach of health inequity (e.g. O'Neill et al., 2014; Solar & Irwin, 2010) may extend our findings. Thirdly, as many included studies poorly reported intervention content and (if reported at all) described activities and techniques not (always) in detail, coding the BCTs was often difficult. Besides, BCTs that are more familiar could have been more easily coded than less well-known BCTs. This difficulty in coding BCTs has also been reported by other reviewers (e.g. Bull et al., 2018; Ronteltap et al., 2022; Shagiwal et al., 2020). The uncertainty in retrospectively classifying BCTs also limits interpretation of potential SEP-differences in intervention effects. Fourthly, as it was out of scope of the current review, we did not perform a critical appraisal of the included studies and can therefore not assess their individual quality while it may vary between studies (Bull et al., 2014; Ronteltap et al., 2022; Western et al., 2021). Fifthly, the third step of the three-step search strategy recommended by JBI was not performed for feasibility reasons, hence some additional relevant papers may have been missed. Lastly, since the review team has good command in Dutch and English but not in other languages, only papers that were published in these languages were eligible and finally only English papers were included for data extraction. This could have affected the variety of the origins of sources, since the largest proportion of studies was performed in relatively high-income, Western countries (e.g. United States of America, United Kingdom). This may have limited the characteristics and SEP-levels of the participants within the included studies, although the studies were conducted in all continents, contributing to the generalizability of the current findings.

### Conclusions

This scoping review provided insight into the techniques that are applied within current behavior change interventions and their effectiveness to alter physical activity, unhealthy eating, tobacco use and alcohol consumption among people with lower (versus higher) SEP. Within the current behavior change literature, no common approach could be identified of operationalizing SEP, describing interventions and their BCTs and examining their effectiveness according to SEP. This impedes a systematic evidence accumulation regarding which (combinations of) intervention components are effective among different SEP groups. To systematically build evidence, future intervention research should justify what is perceived as lower or higher SEP, and take into account the possible diverging effects for different SEP groups by examining the moderating effects of SEP. In addition, more specific studies are needed where BCTs are systematically described according to a behavior change taxonomy (e.g. Michie et al., 2013) so that their unique (and combined) effectiveness among different SEP groups can be examined within experimental, empirical research or meta-analyses. Only then it can be learned which building blocks of behavior change interventions are useful for the development of more effective interventions for all individuals within society. This is crucial to tackle existing SEP-differences in overall health and health behaviors and to prevent intervention-generated inequalities.

## Supplementary Material

Supplemental Material

Supplemental Material

Supplemental Material

## Data Availability

The data that support the findings of this study are available within the article, its supplementary materials and the Open Science Framework, at https://osf.io/25eyg/.

## Notes

1 Given the large reach of the main search, we decided to not scan the reference lists of relevant reviews and meta-analyses to check whether relevant original research papers were missed by the initial search (contrary to our preregistration).

2 The main search strategy was extensive in both scope and complexity (lowering the chance of finding additional relevant studies with additional reference searching; Horsley et al., 2011) and resulted in a large number of studies included. Therefore, we deemed the main search strategy to be comprehensive and the third step of the three-step search strategy recommended by JBI (i.e. backward and forward reference searching) was not performed (contrary to our preregistration).

3 This can in part be explained by the fact that the BCT taxonomy v1 has been published in 2013 (Michie et al., 2013), while this review includes papers published from January 2000. Still, 42 out of the 63 papers that completely described interventions at technique-level (66.7%) are published from 2014 onwards, and of these only four (9.5%) specified techniques according to the taxonomy. Two of these four studies that used the BCT taxonomy to describe intervention content (Ball et al., 2016; Opie et al., 2020) also used the intervention mapping approach (Kok et al., 2016) for intervention development, resulting in a description of BCTs.

4 Other techniques were also tested within single-component interventions, but among a lower number of arms and are therefore not described in detail here.

5 Note that behavioral outcomes differ in magnitude, as they may for example be cross-sectional (e.g. healthy purchases) or longitudinal (e.g. total time spent walking at baseline, 3-month and 9-month follow-up).

6 Note that also these behavioral outcomes vary in magnitude. Although all outcomes involved longitudinally measured abstinence, the duration of measured abstinence differed (e.g. four weeks, six months or two years).

## Open Scholarship

This article has earned the Center for Open Science badges for Open Data, Open Materials and Preregistered. The data and materials are openly accessible at https://osf.io/25eyg/.
