# Supplementary material for: Health behavior interventions among people with lower socio-economic position: a scoping review of behavior change techniques and effectiveness
Source: Health Psychol Behav Med. 2024 Jun 18;12(1):2365931. doi: 10.1080/21642850.2024.2365931 (PMC11188964; doi:10.1080/21642850.2024.2365931)
Supplement: Supplemental Material [file RHPB_A_2365931_SM4104.docx]

Health behavior interventions among people with lower socio-economic position: A scoping review of behavior change techniques and effectiveness – Supplementary file 3 References included papers

Abusabha, R., Namjoshi, D., & Klein, A. (2011). Increasing access and affordability of produce improves perceived consumption of vegetables in low-income seniors. *Journal of American Dietetic Assocation*, *111*(10), 1549-1555. https://doi.org/10.1016/j.jada.2011.07.003

Almabadi, E. S., Seymour, G. J., Akhter, R., Bauman, A., Cullinan, M. P., & Eberhard, J. (2021). Reduction of hsCRP levels following an Oral Health Education Program combined with routine dental treatment. *Journal of Dentistry*, *110*, Article 103686. https://doi.org/10.1016/j.jdent.2021.103686

Andrews, D. W. (2017). *A qualitative exploration of the impact of the Beach Community Wellness Program on nutrition educators and participants* (Publication No. 10254691) [Master’s thesis, California State University Long Beach]. ProQuest Dissertations Publishing. https://www.proquest.com/dissertations-theses/qualitative-exploration-impact-beach-community/docview/1877615127/se-2?accountid=12045

Anjali, R. (2013). *Self-regulation of saturated fat intake in blue-collar employees: A randomized intervention study* (Publication No. 3565069) [Doctoral dissertation, The University of Wisconsin – Milwaukee]. ProQuest Dissertations Publishing. https://www.proquest.com/docview/1411971964

Armitage, C. J., & Arden, M. A. (2010). A volitional help sheet to increase physical activity in people with low socioeconomic status: A randomised exploratory trial. *Psychology & Health*, *25*(10), 1129-1145. https://doi.org/10.1080/08870440903121638

Backman, D., Scruggs, V., Atiedu, A. A., Bowie, S., Bye, L., Dennis, A., Hall, M., Ossa, A., Wertlieb, S., & Foerster, S. B. (2011). Using a Toolbox of tailored educational lessons to improve fruit, vegetable, and physical activity behaviors among African American women in California. *Journal of Nutrition Education and Behavior*, *43*(4, Suppl. 2), S75-S85. https://doi.org/10.1016/j.jneb.2011.02.004

Bailey, D., & Kerlin, L. (2015). Can Health Trainers Make a Difference With Difficult-to-Engage Clients? A Multisite Case Study. *Health Promotion Practice*, *16*(5), 756-764. https://doi.org/10.1177/1524839915572802

Balcázar, H., Fernández-Gaxiola, A. C., Pérez-Lizaur, A. B., Peyron, R. A., & Ayala, C. (2015). Improving heart healthy lifestyles among participants in a Salud para su Corazón promotores model: The Mexican pilot study, 2009-2012. *Preventing Chronic Disease*, *12*, Article 140292. https://doi.org/10.5888/pcd12.140292

Ball, K., McNaughton, S. A., Le, H. N., Abbott, G., Stephens, L. D., & Crawford, D. A. (2016). ShopSmart 4 Health: Results of a randomized controlled trial of a behavioral intervention promoting fruit and vegetable consumption among socioeconomically disadvantaged women. *The American Journal of Clinical Nutrition*, *104*(2), 436-445. https://doi.org/10.3945/ajcn.116.133173

Baltaci, A. (2022). *Latino father-focused, healthy lifestyle intervention to improve adolescent energy balance-related behaviors* (Publication No. 28965258) [Doctoral dissertation, University of Minnesota]. ProQuest Dissertations Publishing. https://www.proquest.com/dissertations-theses/latino-father-focused-healthy-lifestyle/docview/2641840805/se-2?accountid=12045

Bhargava, A., & Hays, J. (2004). Behavioral variables and education are predictors of dietary change in the women's health trial: Feasibility Study in Minority Populations. *Preventive Medicine*, *38*(4), 442-451. https://doi.org/10.1016/j.ypmed.2003.11.014

Bird, C. L., & McClelland, J. W. (2017). Educating limited resource older adults for better choices to lower risk of food insecurity. *International Journal of Consumer Studies*, *41*(2), 225-233. https://doi.org/10.1111/ijcs.12333

Blom, E. E., Aadland, E., Solbraa, A. K., & Oldervoll, L. M. (2020). Healthy Life Centres: A 3-month behaviour change programme's impact on participants' physical activity levels, aerobic fitness and obesity: An observational study. *BMJ Open*, *10*(9), Article e035888. https://doi.org/10.1136/bmjopen-2019-035888

Bopp, M., Wilcox, S., Laken, M., Hooker, S. P., Parra-Medina, D., Saunders, R., Butler, K., Fallon, E. A., & McClorin, L. (2009). 8 Steps to fitness: A faith-based, behavior change physical activity intervention for African Americans. *Journal of Physical Activity and Health*, *6*(5), 568-577. https://doi.org/https://dx.doi.org/10.1123/jpah.6.5.568

Brett, E. I., Chavarria, J., Liu, M., Hedeker, D., & King, A. C. (2021). Effects of a brief motivational smoking intervention in non-treatment seeking disadvantaged Black smokers. *Journal of Consulting and Clinical Psychology*, *89*(4), 241-250. https://doi.org/10.1037/ccp0000629

Brown, C. C., Tilford, J. M., & Bird, T. M. (2018). Improved health and insurance status among cigarette smokers after Medicaid expansion, 2011-2016. *Public Health Reports*, *133*(3), 294-302. https://doi.org/10.1177/0033354918763169

Brownson, R. C., Baker, E. A., Boyd, R. L., Caito, N. M., Duggan, K., Housemann, R. A., Kreuter, M. W., Mitchell, T., Motton, F., Pulley, C., Schmid, T. L., & Walton, D. (2004). A community-based approach to promoting walking in rural areas. *American Journal of Preventive Medicine*, *27*(1), 28-34. https://doi.org/10.1016/j.amepre.2004.03.015

Bullock, K. (2022). *Changes in healthy eating and physical activity behaviors of adult participants in create better health's education (SNAP-Ed) program* (Publication No. 29327787) [Master’s thesis, Utah State University]. ProQuest Dissertations Publishing. https://www.proquest.com/dissertations-theses/changes-healthy-eating-physical-activity/docview/2702500959/se-2?accountid=12045

Cahill, E., Schmidt, S. R., Henry, T. L., Kumar, G., Berney, S., Bussey-Jones, J., & Girard, A. W. (2020). Qualitative research study on addressing barriers to healthy diet among low-income individuals at an urban, safety-net hospital. *BMJ Nutrition, Prevention & Health*, *3*(2), 383-386. https://doi.org/10.1136/bmjnph-2020-000064

Castagna, J. (2004). *The impact of nutrition education videos in changing the nutrition behavior of low-income men and women in drug rehabilitation* (Publication No. 3157940) [Doctoral dissertation, Dowling College]. ProQuest Dissertations Publishing. https://www.proquest.com/dissertations-theses/impact-nutrition-education-videos-changing/docview/305041847/se-2?accountid=12045

Chaput, S., Mercille, G., Drouin, L., & Kestens, Y. (2018). Promoting access to fresh fruits and vegetables through a local market intervention at a subway station. *Public Health Nutrition*, *21*(17), 3258-3270. https://doi.org/10.1017/s1368980018001921

Cohen, A. J., Richardson, C. R., Heisler, M., Sen, A., Murphy, E. C., Hesterman, O. B., Davis, M. M., & Zick, S. M. (2017). Increasing Use of a Healthy Food Incentive: A Waiting Room Intervention Among Low-Income Patients. *American Journal of Preventive Medicine*, *52*(2), 154-162. https://doi.org/10.1016/j.amepre.2016.11.008

Coupe, N., Cotterill, S., & Peters, S. (2022). Enhancing community weight loss groups in a low socioeconomic status area: Application of the COM-B model and Behaviour Change Wheel. *Health Expect*, *25*(5), 2043-2055. https://doi.org/10.1111/hex.13325

Cullen, K. W., Thompson, D. I., Scott, A. R., Lara-Smalling, A., Watson, K. B., & Konzelmann, K. (2010). The impact of goal attainment on behavioral and mediating variables among low income women participating in an Expanded Food and Nutrition Education Program intervention study. *Appetite*, *55*(2), 305-310. https://doi.org/10.1016/j.appet.2010.06.017

Cummins, S., Flint, E., & Matthews, S. A. (2014). New neighborhood grocery store increased awareness of food access but did not alter dietary habits or obesity. *Health Affairs*, *33*(2), 283-291. https://doi.org/10.1377/hlthaff.2013.0512

Darker, C. D., Burke, E., Castello, S., O'Sullivan, K., O'Connell, N., Vance, J., Reynolds, C., Buggy, A., Dougall, N., Loudon, K., Williams, P., Dobbie, F., Bauld, L., & Hayes, C. B. (2022). A process evaluation of 'We Can Quit': A community-based smoking cessation intervention targeting women from areas of socio-disadvantage in Ireland. *BMC Public Health*, *22*(1), Article 1528. https://doi.org/10.1186/s12889-022-13957-5

Davidson, K. A. (2017). *Behavioral economics, nutrition education, and access to markets: Experimental evidence and a theoretical framework for improving dietary diversity* (Publication No. 13847420) [Doctoral dissertation, University of Florida]. ProQuest Dissertations Publishing. https://www.proquest.com/dissertations-theses/behavioral-economics-nutrition-education-access/docview/2185961104/se-2?accountid=12045

De Menezes, M. C., Mingoti, S. A., Cardoso, C. S., Mendonca, R. D., & Lopes, A. C. S. (2015). Intervention based on Transtheoretical Model promotes anthropometric and nutritional improvements: A randomized controlled trial. *Eating behaviors*, *17*, 37‐44. https://doi.org/10.1016/j.eatbeh.2014.12.007

DeBiasse, M. A. (2016). *The theory of planned behavior and implementation intentions to describe and improve fruit and vegetable intake in women of low socioeconomic status* (Publication No. 10191278) [Doctoral dissertation, Boston University]. ProQuest Dissertations Publishing. https://www.proquest.com/dissertations-theses/theory-planned-behavior-implementation-intentions/docview/1853453702/se-2?accountid=12045

Dickin, K. L., Hill, T. F., & Dollahite, J. S. (2014). Practice-based evidence of effectiveness in an integrated nutrition and parenting education intervention for low-income parents. *Journal of the Academy of Nutrition and Dietetics*, *114*(6), 945-950. https://doi.org/10.1016/j.jand.2013.09.029

Dollahite, J. S., Pijai, E. I., Scott-Pierce, M., Parker, C., & Trochim, W. (2014). A randomized controlled trial of a community-based nutrition education program for low-income parents. *Journal of Nutrition Education and Behavior*, *46*(2), 102-109. https://doi.org/10.1016/j.jneb.2013.09.004

Dressel, A., Schneider, R., DeNomie, M., Kusch, J., Welch, W., Sosa, M., Yeldell, S., Maida, T., Wineberg, J., Holt, K., & Bernstein, R. (2018). Assessing health promotion interventions: Limitations of traditional research methods in community-based studies. *Health Promotion Practice*, *19*(4), 573-580. https://doi.org/10.1177/1524839917725489

Einterz, F. E. (2017). *Skim milk tastes like water: Assessing the impact of a nutrition program for women in the San Joaquin Valley* (Publication No. 10605329) [Master’s thesis, University of California, Davis]. ProQuest Dissertations Publishing. https://www.proquest.com/dissertations-theses/skim-milk-tastes-like-water-assessing-impact/docview/1970496306/se-2?accountid=12045

Elbel, B., Kersh, R., Brescoll, V. L., & Dixon, L. B. (2009). Calorie labeling and food choices: A first look at the effects on low-income people in New York City. *Health Affairs*, *28*(Suppl. 1), w1110-w1121. https://doi.org/10.1377/hlthaff.28.6.w1110

Elbel, B., Mijanovich, T., Kiszko, K., Abrams, C., Cantor, J., & Dixon, L. B. (2017). The introduction of a supermarket via tax-credits in a low-income area. *American Journal of Health Promotion*, *31*(1), 59-66. https://doi.org/10.4278/ajhp.150217-QUAN-733

Essman, M., Taillie, L. S., Frank, T., Ng, S. W., Popkin, B. M., & Swart, E. C. (2021). Taxed and untaxed beverage intake by South African young adults after a national sugar-sweetened beverage tax: A before-and-after study. *PLoS Medicine*, *18*(5), Article e1003574. https://doi.org/10.1371/journal.pmed.1003574

Evoy, K. E., Ford, K. H., Nduaguba, S., Taylor, A., & Thomas, L. (2019). A pilot study assessing the addition of a Quit and Win program to pharmacist-led intensive smoking cessation therapy in a predominantly underserved, minority population. *Tobacco Prevention & Cessation*, *5*, Article 44. https://doi.org/10.18332/tpc/113356

Fernandez, A., Lozano, A., Lee, T. K., Messiah, S. E., & Prado, G. (2022). A healthy lifestyle intervention for Hispanic families: Moderating effects of education, income, nativity. *Journal of Nutrition Education and Behavior*, *54*(2), 125-134. https://doi.org/10.1016/j.jneb.2021.05.001

Fernando Prieto Peres, M., Prieto Peres Mercante, J., & Belitardo de Oliveira, A. (2019). Non-pharmacological treatment for primary headaches prevention and lifestyle changes in a low-income community of Brazil: A randomized clinical trial. *Headache*, *59*(1), 86-96. https://doi.org/10.1111/head.13457

Forde, H., & Solomon-Moore, E. (2019). A qualitative study to understand the potential efficacy of an information-based sugar reduction intervention among low socioeconomic individuals in the UK. *International Journal of Environmental Research and Public Health*, *16*(3). https://doi.org/10.3390/ijerph16030413

Gallegos, D., Do, H., To, Q. G., Vo, B., Goris, J., & Alraman, H. (2021). The effectiveness of living well multicultural-lifestyle management program among ethnic populations in Queensland, Australia. *Health Promotion Journal of Australia*, *32*(1), 84-95. https://doi.org/10.1002/hpja.329

Gallois, K. M., Buck, C., Dreas, J. A., Hassel, H., & Zeeb, H. (2013). Evaluation of an intervention using a self-regulatory counselling aid: Pre- and post- intervention results of the OPTIMAHL 60plus study. *International Journal of Public Health*, *58*(3), 449-458. https://doi.org/10.1007/s00038-012-0420-7

Gardner, B., Cane, J., Rumsey, N., & Michie, S. (2012). Behaviour change among overweight and socially disadvantaged adults: A longitudinal study of the NHS Health Trainer Service. *Psychology & Health*, *27*(10), 1178-1193. https://doi.org/10.1080/08870446.2011.652112

Giles, E. L., Becker, F., Ternent, L., Sniehotta, F. F., McColl, E., & Adams, J. (2016). Acceptability of financial incentives for health behaviours: A discrete choice experiment. *PLoS One*, *11*(6), Article e0157403. https://doi.org/10.1371/journal.pone.0157403

Gillespie, R., DeWitt, E., Slone, S., Cardarelli, K., & Gustafson, A. (2022). The impact of a grocery store closure in one rural highly obese Appalachian community on shopping behavior and dietary intake. *International Journal of Environmental Research and Public Health*, *19*(6), Article 3506. https://doi.org/10.3390/ijerph19063506

Gittelsohn, J., Song, H. J., Suratkar, S., Kumar, M. B., Henry, E. G., Sharma, S., Mattingly, M., & Anliker, J. A. (2010). An urban food store intervention positively affects food-related psychosocial variables and food behaviors. *Health Education & Behavior*, *37*(3), 390-402. https://doi.org/10.1177/1090198109343886

Gray, K. E., Hoerster, K. D., Taylor, L., Krieger, J., & Nelson, K. M. (2021). Improvements in physical activity and some dietary behaviors in a community health worker-led diabetes self-management intervention for adults with low incomes: Results from a randomized controlled trial. *Translational Behavioral Medicine*, *11*(12), 2144-2154. https://doi.org/10.1093/tbm/ibab113

Gressier, M., Sassi, F., & Frost, G. (2021). Contribution of reformulation, product renewal, and changes in consumer behavior to the reduction of salt intakes in the UK population between 2008/2009 and 2016/2017. *The American Journal of Clinical Nutrition*, *114*(3), 1092-1099. https://doi.org/10.1093/ajcn/nqab130

Griffin, T., Sun, Y., Sidhu, M., Adab, P., Burgess, A., Collins, C., Daley, A., Entwistle, A., Frew, E., Hardy, P., Hurley, K., Jones, L., McGee, E., Pallan, M., Young, M., Morgan, P., & Jolly, K. (2019). Healthy Dads, Healthy Kids UK, a weight management programme for fathers: Feasibility RCT. *BMJ Open*, *9*(12), Article e033534. https://doi.org/10.1136/bmjopen-2019-033534

Gutierrez, J., Devia, C., Weiss, L., Chantarat, T., Ruddock, C., Linnell, J., Golub, M., Godfrey, L., Rosen, R., & Calman, N. (2014). Health, community, and spirituality: Evaluation of a multicultural faith-based diabetes prevention program. *The Diabetes Educator*, *40*(2), 214-222. https://doi.org/10.1177/0145721714521872

Halpern, S. D., French, B., Small, D. S., Saulsgiver, K., Harhay, M. O., Audrain-McGovern, J., Loewenstein, G., Asch, D. A., & Volpp, K. G. (2016). Heterogeneity in the effects of reward- and deposit-based financial incentives on smoking cessation. *American Journal of Respiratory and Critical Care Medicine*, *194*(8), 981-988. https://doi.org/10.1164/rccm.201601-0108OC

Hamilton, D. (2016). *Increasing farmers market access among low-income shoppers in Washington State: Understanding the role of peer-to-peer programs* (Publication No. 10248907) [Master's thesis, University of Washington]. ProQuest Dissertations Publishing. https://www.proquest.com/dissertations-theses/increasing-farmers-market-access-among-low-income/docview/1872599139/se-2?accountid=12045

Han, M. A. (2019). The price of tobacco and its effects on smoking behaviors in Korea: The 2015 Korea Community Health Survey. *Preventive Medicine*, *120*, 71-77. https://doi.org/10.1016/j.ypmed.2019.01.010

Hand, R. K., Birnbaum, A. S., Carter, B. J., Medrow, L., Stern, E., & Brown, K. (2014). The RD parent empowerment program creates measurable change in the behaviors of low-income families and children: An intervention description and evaluation. *Journal of the Academy of Nutrition and Dietetics*, *114*(12), 1923-1931. https://doi.org/10.1016/j.jand.2014.08.014

Hankonen, N., Absetz, P., Haukkala, A., & Uutela, A. (2009). Socioeconomic status and psychosocial mechanisms of lifestyle change in a type 2 diabetes prevention trial. *Annals of Behavioral Medicine*, *38*(2), 160-165. https://doi.org/10.1007/s12160-009-9144-1

Hardcastle, S., Blake, N., & Hagger, M. S. (2012). The effectiveness of a motivational interviewing primary-care based intervention on physical activity and predictors of change in a disadvantaged community. *Journal of Behavioral Medicine*, *35*(3), 318-333. https://doi.org/10.1007/s10865-012-9417-1

Hayashi, T., Farrell, M. A., Chaput, L. A., Rocha, D. A., & Hernandez, M. (2010). Lifestyle intervention, behavioral changes, and improvement in cardiovascular risk profiles in the California WISEWOMAN project. *Journal of Women’s Health*, *19*(6), 1129-1138. https://doi.org/10.1089/jwh.2009.1631

Hays, L. M., Hoen, H. M., Slaven, J. E., Finch, E. A., Marrero, D. G., Saha, C., & Ackermann, R. T. (2016). Effects of a community-based lifestyle intervention on change in physical activity among economically disadvantaged adults with prediabetes. *American Journal of Health Education*, *47*(5), 266‐278. https://doi.org/10.1080/19325037.2016.1203839

Hersey, J. C., Cates, S. C., Blitstein, J. L., Kosa, K. M., Santiago Rivera, O. J., Contreras, D. A., Long, V. A., Singh, A., & Berman, D. A. (2015). Eat Smart, Live Strong intervention increases fruit and vegetable consumption among low-income older adults. *Journal of Nutrition in Gerontology and Geriatrics*, *34*(1), 66-80. https://doi.org/10.1080/21551197.2015.1007199

Hiscock, R., Murray, S., Brose, L. S., McEwen, A., Bee, J. L., Dobbie, F., & Bauld, L. (2013). Behavioural therapy for smoking cessation: The effectiveness of different intervention types for disadvantaged and affluent smokers. *Addictive Behaviors*, *38*(11), 2787-2796. https://doi.org/10.1016/j.addbeh.2013.07.010

Hoenink, J. C., Mackenbach, J. D., Waterlander, W., Lakerveld, J., Van der Laan, N., & Beulens, J. W. J. (2020). The effects of nudging and pricing on healthy food purchasing behavior in a virtual supermarket setting: A randomized experiment. *International Journal of Behavioral Nutrition and Physical Activity*, *17*(1), Article 98. https://doi.org/10.1186/s12966-020-01005-7

Huf, S. W., Volpp, K. G., Asch, D. A., Bair, E., & Venkataramani, A. (2018). Association of Medicaid healthy behavior incentive programs with smoking cessation, weight loss, and annual preventive health visits. *JAMA Network Open*, *1*(8), Article e186185. https://doi.org/10.1001/jamanetworkopen.2018.6185

Huitink, M., Poelman, M. P., Van den Eynde, E., Seidell, J. C., & Dijkstra, S. C. (2020). Social norm nudges in shopping trolleys to promote vegetable purchases: A quasi-experimental study in a supermarket in a deprived urban area in the Netherlands. *Appetite*, *151*, Article 104655. https://doi.org/10.1016/j.appet.2020.104655

Jemmott, J. B., 3rd, Stephens-Shields, A., O'Leary, A., Jemmott, L. S., Teitelman, A., Ngwane, Z., & Mtose, X. (2015). Mediation of effects of a theory-based behavioral intervention on self-reported physical activity in South African men. *Preventive Medicine*, *72*, 1-7. https://doi.org/10.1016/j.ypmed.2014.12.022

Jenum, A. K., Lorentzen, C. A., & Ommundsen, Y. (2009). Targeting physical activity in a low socioeconomic status population: Observations from the Norwegian 'Romsas in Motion' study. *British Journal of Sports Medicine*, *43*(1), 64-69. https://doi.org/10.1136/bjsm.2008.053637

Jeremias, E., Chatkin, J. M., Chatkin, G., Seibert, J., Martins, M., & Wagner, M. (2012). Smoking cessation in older adults. *International Journal of Tuberculosis and Lung Disease*, *16*(2), 273-278. https://doi.org/10.5588/ijtld.11.0312

Kamstrup-Larsen, N., Dalton, S. O., Grønbæk, M., Broholm-Jørgensen, M., Thomsen, J. L., Larsen, L. B., Johansen, C., & Tolstrup, J. (2019). The effectiveness of general practice-based health checks on health behaviour and incidence on non-communicable diseases in individuals with low socioeconomic position: A randomised controlled trial in Denmark. *BMJ Open*, *9*(9), Article e029180. https://doi.org/10.1136/bmjopen-2019-029180

Kannan, S., Ranjit, N., Ganguri, H. B., Lasichak, A., Sparks, A., Scherer, H., & Schulz, A. (2020). Sisters Together: Lessons learned from implementing a grocery store campaign in Michigan communities at risk for neural tube defects. *Journal of Health Care for the Poor and Underserved*, *31*(1), 301-324. https://doi.org/10.1353/hpu.2020.0024

Katenga-Kaunda, L. Z., Iversen, P. O., Kamudoni, P. R., Holmboe-Ottesen, G., & Fjeld, H. E. (2022). Food-based nutrition counselling and education intervention for improved diets of pregnant women in rural Malawi: A qualitative study of factors influencing dietary behaviour change. *Public Health Nutrition*, *25*(9), 2436-2447. https://doi.org/10.1017/s1368980022000593

Katz, K. S., Blake, S. M., Milligan, R. A., Sharps, P. W., White, D. B., Rodan, M. F., Rossi, M., & Murray, K. B. (2008). The design, implementation and acceptability of an integrated intervention to address multiple behavioral and psychosocial risk factors among pregnant African American women. *BMC Pregnancy Childbirth*, *8*, Article 22. https://doi.org/10.1186/1471-2393-8-22

Khare, M. M., Carpenter, R. A., Huber, R., Bates, N. J., Cursio, J. F., Balmer, P. W., Nolen, K. N., Hudson, H., Shippee, S. J., & Loo, R. K. (2012). Lifestyle intervention and cardiovascular risk reduction in the Illinois WISEWOMAN program. *Journal of Women's Health*, *21*(3), 294-301. https://doi.org/10.1089/jwh.2011.2926

Khare, M. M., Cursio, J. F., Locklin, C. A., Bates, N. J., & Loo, R. K. (2014). Lifestyle intervention and cardiovascular disease risk reduction in low-income Hispanic immigrant women participating in the Illinois WISEWOMAN program. *Journal of Community Health*, *39*(4), 737-746. https://doi.org/10.1007/s10900-014-9820-3

Kim, E. J., Nho, J. H., Kim, H. Y., & Park, S. K. (2021). The effects of lifestyle interventions on the health-promoting behavior, type D personality, cognitive function and body composition of low-income middle-aged Korean women. *International Journal of Environmental Research and Public Health*, *18*(11). https://doi.org/10.3390/ijerph18115637

Kim, S., & Cho, S. I. (2022). Smoking-related behaviour changes among Korean men after the 2015 tobacco price increase: Assessing the implications for the tobacco endgame using a reconstructed retrospective cohort study. *BMJ Open*, *12*(1), Article e051712. https://doi.org/10.1136/bmjopen-2021-051712

Klassen, A. C., Garrett-Mayer, E., Houts, P. S., Shankar, S., & Torio, C. M. (2008). The relationship of body size to participation and success in a fruits and vegetables intervention among low-income women. *Journal of Community Health*, *33*(2), 78-89. https://doi.org/10.1007/s10900-007-9072-6

Ko, I. S., Lee, T. H., Kim, G. S., Kang, S. W., & Kim, M. J. (2011). Effects of visiting nurses' individually tailored education for low-income adult diabetic patients in Korea. *Public Health Nursinging*, *28*(5), 429-437. https://doi.org/10.1111/j.1525-1446.2011.00941.x

Landais, L. L., Van Wijk, E. C., & Harting, J. (2021). Smoking cessation in lower socioeconomic groups: Adaptation and pilot test of a rolling group intervention. *BioMed Research International*, *2021*, Article 8830912. https://doi.org/10.1155/2021/8830912

Langenberg, P., Ballesteros, M., Feldman, R., Damron, D., Anliker, J., & Havas, S. (2000). Psychosocial factors and intervention-associated changes in those factors as correlates of change in fruit and vegetable consumption in the Maryland WIC 5 A Day Promotion Program. *Annals of Behavioral Medicine*, *22*(4), 307-315. https://doi.org/10.1007/bf02895667

López-Núñez, C., Secades-Villa, R., Peña-Suárez, E., Fernández-Artamendi, S., & Weidberg, S. (2017). Income levels and response to contingency management for smoking cessation. *Substance Use & Misuse*, *52*(7), 875-883. https://doi.org/10.1080/10826084.2016.1264973

Lucumí, D. I., Sarmiento, O. L., Forero, R., Gomez, L. F., & Espinosa, G. (2006). Community intervention to promote consumption of fruits and vegetables, smoke-free homes, and physical activity among home caregivers in Bogotá, Colombia. *Preventing Chronic Disease*, *3*(4), 1-13.

Majlesi, F., Foroshanie, A. R., Tal, A., & Nasrolahnejad, J. (2018). The effect of blended educational program on improving health promoting behaviors in patients with type 2 diabetes. *Journal of Research in Medical and Dental Science*, *6*(3), 332-338. https://doi.org/10.24896/jrmds.20186351

Mareno, N. (2014). An early-phase translation study of the ways to enhance children's activity and nutrition (We Can!) programme for low-income families. *Journal of Clinical Nursing*, *23*(11-12), 1760-1762. https://doi.org/10.1111/jocn.12210

Menne, H. L., Borato, L. M., Shelton, E. G., & Johnson, J. D. (2016). Promoting heart health and behavior change in a vulnerable older adult population. *Healthy Aging Research*, *5*, Article 11.

Mier, N., Tanguma, J., Millard, A. V., Villarreal, E. K., Alen, M., & Ory, M. G. (2011). A pilot walking program for Mexican-American women living in colonias at the border. *American Journal of Health Promotion*, *25*(3), 172-175. https://doi.org/10.4278/ajhp.090325-ARB-115

Minou, M. (2011). *Physical activity promotion of ethnic populations in deprived communities: From determinants to intervention* (Publication No. U568275) [Doctoral dissertation, Liverpool John Moores University (United Kingdom)]. ProQuest Dissertations Publishing. https://www.proquest.com/dissertations-theses/physical-activity-promotion-ethnic-populations/docview/1779233819/se-2?accountid=12045

Murray, S. M., Skavenski Van Wyk, S., Metz, K., Mulemba, S. M., Mwenge, M. M., Kane, J. C., Alto, M., Venturo-Conerly, K. E., Wasil, A. R., Fine, S. L., & Murray, L. K. (2021). A qualitative exploration of mechanisms of intimate partner violence reduction for Zambian couples receiving the Common Elements Treatment Approach (CETA) intervention. *Social Science Medicine*, *268*, Article 113458. https://doi.org/10.1016/j.socscimed.2020.113458

Ni Mhurchu, C., Eyles, H., Dixon, R., Matoe, L., Teevale, T., & Meagher-Lundberg, P. (2012). Economic incentives to promote healthier food purchases: Exploring acceptability and key factors for success. *Health Promotion International*, *27*(3), 331-341. https://doi.org/10.1093/heapro/dar042

Nieves, C. I., Dannefer, R., Zamula, A., Fonseca, A., Myers, C., Brown-Dudley, L., & Manyindo, N. (2022). A qualitative evaluation of a community-based nutrition and health promotion program. *Journal of Hunger & Environmental Nutrition*, *17*(3), 318-332. https://doi.org/10.1080/19320248.2021.1898514

Nour, K., Laforest, S., Gauvin, L., & Gignac, M. (2006). Behavior change following a self-management intervention for housebound older adults with arthritis: An experimental study. *International Journal of Behavioral Nutrition and Physical Activity*, *3*, Article 12. https://doi.org/10.1186/1479-5868-3-12

Ohly, H. (2018). *A realist investigation of the impact of 'healthy start' on the diets of low-income pregnant women in the UK* (Publication No. 13833084) [Doctoral dissertation, University of Central Lancashire (United Kingdom)]. ProQuest Dissertations Publishing. https://www.proquest.com/dissertations-theses/realist-investigation-impact-healthy-start-on/docview/2164569777/se-2?accountid=12045

Oli, N., Vaidya, A., Eiben, G., & Krettek, A. (2019). Effectiveness of health promotion regarding diet and physical activity among Nepalese mothers and their young children: The Heart-health Associated Research, Dissemination, and Intervention in the Community (HARDIC) trial. *Global Health Action*, *12*(1), Article 1670033. https://doi.org/10.1080/16549716.2019.1670033

Opie, R. S., McNaughton, S. A., Crawford, D., Abbott, G., & Ball, K. (2020). How and why does discretionary food consumption change when we promote fruit and vegetables? Results from the ShopSmart randomised controlled trial. *Public Health Nutrition*, *23*(1), 124-133. https://doi.org/10.1017/s1368980019002830

Otterbach, L., Mena, N. Z., Greene, G., Redding, C. A., De Groot, A., & Tovar, A. (2018). Community-based childhood obesity prevention intervention for parents improves health behaviors and food parenting practices among Hispanic, low-income parents. *BMC Obesity*, *5*, Article 11. https://doi.org/10.1186/s40608-018-0188-2

Panter, J., & Ogilvie, D. (2017). Can environmental improvement change the population distribution of walking? *Journal of Epidemiology and Community Health*, *71*(6), 528-535. https://doi.org/10.1136/jech-2016-208417

Parks, M. J., Kingsbury, J. H., Boyle, R. G., & Choi, K. (2017). Behavioral change in response to a statewide tobacco tax increase and differences across socioeconomic status. *Addictive Behaviors*, *73*, 209-215. https://doi.org/10.1016/j.addbeh.2017.05.019

Perignon, M., Dubois, C., Gazan, R., Maillot, M., Muller, L., Ruffieux, B., Gaigi, H., & Darmon, N. (2017). Co-construction and evaluation of a prevention program for improving the nutritional quality of food purchases at no additional cost in a socioeconomically disadvantaged population. *Current Developments in Nutrition*, *1*(10), Article e001107. https://doi.org/10.3945/cdn.117.001107

Perkins, S., Daley, A., Yerxa, K., & Therrien, M. (2020). The effectiveness of the Expanded Food and Nutrition Education Program (EFNEP) on diet quality as measured by the Healthy Eating Index. *American Journal of Lifestyle Medicine*, *14*(3), 316-325. https://doi.org/10.1177/1559827619872733

Perkins-Porras, L., Cappuccio, F. P., Rink, E., Hilton, S., McKay, C., & Steptoe, A. (2005). Does the effect of behavioral counseling on fruit and vegetable intake vary with stage of readiness to change? *Preventive Medicine*, *40*(3), 314-320. https://doi.org/10.1016/j.ypmed.2004.06.002

Petersen, Z., Nilsson, M., Steyn, K., & Emmelin, M. (2013). Identifying with a process of change: A qualitative assessment of the components included in a smoking cessation intervention at antenatal clinics in South Africa. *Midwifery*, *29*(7), 751-758. https://doi.org/10.1016/j.midw.2012.07.016

Pettigrew, S., Moore, S., Pratt, I. S., & Jongenelis, M. (2016). Evaluation outcomes of a long-running adult nutrition education programme. *Public Health Nutrition*, *19*(4), 743-752. https://doi.org/10.1017/s1368980015001536

Prins, R. G., Kamphuis, C. B. M., & Van Lenthe, F. J. (2019). The effects of small-scale physical and social environmental interventions on walking behaviour among Dutch older adults living in deprived neighbourhoods: Results from the quasi-experimental NEW.ROADS study. *International Journal of Behavioral Nutrition and Physical Activity*, *16*(1), Article 133. https://doi.org/10.1186/s12966-019-0863-9

Rash, C. J., Petry, N. M., & Alessi, S. M. (2018). A randomized trial of contingency management for smoking cessation in the homeless. *Psychology of Addictive Behaviors*, *32*(2), 141-148. https://doi.org/10.1037/adb0000350

Realmuto, L., Kamler, A., Weiss, L., Gary-Webb, T. L., Hodge, M. E., Pagán, J. A., & Walker, E. A. (2018). Power Up for Health: Participants' perspectives on an adaptation of the national diabetes prevention program to engage men. *American Journal of Men’s Health*, *12*(4), 981-988. https://doi.org/10.1177/1557988318758786

Resnick, B., Shaughnessy, M., Galik, E., Scheve, A., Fitten, R., Morrison, T., Michael, K., & Agness, C. (2009). Pilot testing of the PRAISEDD intervention among African American and low-income older adults. *Journal of Cardiovascular Nursing*, *24*(5), 352-361. https://doi.org/10.1097/JCN.0b013e3181ac0301

Rhoads-Baeza, M. E. (2008). *Assessing the knowledge, attitudes, and behaviors of pregnant Hispanic women: Developing an effective educational intervention for gestational diabetes* (Publication No. 3337895) [Doctoral dissertation, University of Illinois at Urbana-Champaign]. ProQuest Dissertations Publishing. https://www.proquest.com/docview/304603883?pq-origsite=gscholar&fromopenview=true

Ries, A. V., Blackman, L. T., Page, R. A., Gizlice, Z., Benedict, S., Barnes, K., Kelsey, K., & Carter-Edwards, L. (2014). Goal setting for health behavior change: Evidence from an obesity intervention for rural low-income women. *Rural and Remote Health*, *14*, Article 2682.

Rimmer, J. H., Braunschweig, C., Silverman, K., Riley, B., Creviston, T., & Nicola, T. (2000). Effects of a short-term health promotion intervention for a predominantly African-American group of stroke survivors. *American Journal of Preventive Medicine*, *18*(4), 332-338. https://doi.org/10.1016/S0749-3797(00)00129-X

Rimmer, J. H., Silverman, K., Braunschweig, C., Quinn, L., & Liu, Y. (2002). Feasibility of a health promotion intervention for a group of predominantly African American women with type 2 diabetes. *The Diabetes Educator*, *28*(4), 571-580. https://doi.org/10.1177/014572170202800411

Ritten, A., Waldrop, J., & Kitson, J. (2016). Fit living in progress: Fighting lifelong obesity patterns (FLIP-FLOP): A nurse practitioner delivered intervention. *Applied Nursing Research*, *30*, 119-124. https://doi.org/10.1016/j.apnr.2015.09.006

Rustad, C., & Smith, C. (2013). Nutrition knowledge and associated behavior changes in a holistic, short-term nutrition education intervention with low-income women. *Journal of Nutrition Education and Behavior*, *45*(6), 490-498. https://doi.org/10.1016/j.jneb.2013.06.009

Saito, Y., Tanaka, A., Tajima, T., Ito, T., Aihara, Y., Nakano, K., Kamada, M., Inoue, S., Miyachi, M., Lee, I. M., & Oguma, Y. (2021). A community-wide intervention to promote physical activity: A five-year quasi-experimental study. *Preventive Medicine*, *150*, Article 106708. https://doi.org/10.1016/j.ypmed.2021.106708

Sanz-Remacha, M., García-González, L., Sevil Serrano, J., & Aibar Solana, A. (2023). A qualitative evaluation of a community-based intervention on health-related behaviors in disadvantaged women. *Research Quarterly for Exercise and Sport*, *94*(1), 272-282. https://doi.org/10.1080/02701367.2021.1971149

Schlosser, A. V., Joshi, K., Smith, S., Thornton, A., Bolen, S. D., & Trapl, E. S. (2019). "The coupons and stuff just made it possible": Economic constraints and patient experiences of a produce prescription program. *Translational Behavioral Medicine*, *9*(5), 875-883. https://doi.org/10.1093/tbm/ibz086

Schulz, A. J., Israel, B. A., Mentz, G. B., Bernal, C., Caver, D., DeMajo, R., Diaz, G., Gamboa, C., Gaines, C., Hoston, B., Opperman, A., Reyes, A. G., Rowe, Z., Sand, S. L., & Woods, S. (2015). Effectiveness of a walking group intervention to promote physical activity and cardiovascular health in predominantly non-Hispanic black and Hispanic urban neighborhoods: Findings from the walk your heart to health intervention. *Health Education & Behavior*, *42*(3), 380-392. https://doi.org/10.1177/1090198114560015

Scott, S., Gupta, S., Menon, P., Raghunathan, K., Thai, G., Quisumbing, A., Prasad, V., Hegde, A., Choudhury, A., Khetan, M., Nichols, C., & Kumar, N. (2022). A quasi-experimental evaluation of a nutrition behavior change intervention delivered through women's self-help groups in rural India: Impacts on maternal and young child diets, anthropometry, and intermediate outcomes. *Current Developments in Nutrition*, *6*(6), Article nzac079. https://doi.org/10.1093/cdn/nzac079

Seguin-Fowler, R. A., Hanson, K. L., Pitts, S. B. J., Kolodinsky, J., Sitaker, M., Ammerman, A. S., Marshall, G. A., Belarmino, E. H., Garner, J. A., & Wang, W. W. (2021). Community supported agriculture plus nutrition education improves skills, self-efficacy, and eating behaviors among low-income caregivers but not their children: A randomized controlled trial. *International Journal of Behavioral Nutrition and Physical Activity*, *18*(1), Article 112. https://doi.org/10.1186/s12966-021-01168-x

Siero, F. W., Broer, J., Bemelmans, W. J., & Meyboom-De Jong, B. M. (2000). Impact of group nutrition education and surplus value of Prochaska-based stage-matched information on health-related cognitions and on Mediterranean nutrition behavior. *Health Education Research*, *15*(5), 635-647. https://doi.org/10.1093/her/15.5.635

Skalka, A. J. (2020). *Evaluation of the nutrition education for limited resource audiences in Iowa* (Publication No. 28093643) [Master’s thesis, Iowa State University]. ProQuest Dissertations Publishing. https://www.proquest.com/dissertations-theses/evaluation-nutrition-education-limited-resource/docview/2479729225/se-2?accountid=12045

Skinner, H. G. (2017). *Personal utility: Examining the effects of genomic risk knowledge on motivation toward diet and physical activity behavior changes* (Publication No. 10690876) [Doctoral dissertation, The University of North Carolina at Chapel Hill]. ProQuest Dissertations Publishing. https://www.proquest.com/dissertations-theses/personal-utility-examining-effects-genomic-risk/docview/2007241626/se-2?accountid=12045

Snyder, S. A. (2019). *Testing theories and measures for promoting dietary change* (Publication No. 27629437) [Master’s thesis, The University of Texas at Arlington]. ProQuest Dissertations Publishing. https://www.proquest.com/dissertations-theses/testing-theories-measures-promoting-dietary/docview/2314065412/se-2?accountid=12045

Soni, A., Beeken, R. J., McGowan, L., Lawson, V., Chadwick, P., & Croker, H. (2021). 'Shape-Up', a modified cognitive-behavioural community programme for weight management: Real-world evaluation as an approach for delivering public health goals. *Nutrients*, *13*(8), Article 2807. https://doi.org/10.3390/nu13082807

Sorensen, G., Barbeau, E., Stoddard, A. M., Hunt, M. K., Kaphingst, K., & Wallace, L. (2005). Promoting behavior change among working-class, multiethnic workers: Results of the healthy directions–small business study. *American Journal of Public Health*, *95*(8), 1389-1395. https://doi.org/10.2105/ajph.2004.038745

Sorensen, G., Stoddard, A. M., Dubowitz, T., Barbeau, E. M., Bigby, J., Emmons, K. M., Berkman, L. F., & Peterson, K. E. (2007). The influence of social context on changes in fruit and vegetable consumption: Results of the healthy directions studies. *American Journal of Public Health*, *97*(7), 1216-1227. https://doi.org/10.2105/ajph.2006.088120

Stead, M., MacKintosh, A. M., Findlay, A., Sparks, L., Anderson, A. S., Barton, K., & Eadie, D. (2017). Impact of a targeted direct marketing price promotion intervention (Buywell) on food-purchasing behaviour by low income consumers: A randomised controlled trial. *Journal of Human Nutrition and Dietetics*, *30*(4), 524-533. https://doi.org/10.1111/jhn.12441

Steinberg, M. L., Rosen, R. L., Versella, M. V., Borges, A., & Leyro, T. M. (2020). A pilot randomized clinical trial of brief interventions to encourage quit attempts in smokers from socioeconomic disadvantage. *Nicotine & Tobacco Research*, *22*(9), 1500-1508. https://doi.org/10.1093/ntr/ntaa047

Steptoe, A., Perkins-Porras, L., Hilton, S., Rink, E., & Cappuccio, F. P. (2004). Quality of life and self-rated health in relation to changes in fruit and vegetable intake and in plasma vitamins C and E in a randomised trial of behavioural and nutritional education counselling. *Britisch Journal of Nutrition*, *92*(1), 177-184. https://doi.org/10.1079/bjn20041177

Steptoe, A., Perkins-Porras, L., McKay, C., Rink, E., Hilton, S., & Cappuccio, F. P. (2003). Behavioural counselling to increase consumption of fruit and vegetables in low income adults: Randomised trial. *British Medical Journal*, *326*, Article 855. https://doi.org/10.1136/bmj.326.7394.855

Steptoe, A., Perkins-Porras, L., Rink, E., Hilton, S., & Cappuccio, F. P. (2004). Psychological and social predictors of changes in fruit and vegetable consumption over 12 months following behavioral and nutrition education counseling. *Health Psychology*, *23*(6), 574-581. https://doi.org/10.1037/0278-6133.23.6.574

Tabuchi, T., Fujiwara, T., & Shinozaki, T. (2017). Tobacco price increase and smoking behaviour changes in various subgroups: A nationwide longitudinal 7-year follow-up study among a middle-aged Japanese population. *Tobacco Control*, *26*(1), 69-77. https://doi.org/10.1136/tobaccocontrol-2015-052804

Taft, C. T., Franz, M. R., Cole, H. E., D'Avanzato, C., & Rothman, E. F. (2021). Examining strength at home for preventing intimate partner violence in civilians. *Journal of Family Psychology*, *35*(6), 857-862. https://doi.org/10.1037/fam0000732

Taylor, T., Serrano, E., Anderson, J., & Kendall, P. (2000). Knowledge, skills, and behavior improvements on peer educators and low-income Hispanic participants after a stage of change-based bilingual nutrition education program. *Journal of Community Health*, *25*(3), 241-262. https://doi.org/10.1023/a:1005160216289

Towey, M., Harrell, R., & Lee, B. (2011). Evaluation of "One Body, One Life": A community-based family intervention for the prevention of obesity in children. *Journal of Obesity*, *2011*, Article 619643. https://doi.org/10.1155/2011/619643

Townsend, C. (2005). *Outcomes of a nutrition and gardening education program: A qualitative study* (Publication No. 1432236) [Master’s thesis, Michigan State University]. ProQuest Dissertations Publishing. https://www.proquest.com/dissertations-theses/outcomes-nutrition-gardening-education-program/docview/305476303/se-2?accountid=12045

Trapl, E. S., Smith, S., Joshi, K., Osborne, A., Benko, M., Matos, A. T., & Bolen, S. (2018). Dietary impact of produce prescriptions for patients with hypertension. *Preventing Chronic Disease*, *15*, Article E138. https://doi.org/10.5888/pcd15.180301

Verheijden, M. W., Van Dommelen, P., Van Empelen, P., Crone, M. R., Werkman, A. M., & Van Kesteren, N. M. (2012). Changes in self-reported energy balance behaviours and body mass index during a mass media campaign. *Family Practice*, *29* (Suppl. 1), i75-i81. https://doi.org/10.1093/fampra/cmr133

Vieten, C., Laraia, B. A., Kristeller, J., Adler, N., Coleman-Phox, K., Bush, N. R., Wahbeh, H., Duncan, L. G., & Epel, E. (2018). The mindful moms training: Development of a mindfulness-based intervention to reduce stress and overeating during pregnancy. *BMC Pregnancy Childbirth*, *18*(1), Article 201. https://doi.org/10.1186/s12884-018-1757-6

Visram, S., Clarke, C., & White, M. (2014). Making and maintaining lifestyle changes with the support of a lay health advisor: Longitudinal qualitative study of health trainer services in northern England. *PLoS One*, *9*(5), Article e94749. https://doi.org/10.1371/journal.pone.0094749

Voigt, E. C., Mutter, E. R., & Oettingen, G. (2022). Effectiveness of a motivational smoking reduction strategy across socioeconomic status and stress levels. *Frontiers in Psychology*, *13*, Article 801028. https://doi.org/10.3389/fpsyg.2022.801028

Walker, E. A., Weiss, L., Gary-Webb, T. L., Realmuto, L., Kamler, A., Ravenell, J., Tejeda, C., Lukin, J., & Schechter, C. B. (2018). Power Up for Health: Pilot study outcomes of a diabetes prevention program for men from disadvantaged neighborhoods. *American Journal of Men’s Health*, *12*(4), 989-997. https://doi.org/10.1177/1557988318758787

Waller, M., Blomstrand, A., Högberg, T., Ariai, N., Thorn, J., Hange, D., & Björkelund, C. (2016). A primary care lifestyle programme suitable for socioeconomically vulnerable groups: An observational study. *Scandinavian Journal of Primary Health Care*, *34*(4), 352-359. https://doi.org/10.1080/02813432.2016.1248628

Wang, M. C., MacLeod, K. E., Steadman, C., Williams, L., Bowie, S. L., Herd, D., Luluquisen, M., & Woo, M. (2007). Is the opening of a neighborhood full-service grocery store followed by a change in the food behavior of residents?. *Journal of Hunger & Environmental Nutrition*, *2*(1), 3-18. https://doi.org/10.1080/19320240802077789

Webb Hooper, M., Antoni, M. H., Okuyemi, K., Dietz, N. A., & Resnicow, K. (2017). Randomized controlled trial of group-based culturally specific cognitive behavioral therapy among African American smokers. *Nicotine & Tobacco Research*, *19*(3), 333-341. https://doi.org/10.1093/ntr/ntw181

Weinstein, E., Galindo, R. J., Fried, M., Rucker, L., & Davis, N. J. (2014). Impact of a focused nutrition educational intervention coupled with improved access to fresh produce on purchasing behavior and consumption of fruits and vegetables in overweight patients with diabetes mellitus. *The Diabetes Educator*, *40*(1), 100-106. https://doi.org/10.1177/0145721713508823

Weiss, L., Quint, E., Leto, C., Vaughn, I., Redrovan, A., Fernandes, M., Lamourt, K., Edgar, C., & Reso, A. (2021). Evaluation of an integrated health promotion program for a low-income urban population: Findings and lessons learned. *Public Health Nursing*, *38*(4), 571-578. https://doi.org/10.1111/phn.12839

West, E. G., Lindberg, R., Ball, K., & McNaughton, S. A. (2020). The role of a food literacy intervention in promoting food security and food literacy: OzHarvest's NEST program. *Nutrients*, *12*(8). Article 2197. https://doi.org/10.3390/nu12082197

White, R. (2012). *The effectiveness of an incentivized program to increase daily fruit and vegetable dietary intake by low income, middle-aged women* (Publication No. 1512456) [Master’s thesis, Minnesota State University, Mankato]. ProQuest Dissertations Publishing. https://www.proquest.com/dissertations-theses/effectiveness-incentivized-program-increase-daily/docview/1022504455/se-2?accountid=12045

White, S. C., Agurto, I., & Araguas, N. (2006). Promoting healthy behaviors to prevent chronic disease in Panama and Trinidad & Tobago: Results of the women as agents of change project. *Journal of Community Health*, *31*(5), 413-429. https://doi.org/10.1007/s10900-006-9022-8

Whitehead, D., Bodenlos, J. S., Cowles, M. L., Jones, G. N., & Brantley, P. J. (2007). A stage-targeted physical activity intervention among a predominantly African-American low-income primary care population. *American Journal of Health Promotion*, *21*(3), 160-163. https://doi.org/10.4278/0890-1171-21.3.160

Williams, A. R., Wilson-Genderson, M., & Thomson, M. D. (2021). A cross-sectional analysis of associations between lifestyle advice and behavior changes in patients with hypertension or diabetes: NHANES 2015-2018. *Preventive Medicine*, *145*, Article 106426. https://doi.org/10.1016/j.ypmed.2021.106426

Woodward-Lopez, G., Kao, J., Kuo, E. S., Rauzon, S., Taylor, A. C., Goette, C., Collins, C., Gonzalez, E. P., Ronshausen, D. R., Boyle, K., Williamson, D., & Cheadle, A. (2018). Changes in consumer purchases in stores participating in an obesity prevention initiative. *American Journal of Preventive Medicine*, *54*(5, Suppl. 2), S160-S169. https://doi.org/https://dx.doi.org/10.1016/j.amepre.2017.12.002

Zwolinsky, S., McKenna, J., Pringle, A., Daly-Smith, A., Robertson, S., & White, A. (2013). Optimizing lifestyles for men regarded as ‘hard-to-reach’ through top-flight football/soccer clubs. *Health Education Research*, *28*(3), 405-413. https://doi.org/10.1093/her/cys108
