# Supplementary material for: Health behavior interventions among people with lower socio-economic position: a scoping review of behavior change techniques and effectiveness
Source: Health Psychol Behav Med. 2024 Jun 18;12(1):2365931. doi: 10.1080/21642850.2024.2365931 (PMC11188964; doi:10.1080/21642850.2024.2365931)
Supplement: Supplemental Material [file RHPB_A_2365931_SM4100.docx]

Health behavior interventions among people with lower socio-economic position: A scoping review of behavior change techniques and effectiveness – Supplementary file 1 Final search strategy all databases

**PubMed**

(("Economic Status"[mesh] OR "Educational Status"[mesh] OR "Employment"[mesh] OR "Unemployment"[mesh] OR "Income"[mesh] OR "Salaries and Fringe Benefits"[mesh] OR "Occupations"[mesh] OR "Poverty"[mesh] OR "Poverty Areas"[mesh] OR "Social Class"[mesh] OR "Social Conditions"[mesh] OR "Economic Status"[tiab] OR "Educational Status"[tiab] OR "Employment"[tiab] OR "Income"[tiab] OR "Occupation"[tiab] OR "Occupations"[tiab] OR "Poverty"[tiab] OR "Poverty Area"[tiab] OR "Poverty Areas"[tiab] OR "Salaries"[tiab] OR "Salary"[tiab] OR "Social Class"[tiab] OR "Social Classes"[tiab] OR "Social Condition"[tiab] OR "Social Conditions"[tiab] OR "Unemployment"[tiab] OR "socioeconomic position"[tiab] OR "socioeconomic positions"[tiab] OR "socioeconomic posit*"[tiab] OR "socio economic position"[tiab] OR "socio economic positions"[tiab] OR "socio economic posit*"[tiab] OR "social economic position"[tiab] OR "social economic posit*"[tiab] OR "socioeconomic status"[tiab] OR "socio economic status"[tiab] OR "social economic status"[tiab] OR "deprived urban area"[tiab] OR "deprived suburban area"[tiab] OR "deprived area"[tiab] OR "blue collar"[tiab] OR "blue-collar"[tiab] OR "deprive*"[tiab] OR "deprived"[tiab] OR "disadvantaged"[tiab] OR "education level*"[tiab] OR "educational level"[tiab] OR "employment*"[tiab] OR "income*"[tiab] OR "job"[tiab] OR "jobs"[tiab] OR "low education"[tiab] OR "low income"[tiab] OR "low-educat*"[tiab] OR "low-income"[tiab] OR "social class*"[tiab] OR "social disparity"[tiab] OR "social disparities"[tiab] OR "social disparit*"[tiab] OR "social inequalit*"[tiab] OR "social inequalities"[tiab] OR "social inequality"[tiab] OR "social inequit*"[tiab] OR "social inequities"[tiab] OR "social inequity"[tiab] OR "Health Inequities"[Mesh] OR "Health inequity"[tiab] OR "Health inequality"[tiab] OR "Health inequities"[tiab] OR "Health inequalities"[tiab] OR "Health inequit*"[tiab] OR "Health inequalit*"[tiab] OR "Healthcare Disparities"[Mesh] OR "Healthcare Disparities"[tiab] OR "Healthcare Disparity"[tiab] OR "Health care Disparities"[tiab] OR "Health care Disparity"[tiab] OR "social position"[tiab] OR "social standing"[tiab] OR "social status"[tiab] OR "social strata"[tiab] OR "socioeconomic"[tiab] OR "socio-economic"[tiab] OR "socio-economic"[tiab] OR "socioeconomic factor"[tiab] OR "socioeconomic factors"[tiab] OR "Socio-economic factors"[tiab] OR "Socio-economic factor"[tiab] OR "socio-economic status"[tiab] OR "socioeconomically"[tiab] OR "socio-economically"[tiab] OR "underprivileged"[tiab] OR "unemployed"[tiab] OR "working class"[tiab] OR "working-class "[tiab] OR "years of education"[tiab] OR "years of schooling"[tiab] OR "job status"[tiab] OR "occupational status"[tiab] OR "occupation status"[tiab]) AND ("Exercise"[Mesh] OR "Physical activity"[tiab] OR "Physical activities"[tiab] OR "Exercise"[tiab] OR "Exercises"[tiab] OR "Exercising"[tiab] OR "Exercis*"[tiab] OR "Strength training"[tiab] OR "Aerobic"[tiab] OR "Aerobics"[tiab] OR "Resistance training"[tiab] OR "Walking"[tiab] OR "Endurance Training"[tiab] OR "Exergaming"[tiab] OR "Gymnastics"[tiab] OR "Interval Training"[tiab] OR "Jogging"[tiab] OR "Nordic Walking"[tiab] OR "Physical Conditioning"[tiab] OR "Running"[tiab] OR "Stair Climbing"[tiab] OR "Swimming"[tiab] OR "Sitting"[tiab] OR "Sedentary Behavior"[Mesh] OR "Sedentary behaviour"[tiab] OR "Sedentary behavior"[tiab] OR "Activity"[tiab] OR "activities"[tiab] OR "Inactivity"[tiab] OR "inactivities"[tiab] OR "Inactivit*"[tiab] OR "Diet"[mesh] OR "diet"[tiab] OR "diets"[tiab] OR "diet*"[tiab] OR "nutrition"[tiab] OR "nutritional"[tiab] OR "nutrition*"[tiab] OR "Eating"[mesh] OR "eat"[tiab] OR "eating"[tiab] OR "consumption"[tiab] OR "food intake"[tiab] OR "food pattern"[tiab] OR "food habit"[tiab] OR "food patterns"[tiab] OR "food habits"[tiab] OR "food intake"[tiab] OR "Food"[mesh] OR "food"[tiab] OR "foods"[tiab] OR "food-related"[tiab] OR "vegetable"[tiab] OR "vegetables"[tiab] OR "fruit"[tiab] OR "fruits"[tiab] OR "wholegrain"[tiab] OR "wholegrains"[tiab] OR "legume*"[tiab] OR "nut"[tiab] OR "nuts"[tiab] OR "dairy"[tiab] OR "fish"[tiab] OR "tea"[tiab] OR "fat"[tiab] OR "fats"[tiab] OR "oil"[tiab] OR "oils"[tiab] OR "coffee"[tiab] OR "red meat"[tiab] OR "processed meat"[tiab] OR "Food and Beverages"[Mesh] OR "Beverages"[Mesh] OR "sweetened beverage*"[tiab] OR "juice*"[tiab] OR "Drinking Behavior"[mesh] OR "Drinking Behavior"[tiab] OR "Drinking Behaviour"[tiab] OR "Alcohol Drinking"[mesh] OR "Ethanol"[Mesh] OR "Ethanol"[tiab] OR "alcohol"[tiab] OR "Alcohol consumption"[tiab] OR "Alcohol drinking"[tiab] OR "alcohol use"[tiab] OR "Energy Intake"[Mesh] OR "Weight Gain"[Mesh] OR "Weight gain"[tiab] OR "gain weight"[tiab] OR "gaining weight"[tiab] OR "poor diet"[tiab] OR "poor diets"[tiab] OR "poor dietary"[tiab] OR "healthy eating"[tiab] OR "Smoking"[mesh] OR "Smoking"[tiab] OR "Smoking Cessation"[mesh] OR "Smoking Reduction"[mesh] OR "Tobacco Use Cessation"[mesh] OR "Smoking cessation"[tiab] OR "Tobacco"[mesh] OR "Tobacco Products"[mesh] OR "Tobacco use"[mesh] OR "Tobacco use"[tiab]) AND ("behavior change"[tiab] OR "behavior changes"[tiab] OR "behavior chang*"[tiab] OR "behavioral change"[tiab] OR "behavioral changes"[tiab] OR "behavioral chang*"[tiab] OR "behaviour change"[tiab] OR "behaviour changes"[tiab] OR "behaviour chang*"[tiab] OR "behavioural change"[tiab] OR "behavioural changes"[tiab] OR "behavioural chang*"[tiab] OR "behavior change interventions"[tiab] OR "behavior change intervention"[tiab] OR "behaviour change interventions"[tiab] OR "behaviour change intervention"[tiab] OR "behavioral change interventions"[tiab] OR "behavioral change intervention"[tiab] OR "behavioural change interventions"[tiab] OR "behavioural change intervention"[tiab] OR "behavior change techniques"[tiab] OR "behavior change technique"[tiab] OR "behaviour change techniques"[tiab] OR "behaviour change technique"[tiab] OR "behavioral change techniques"[tiab] OR "behavioral change technique"[tiab] OR "behavioural change techniques"[tiab] OR "behavioural change technique"[tiab] OR "Behavior Change"[tiab] OR "Behavior Changes"[tiab] OR "Behavior experiment"[tiab] OR "Behavior experiments"[tiab] OR "Behavior Intervention"[tiab] OR "Behavior Interventions"[tiab] OR "Behavior Modification"[tiab] OR "Behavior Modifications"[tiab] OR "Behavior Program"[tiab] OR "Behavior Programme"[tiab] OR "Behavior Programmes"[tiab] OR "Behavior Programs"[tiab] OR "Behavior Promotion"[tiab] OR "Behavior Promotions"[tiab] OR "Behavior Trial"[tiab] OR "Behavior Trials"[tiab] OR "Behavioral Change"[tiab] OR "Behavioral Changes"[tiab] OR "Behavioral experiment"[tiab] OR "Behavioral experiments"[tiab] OR "Behavioral Intervention"[tiab] OR "Behavioral Interventions"[tiab] OR "Behavioral Modification"[tiab] OR "Behavioral Modifications"[tiab] OR "Behavioral Program"[tiab] OR "Behavioral Programme"[tiab] OR "Behavioral Programmes"[tiab] OR "Behavioral Programs"[tiab] OR "Behavioral Promotion"[tiab] OR "Behavioral Promotions"[tiab] OR "Behavioral Trial"[tiab] OR "Behavioral Trials"[tiab] OR "Behaviors Change"[tiab] OR "Behaviors Changes"[tiab] OR "Behaviors experiment"[tiab] OR "Behaviors experiments"[tiab] OR "Behaviors Intervention"[tiab] OR "Behaviors Interventions"[tiab] OR "Behaviors Modification"[tiab] OR "Behaviors Modifications"[tiab] OR "Behaviors Program"[tiab] OR "Behaviors Programme"[tiab] OR "Behaviors Programmes"[tiab] OR "Behaviors Programs"[tiab] OR "Behaviors Promotion"[tiab] OR "Behaviors Promotions"[tiab] OR "Behaviors Trial"[tiab] OR "Behaviors Trials"[tiab] OR "Behaviour Change"[tiab] OR "Behaviour Changes"[tiab] OR "Behaviour experiment"[tiab] OR "Behaviour experiments"[tiab] OR "Behaviour Intervention"[tiab] OR "Behaviour Interventions"[tiab] OR "Behaviour Modification"[tiab] OR "Behaviour Modifications"[tiab] OR "Behaviour Program"[tiab] OR "Behaviour Programme"[tiab] OR "Behaviour Programmes"[tiab] OR "Behaviour Programs"[tiab] OR "Behaviour Promotion"[tiab] OR "Behaviour Promotions"[tiab] OR "Behaviour Trial"[tiab] OR "Behaviour Trials"[tiab] OR "Behavioural Change"[tiab] OR "Behavioural Changes"[tiab] OR "Behavioural experiment"[tiab] OR "Behavioural experiments"[tiab] OR "Behavioural Intervention"[tiab] OR "Behavioural Interventions"[tiab] OR "Behavioural Modification"[tiab] OR "Behavioural Modifications"[tiab] OR "Behavioural Program"[tiab] OR "Behavioural Programme"[tiab] OR "Behavioural Programmes"[tiab] OR "Behavioural Programs"[tiab] OR "Behavioural Promotion"[tiab] OR "Behavioural Promotions"[tiab] OR "Behavioural Trial"[tiab] OR "Behavioural Trials"[tiab] OR "Behaviours Change"[tiab] OR "Behaviours Changes"[tiab] OR "Behaviours experiment"[tiab] OR "Behaviours experiments"[tiab] OR "Behaviours Intervention"[tiab] OR "Behaviours Interventions"[tiab] OR "Behaviours Modification"[tiab] OR "Behaviours Modifications"[tiab] OR "Behaviours Program"[tiab] OR "Behaviours Programme"[tiab] OR "Behaviours Programmes"[tiab] OR "Behaviours Programs"[tiab] OR "Behaviours Promotion"[tiab] OR "Behaviours Promotions"[tiab] OR "Behaviours Trial"[tiab] OR "Behaviours Trials"[tiab] OR (("promotion"[ti] OR "promote"[ti] OR "promoting"[ti]) AND ("intervention"[ti] OR "interventions"[ti]))) NOT ("Telemedicine"[majr] OR "e-health"[ti] OR "m-health"[ti] OR "ehealth"[ti] OR "mhealth"[ti] OR "telehealth"[ti] OR "schoolbased"[ti] OR "school based"[ti] OR "School Health Services"[majr] OR "digital intervention"[ti] OR "digital interventions"[ti] OR "digital"[ti] OR "online"[ti] OR "internet"[ti] OR "smartphone"[ti] OR "smartphones"[ti] OR "phone"[ti] OR "phones"[ti]) AND ("2000/01/01"[PDAT] : "3000/12/31"[PDAT]) NOT (("Infant"[mesh] OR "infant"[ti] OR "infants"[ti] OR "Child"[mesh] OR "child"[ti] OR "children"[ti] OR "childhood"[ti] OR "girl"[ti] OR "girls"[ti] OR "girlhood"[ti] OR "boy"[ti] OR "boys"[ti] OR "boyhood"[ti] OR "Adolescent"[mesh] OR "adolescent"[ti] OR "adolescents"[ti] OR "adolescence"[ti]) NOT ("Adult"[mesh] OR "adult"[ti] OR "adults"[ti] OR "adulthood"[ti] OR "elderly"[ti])))

**Medline (OVID)**

((exp "Economic Status"/ OR exp "Educational Status"/ OR exp "Employment"/ OR exp "Unemployment"/ OR exp "Income"/ OR exp "Salaries and Fringe Benefits"/ OR exp "Occupations"/ OR exp "Poverty"/ OR exp "Poverty Areas"/ OR exp "Social Class"/ OR exp "Social Conditions"/ OR "Economic Status".ti,ab OR "Educational Status".ti,ab OR "Employment".ti,ab OR "Income".ti,ab OR "Occupation".ti,ab OR "Occupations".ti,ab OR "Poverty".ti,ab OR "Poverty Area".ti,ab OR "Poverty Areas".ti,ab OR "Salaries".ti,ab OR "Salary".ti,ab OR "Social Class".ti,ab OR "Social Classes".ti,ab OR "Social Condition".ti,ab OR "Social Conditions".ti,ab OR "Unemployment".ti,ab OR "socioeconomic position".ti,ab OR "socioeconomic positions".ti,ab OR "socioeconomic posit*".ti,ab OR "socio economic position".ti,ab OR "socio economic positions".ti,ab OR "socio economic posit*".ti,ab OR "social economic position".ti,ab OR "social economic posit*".ti,ab OR "socioeconomic status".ti,ab OR "socio economic status".ti,ab OR "social economic status".ti,ab OR "deprived urban area".ti,ab OR "deprived suburban area".ti,ab OR "deprived area".ti,ab OR "blue collar".ti,ab OR "blue-collar".ti,ab OR "deprive*".ti,ab OR "deprived".ti,ab OR "disadvantaged".ti,ab OR "education level*".ti,ab OR "educational level".ti,ab OR "employment*".ti,ab OR "income*".ti,ab OR "job".ti,ab OR "jobs".ti,ab OR "low education".ti,ab OR "low income".ti,ab OR "low-educat*".ti,ab OR "low-income".ti,ab OR "social class*".ti,ab OR "social disparity".ti,ab OR "social disparities".ti,ab OR "social disparit*".ti,ab OR "social inequalit*".ti,ab OR "social inequalities".ti,ab OR "social inequality".ti,ab OR "social inequit*".ti,ab OR "social inequities".ti,ab OR "social inequity".ti,ab OR exp "Health Inequities"/ OR "Health inequity".ti,ab OR "Health inequality".ti,ab OR "Health inequities".ti,ab OR "Health inequalities".ti,ab OR "Health inequit*".ti,ab OR "Health inequalit*".ti,ab OR exp "Healthcare Disparities"/ OR "Healthcare Disparities".ti,ab OR "Healthcare Disparity".ti,ab OR "Health care Disparities".ti,ab OR "Health care Disparity".ti,ab OR "social position".ti,ab OR "social standing".ti,ab OR "social status".ti,ab OR "social strata".ti,ab OR "socioeconomic".ti,ab OR "socio-economic".ti,ab OR "socio-economic".ti,ab OR "socioeconomic factor".ti,ab OR "socioeconomic factors".ti,ab OR "Socio-economic factors".ti,ab OR "Socio-economic factor".ti,ab OR "socio-economic status".ti,ab OR "socioeconomically".ti,ab OR "socio-economically".ti,ab OR "underprivileged".ti,ab OR "unemployed".ti,ab OR "working class".ti,ab OR "working-class ".ti,ab OR "years of education".ti,ab OR "years of schooling".ti,ab OR "job status".ti,ab OR "occupational status".ti,ab OR "occupation status".ti,ab) AND (exp "Exercise"/ OR "Physical activity".ti,ab OR "Physical activities".ti,ab OR "Exercise".ti,ab OR "Exercises".ti,ab OR "Exercising".ti,ab OR "Exercis*".ti,ab OR "Strength training".ti,ab OR "Aerobic".ti,ab OR "Aerobics".ti,ab OR "Resistance training".ti,ab OR "Walking".ti,ab OR "Endurance Training".ti,ab OR "Exergaming".ti,ab OR "Gymnastics".ti,ab OR "Interval Training".ti,ab OR "Jogging".ti,ab OR "Nordic Walking".ti,ab OR "Physical Conditioning".ti,ab OR "Running".ti,ab OR "Stair Climbing".ti,ab OR "Swimming".ti,ab OR "Sitting".ti,ab OR exp "Sedentary Behavior"/ OR "Sedentary behaviour".ti,ab OR "Sedentary behavior".ti,ab OR "Activity".ti,ab OR "activities".ti,ab OR "Inactivity".ti,ab OR "inactivities".ti,ab OR "Inactivit*".ti,ab OR exp "Diet"/ OR "diet".ti,ab OR "diets".ti,ab OR "diet*".ti,ab OR "nutrition".ti,ab OR "nutritional".ti,ab OR "nutrition*".ti,ab OR exp "Eating"/ OR "eat".ti,ab OR "eating".ti,ab OR "consumption".ti,ab OR "food intake".ti,ab OR "food pattern".ti,ab OR "food habit".ti,ab OR "food patterns".ti,ab OR "food habits".ti,ab OR "food intake".ti,ab OR exp "Food"/ OR "food".ti,ab OR "foods".ti,ab OR "food-related".ti,ab OR "vegetable".ti,ab OR "vegetables".ti,ab OR "fruit".ti,ab OR "fruits".ti,ab OR "wholegrain".ti,ab OR "wholegrains".ti,ab OR "legume*".ti,ab OR "nut".ti,ab OR "nuts".ti,ab OR "dairy".ti,ab OR "fish".ti,ab OR "tea".ti,ab OR "fat".ti,ab OR "fats".ti,ab OR "oil".ti,ab OR "oils".ti,ab OR "coffee".ti,ab OR "red meat".ti,ab OR "processed meat".ti,ab OR exp "Food and Beverages"/ OR exp "Beverages"/ OR "sweetened beverage*".ti,ab OR "juice*".ti,ab OR exp "Drinking Behavior"/ OR "Drinking Behavior".ti,ab OR "Drinking Behaviour".ti,ab OR exp "Alcohol Drinking"/ OR exp "Ethanol"/ OR "Ethanol".ti,ab OR "alcohol".ti,ab OR "Alcohol consumption".ti,ab OR "Alcohol drinking".ti,ab OR "alcohol use".ti,ab OR exp "Energy Intake"/ OR exp "Weight Gain"/ OR "Weight gain".ti,ab OR "gain weight".ti,ab OR "gaining weight".ti,ab OR "poor diet".ti,ab OR "poor diets".ti,ab OR "poor dietary".ti,ab OR "healthy eating".ti,ab OR exp "Smoking"/ OR "Smoking".ti,ab OR exp "Smoking Cessation"/ OR exp "Smoking Reduction"/ OR exp "Tobacco Use Cessation"/ OR "Smoking cessation".ti,ab OR exp "Tobacco"/ OR exp "Tobacco Products"/ OR exp "Tobacco use"/ OR "Tobacco use".ti,ab) AND ("behavior change".ti,ab OR "behavior changes".ti,ab OR "behavior chang*".ti,ab OR "behavioral change".ti,ab OR "behavioral changes".ti,ab OR "behavioral chang*".ti,ab OR "behaviour change".ti,ab OR "behaviour changes".ti,ab OR "behaviour chang*".ti,ab OR "behavioural change".ti,ab OR "behavioural changes".ti,ab OR "behavioural chang*".ti,ab OR "behavior change interventions".ti,ab OR "behavior change intervention".ti,ab OR "behaviour change interventions".ti,ab OR "behaviour change intervention".ti,ab OR "behavioral change interventions".ti,ab OR "behavioral change intervention".ti,ab OR "behavioural change interventions".ti,ab OR "behavioural change intervention".ti,ab OR "behavior change techniques".ti,ab OR "behavior change technique".ti,ab OR "behaviour change techniques".ti,ab OR "behaviour change technique".ti,ab OR "behavioral change techniques".ti,ab OR "behavioral change technique".ti,ab OR "behavioural change techniques".ti,ab OR "behavioural change technique".ti,ab OR "Behavior Change".ti,ab OR "Behavior Changes".ti,ab OR "Behavior experiment".ti,ab OR "Behavior experiments".ti,ab OR "Behavior Intervention".ti,ab OR "Behavior Interventions".ti,ab OR "Behavior Modification".ti,ab OR "Behavior Modifications".ti,ab OR "Behavior Program".ti,ab OR "Behavior Programme".ti,ab OR "Behavior Programmes".ti,ab OR "Behavior Programs".ti,ab OR "Behavior Promotion".ti,ab OR "Behavior Promotions".ti,ab OR "Behavior Trial".ti,ab OR "Behavior Trials".ti,ab OR "Behavioral Change".ti,ab OR "Behavioral Changes".ti,ab OR "Behavioral experiment".ti,ab OR "Behavioral experiments".ti,ab OR "Behavioral Intervention".ti,ab OR "Behavioral Interventions".ti,ab OR "Behavioral Modification".ti,ab OR "Behavioral Modifications".ti,ab OR "Behavioral Program".ti,ab OR "Behavioral Programme".ti,ab OR "Behavioral Programmes".ti,ab OR "Behavioral Programs".ti,ab OR "Behavioral Promotion".ti,ab OR "Behavioral Promotions".ti,ab OR "Behavioral Trial".ti,ab OR "Behavioral Trials".ti,ab OR "Behaviors Change".ti,ab OR "Behaviors Changes".ti,ab OR "Behaviors experiment".ti,ab OR "Behaviors experiments".ti,ab OR "Behaviors Intervention".ti,ab OR "Behaviors Interventions".ti,ab OR "Behaviors Modification".ti,ab OR "Behaviors Modifications".ti,ab OR "Behaviors Program".ti,ab OR "Behaviors Programme".ti,ab OR "Behaviors Programmes".ti,ab OR "Behaviors Programs".ti,ab OR "Behaviors Promotion".ti,ab OR "Behaviors Promotions".ti,ab OR "Behaviors Trial".ti,ab OR "Behaviors Trials".ti,ab OR "Behaviour Change".ti,ab OR "Behaviour Changes".ti,ab OR "Behaviour experiment".ti,ab OR "Behaviour experiments".ti,ab OR "Behaviour Intervention".ti,ab OR "Behaviour Interventions".ti,ab OR "Behaviour Modification".ti,ab OR "Behaviour Modifications".ti,ab OR "Behaviour Program".ti,ab OR "Behaviour Programme".ti,ab OR "Behaviour Programmes".ti,ab OR "Behaviour Programs".ti,ab OR "Behaviour Promotion".ti,ab OR "Behaviour Promotions".ti,ab OR "Behaviour Trial".ti,ab OR "Behaviour Trials".ti,ab OR "Behavioural Change".ti,ab OR "Behavioural Changes".ti,ab OR "Behavioural experiment".ti,ab OR "Behavioural experiments".ti,ab OR "Behavioural Intervention".ti,ab OR "Behavioural Interventions".ti,ab OR "Behavioural Modification".ti,ab OR "Behavioural Modifications".ti,ab OR "Behavioural Program".ti,ab OR "Behavioural Programme".ti,ab OR "Behavioural Programmes".ti,ab OR "Behavioural Programs".ti,ab OR "Behavioural Promotion".ti,ab OR "Behavioural Promotions".ti,ab OR "Behavioural Trial".ti,ab OR "Behavioural Trials".ti,ab OR "Behaviours Change".ti,ab OR "Behaviours Changes".ti,ab OR "Behaviours experiment".ti,ab OR "Behaviours experiments".ti,ab OR "Behaviours Intervention".ti,ab OR "Behaviours Interventions".ti,ab OR "Behaviours Modification".ti,ab OR "Behaviours Modifications".ti,ab OR "Behaviours Program".ti,ab OR "Behaviours Programme".ti,ab OR "Behaviours Programmes".ti,ab OR "Behaviours Programs".ti,ab OR "Behaviours Promotion".ti,ab OR "Behaviours Promotions".ti,ab OR "Behaviours Trial".ti,ab OR "Behaviours Trials".ti,ab OR (("promotion".ti OR "promote".ti OR "promoting".ti) AND ("intervention".ti OR "interventions".ti)) OR (("change" OR "changes" OR "chang*" OR "experiment" OR "experiments" OR "Intervention" OR "Interventions" OR "Modification" OR "Modifications" OR "Program" OR "Programme" OR "Programmes" OR "Programs" OR "Promotion" OR "Promotions" OR "Trial" OR "Trials" OR "influence" OR "influences" OR "influencing" OR "influenced" OR "influenc*") ADJ3 ("behavior" OR "behaviors" OR "behavioral" OR "behavior*" OR "behaviour" OR "behaviours" OR "behavioural" OR "behaviour*") ADJ3 ("Exercise" OR "Physical activity" OR "Physical activities" OR "Exercise" OR "Exercises" OR "Exercising" OR "Exercis*" OR "Strength training" OR "Aerobic" OR "Aerobics" OR "Resistance training" OR "Walking" OR "Endurance Training" OR "Exergaming" OR "Gymnastics" OR "Interval Training" OR "Jogging" OR "Nordic Walking" OR "Physical Conditioning" OR "Running" OR "Stair Climbing" OR "Swimming" OR "Sitting" OR "Sedentary Behavior" OR "Sedentary behaviour" OR "Sedentary behavior" OR "Activity" OR "activities" OR "Inactivity" OR "inactivities" OR "Inactivit*" OR "Diet" OR "diet" OR "diets" OR "diet*" OR "nutrition" OR "nutritional" OR "nutrition*" OR "Eating" OR "eat" OR "eating" OR "consumption" OR "food intake" OR "food pattern" OR "food habit" OR "food patterns" OR "food habits" OR "food intake" OR "Food" OR "food" OR "foods" OR "food-related" OR "vegetable" OR "vegetables" OR "fruit" OR "fruits" OR "wholegrain" OR "wholegrains" OR "legume*" OR "nut" OR "nuts" OR "dairy" OR "fish" OR "tea" OR "fat" OR "fats" OR "oil" OR "oils" OR "coffee" OR "red meat" OR "processed meat" OR "Food and Beverages" OR "Beverages" OR "sweetened beverage*" OR "juice*" OR "Drinking Behavior" OR "Drinking Behavior" OR "Drinking Behaviour" OR "Alcohol Drinking" OR "Ethanol" OR "Ethanol" OR "alcohol" OR "Alcohol consumption" OR "Alcohol drinking" OR "alcohol use" OR "Energy Intake" OR "Weight Gain" OR "Weight gain" OR "gain weight" OR "gaining weight" OR "poor diet" OR "poor diets" OR "poor dietary" OR "healthy eating" OR "Smoking" OR "Smoking" OR "Smoking Cessation" OR "Smoking Reduction" OR "Tobacco Use Cessation" OR "Smoking cessation" OR "Tobacco" OR "Tobacco Products" OR "Tobacco use" OR "Tobacco use")).ti,ab) NOT (exp *"Telemedicine"/ OR "e-health".ti OR "m-health".ti OR "ehealth".ti OR "mhealth".ti OR "telehealth".ti OR "schoolbased".ti OR "school based".ti OR exp "School Health Services"/ OR "digital intervention".ti OR "digital interventions".ti OR "digital".ti OR "online".ti OR "internet".ti OR "smartphone".ti OR "smartphones".ti OR "phone".ti OR "phones".ti) AND 2000:2023.(sa_year) NOT ((exp "Infant"/ OR "infant".ti OR "infants".ti OR exp "Child"/ OR "child".ti OR "children".ti OR "childhood".ti OR "girl".ti OR "girls".ti OR "girlhood".ti OR "boy".ti OR "boys".ti OR "boyhood".ti OR exp "Adolescent"/ OR "adolescent".ti OR "adolescents".ti OR "adolescence".ti) NOT (exp "Adult"/ OR "adult".ti OR "adults".ti OR "adulthood".ti OR "elderly".ti)))

**Embase (OVID)**

((exp *"social status"/ OR "Economic Status"/ OR exp *"Educational Status"/ OR exp *"Employment"/ OR exp *"Unemployment"/ OR exp *"Income"/ OR exp *"Salaries and Fringe Benefits"/ OR exp *"Occupations"/ OR exp *"Poverty"/ OR exp *"Poverty Areas"/ OR exp *"Social Class"/ OR exp *"Social Conditions"/ OR "Economic Status".ti,ab OR "Educational Status".ti,ab OR "Employment".ti,ab OR "Income".ti,ab OR "Occupation".ti,ab OR "Occupations".ti,ab OR "Poverty".ti,ab OR "Poverty Area".ti,ab OR "Poverty Areas".ti,ab OR "Salaries".ti,ab OR "Salary".ti,ab OR "Social Class".ti,ab OR "Social Classes".ti,ab OR "Social Condition".ti,ab OR "Social Conditions".ti,ab OR "Unemployment".ti,ab OR "socioeconomic position".ti,ab OR "socioeconomic positions".ti,ab OR "socioeconomic posit*".ti,ab OR "socio economic position".ti,ab OR "socio economic positions".ti,ab OR "socio economic posit*".ti,ab OR "social economic position".ti,ab OR "social economic posit*".ti,ab OR "socioeconomic status".ti,ab OR "socio economic status".ti,ab OR "social economic status".ti,ab OR "deprived urban area".ti,ab OR "deprived suburban area".ti,ab OR "deprived area".ti,ab OR "blue collar".ti,ab OR "blue-collar".ti,ab OR "deprive*".ti,ab OR "deprived".ti,ab OR "disadvantaged".ti,ab OR "education level*".ti,ab OR "educational level".ti,ab OR "employment*".ti,ab OR "income*".ti,ab OR "job".ti,ab OR "jobs".ti,ab OR "low education".ti,ab OR "low income".ti,ab OR "low-educat*".ti,ab OR "low-income".ti,ab OR "social class*".ti,ab OR "social disparity".ti,ab OR "social disparities".ti,ab OR "social disparit*".ti,ab OR "social inequalit*".ti,ab OR "social inequalities".ti,ab OR "social inequality".ti,ab OR "social inequit*".ti,ab OR "social inequities".ti,ab OR "social inequity".ti,ab OR exp *"Health Disparity"/ OR "Health inequity".ti,ab OR "Health inequality".ti,ab OR "Health inequities".ti,ab OR "Health inequalities".ti,ab OR "Health inequit*".ti,ab OR "Health inequalit*".ti,ab OR exp *"Health care Disparities"/ OR "Healthcare Disparities".ti,ab OR "Healthcare Disparity".ti,ab OR "Health care Disparities".ti,ab OR "Health care Disparity".ti,ab OR "social position".ti,ab OR "social standing".ti,ab OR "social status".ti,ab OR "social strata".ti,ab OR "socioeconomic".ti,ab OR "socio-economic".ti,ab OR "socio-economic".ti,ab OR "socioeconomic factor".ti,ab OR "socioeconomic factors".ti,ab OR "Socio-economic factors".ti,ab OR "Socio-economic factor".ti,ab OR "socio-economic status".ti,ab OR "socioeconomically".ti,ab OR "socio-economically".ti,ab OR "underprivileged".ti,ab OR "unemployed".ti,ab OR "working class".ti,ab OR "working-class ".ti,ab OR "years of education".ti,ab OR "years of schooling".ti,ab OR "job status".ti,ab OR "occupational status".ti,ab OR "occupation status".ti,ab) AND (exp *"Exercise"/ OR "Physical activity".ti,ab OR "Physical activities".ti,ab OR "Exercise".ti,ab OR "Exercises".ti,ab OR "Exercising".ti,ab OR "Exercis*".ti,ab OR "Strength training".ti,ab OR "Aerobic".ti,ab OR "Aerobics".ti,ab OR "Resistance training".ti,ab OR "Walking".ti,ab OR "Endurance Training".ti,ab OR "Exergaming".ti,ab OR "Gymnastics".ti,ab OR "Interval Training".ti,ab OR "Jogging".ti,ab OR "Nordic Walking".ti,ab OR "Physical Conditioning".ti,ab OR "Running".ti,ab OR "Stair Climbing".ti,ab OR "Swimming".ti,ab OR "Sitting".ti,ab OR exp *"Sedentary Lifestyle"/ OR "Sedentary behaviour".ti,ab OR "Sedentary behavior".ti,ab OR "Activity".ti,ab OR "activities".ti,ab OR "Inactivity".ti,ab OR "inactivities".ti,ab OR "Inactivit*".ti,ab OR exp *"Diet"/ OR "diet".ti,ab OR "diets".ti,ab OR "diet*".ti,ab OR "nutrition".ti,ab OR "nutritional".ti,ab OR "nutrition*".ti,ab OR exp *"Eating"/ OR "eat".ti,ab OR "eating".ti,ab OR "consumption".ti,ab OR "food intake".ti,ab OR "food pattern".ti,ab OR "food habit".ti,ab OR "food patterns".ti,ab OR "food habits".ti,ab OR "food intake".ti,ab OR exp *"Food"/ OR "food".ti,ab OR "foods".ti,ab OR "food-related".ti,ab OR "vegetable".ti,ab OR "vegetables".ti,ab OR "fruit".ti,ab OR "fruits".ti,ab OR "wholegrain".ti,ab OR "wholegrains".ti,ab OR "legume*".ti,ab OR "nut".ti,ab OR "nuts".ti,ab OR "dairy".ti,ab OR "fish".ti,ab OR "tea".ti,ab OR "fat".ti,ab OR "fats".ti,ab OR "oil".ti,ab OR "oils".ti,ab OR "coffee".ti,ab OR "red meat".ti,ab OR "processed meat".ti,ab OR exp *"Beverage"/ OR "sweetened beverage*".ti,ab OR "juice*".ti,ab OR exp *"Drinking Behavior"/ OR "Drinking Behavior".ti,ab OR "Drinking Behaviour".ti,ab OR exp *"Alcohol"/ OR "Ethanol".ti,ab OR "alcohol".ti,ab OR "Alcohol consumption".ti,ab OR "Alcohol drinking".ti,ab OR "alcohol use".ti,ab OR exp *"Caloric Intake"/ OR exp *"Body Weight Gain"/ OR "Weight gain".ti,ab OR "gain weight".ti,ab OR "gaining weight".ti,ab OR "poor diet".ti,ab OR "poor diets".ti,ab OR "poor dietary".ti,ab OR "healthy eating".ti,ab OR exp *"Smoking"/ OR "Smoking".ti,ab OR exp *"Smoking Cessation"/ OR exp *"Smoking Reduction"/ OR exp *"Tobacco Use"/ OR "Smoking cessation".ti,ab OR exp *"Tobacco"/ OR "Tobacco use".ti,ab) AND (exp *"behavior change"/ OR exp *"behavior modification"/ OR "behavior change".ti,ab OR "behavior changes".ti,ab OR "behavior chang*".ti,ab OR "behavioral change".ti,ab OR "behavioral changes".ti,ab OR "behavioral chang*".ti,ab OR "behaviour change".ti,ab OR "behaviour changes".ti,ab OR "behaviour chang*".ti,ab OR "behavioural change".ti,ab OR "behavioural changes".ti,ab OR "behavioural chang*".ti,ab OR "behavior change interventions".ti,ab OR "behavior change intervention".ti,ab OR "behaviour change interventions".ti,ab OR "behaviour change intervention".ti,ab OR "behavioral change interventions".ti,ab OR "behavioral change intervention".ti,ab OR "behavioural change interventions".ti,ab OR "behavioural change intervention".ti,ab OR "behavior change techniques".ti,ab OR "behavior change technique".ti,ab OR "behaviour change techniques".ti,ab OR "behaviour change technique".ti,ab OR "behavioral change techniques".ti,ab OR "behavioral change technique".ti,ab OR "behavioural change techniques".ti,ab OR "behavioural change technique".ti,ab OR "Behavior Change".ti,ab OR "Behavior Changes".ti,ab OR "Behavior experiment".ti,ab OR "Behavior experiments".ti,ab OR "Behavior Intervention".ti,ab OR "Behavior Interventions".ti,ab OR "Behavior Modification".ti,ab OR "Behavior Modifications".ti,ab OR "Behavior Program".ti,ab OR "Behavior Programme".ti,ab OR "Behavior Programmes".ti,ab OR "Behavior Programs".ti,ab OR "Behavior Promotion".ti,ab OR "Behavior Promotions".ti,ab OR "Behavior Trial".ti,ab OR "Behavior Trials".ti,ab OR "Behavioral Change".ti,ab OR "Behavioral Changes".ti,ab OR "Behavioral experiment".ti,ab OR "Behavioral experiments".ti,ab OR "Behavioral Intervention".ti,ab OR "Behavioral Interventions".ti,ab OR "Behavioral Modification".ti,ab OR "Behavioral Modifications".ti,ab OR "Behavioral Program".ti,ab OR "Behavioral Programme".ti,ab OR "Behavioral Programmes".ti,ab OR "Behavioral Programs".ti,ab OR "Behavioral Promotion".ti,ab OR "Behavioral Promotions".ti,ab OR "Behavioral Trial".ti,ab OR "Behavioral Trials".ti,ab OR "Behaviors Change".ti,ab OR "Behaviors Changes".ti,ab OR "Behaviors experiment".ti,ab OR "Behaviors experiments".ti,ab OR "Behaviors Intervention".ti,ab OR "Behaviors Interventions".ti,ab OR "Behaviors Modification".ti,ab OR "Behaviors Modifications".ti,ab OR "Behaviors Program".ti,ab OR "Behaviors Programme".ti,ab OR "Behaviors Programmes".ti,ab OR "Behaviors Programs".ti,ab OR "Behaviors Promotion".ti,ab OR "Behaviors Promotions".ti,ab OR "Behaviors Trial".ti,ab OR "Behaviors Trials".ti,ab OR "Behaviour Change".ti,ab OR "Behaviour Changes".ti,ab OR "Behaviour experiment".ti,ab OR "Behaviour experiments".ti,ab OR "Behaviour Intervention".ti,ab OR "Behaviour Interventions".ti,ab OR "Behaviour Modification".ti,ab OR "Behaviour Modifications".ti,ab OR "Behaviour Program".ti,ab OR "Behaviour Programme".ti,ab OR "Behaviour Programmes".ti,ab OR "Behaviour Programs".ti,ab OR "Behaviour Promotion".ti,ab OR "Behaviour Promotions".ti,ab OR "Behaviour Trial".ti,ab OR "Behaviour Trials".ti,ab OR "Behavioural Change".ti,ab OR "Behavioural Changes".ti,ab OR "Behavioural experiment".ti,ab OR "Behavioural experiments".ti,ab OR "Behavioural Intervention".ti,ab OR "Behavioural Interventions".ti,ab OR "Behavioural Modification".ti,ab OR "Behavioural Modifications".ti,ab OR "Behavioural Program".ti,ab OR "Behavioural Programme".ti,ab OR "Behavioural Programmes".ti,ab OR "Behavioural Programs".ti,ab OR "Behavioural Promotion".ti,ab OR "Behavioural Promotions".ti,ab OR "Behavioural Trial".ti,ab OR "Behavioural Trials".ti,ab OR "Behaviours Change".ti,ab OR "Behaviours Changes".ti,ab OR "Behaviours experiment".ti,ab OR "Behaviours experiments".ti,ab OR "Behaviours Intervention".ti,ab OR "Behaviours Interventions".ti,ab OR "Behaviours Modification".ti,ab OR "Behaviours Modifications".ti,ab OR "Behaviours Program".ti,ab OR "Behaviours Programme".ti,ab OR "Behaviours Programmes".ti,ab OR "Behaviours Programs".ti,ab OR "Behaviours Promotion".ti,ab OR "Behaviours Promotions".ti,ab OR "Behaviours Trial".ti,ab OR "Behaviours Trials".ti,ab OR (("promotion" OR "promote" OR "promoting") AND ("intervention" OR "interventions")).ti OR (("change" OR "changes" OR "chang*" OR "experiment" OR "experiments" OR "Intervention" OR "Interventions" OR "Modification" OR "Modifications" OR "Program" OR "Programme" OR "Programmes" OR "Programs" OR "Promotion" OR "Promotions" OR "Trial" OR "Trials" OR "influence" OR "influences" OR "influencing" OR "influenced" OR "influenc*") ADJ3 ("behavior" OR "behaviors" OR "behavioral" OR "behavior*" OR "behaviour" OR "behaviours" OR "behavioural" OR "behaviour*") ADJ3 ("Exercise" OR "Physical activity" OR "Physical activities" OR "Exercise" OR "Exercises" OR "Exercising" OR "Exercis*" OR "Strength training" OR "Aerobic" OR "Aerobics" OR "Resistance training" OR "Walking" OR "Endurance Training" OR "Exergaming" OR "Gymnastics" OR "Interval Training" OR "Jogging" OR "Nordic Walking" OR "Physical Conditioning" OR "Running" OR "Stair Climbing" OR "Swimming" OR "Sitting" OR "Sedentary Behavior" OR "Sedentary behaviour" OR "Sedentary behavior" OR "Activity" OR "activities" OR "Inactivity" OR "inactivities" OR "Inactivit*" OR "Diet" OR "diet" OR "diets" OR "diet*" OR "nutrition" OR "nutritional" OR "nutrition*" OR "Eating" OR "eat" OR "eating" OR "consumption" OR "food intake" OR "food pattern" OR "food habit" OR "food patterns" OR "food habits" OR "food intake" OR "Food" OR "food" OR "foods" OR "food-related" OR "vegetable" OR "vegetables" OR "fruit" OR "fruits" OR "wholegrain" OR "wholegrains" OR "legume*" OR "nut" OR "nuts" OR "dairy" OR "fish" OR "tea" OR "fat" OR "fats" OR "oil" OR "oils" OR "coffee" OR "red meat" OR "processed meat" OR "Food and Beverages" OR "Beverages" OR "sweetened beverage*" OR "juice*" OR "Drinking Behavior" OR "Drinking Behavior" OR "Drinking Behaviour" OR "Alcohol Drinking" OR "Ethanol" OR "Ethanol" OR "alcohol" OR "Alcohol consumption" OR "Alcohol drinking" OR "alcohol use" OR "Energy Intake" OR "Weight Gain" OR "Weight gain" OR "gain weight" OR "gaining weight" OR "poor diet" OR "poor diets" OR "poor dietary" OR "healthy eating" OR "Smoking" OR "Smoking" OR "Smoking Cessation" OR "Smoking Reduction" OR "Tobacco Use Cessation" OR "Smoking cessation" OR "Tobacco" OR "Tobacco Products" OR "Tobacco use" OR "Tobacco use")).ti,ab) NOT (exp *"Telemedicine"/ OR "e-health".ti OR "m-health".ti OR "ehealth".ti OR "mhealth".ti OR "telehealth".ti OR "schoolbased".ti OR "school based".ti OR exp *"School Health Services"/ OR "digital intervention".ti OR "digital interventions".ti OR "digital".ti OR "online".ti OR "internet".ti OR "smartphone".ti OR "smartphones".ti OR "phone".ti OR "phones".ti) AND 2000:2023.(sa_year) NOT ((exp *"Infant"/ OR "infant".ti OR "infants".ti OR exp *"Child"/ OR "child".ti OR "children".ti OR "childhood".ti OR "girl".ti OR "girls".ti OR "girlhood".ti OR "boy".ti OR "boys".ti OR "boyhood".ti OR exp *"Adolescent"/ OR "adolescent".ti OR "adolescents".ti OR "adolescence".ti) NOT (exp *"Adult"/ OR "adult".ti OR "adults".ti OR "adulthood".ti OR "elderly".ti)))

**Emcare (OVID)**

((exp *"social status"/ OR "Economic Status"/ OR exp *"Educational Status"/ OR exp *"Employment"/ OR exp *"Unemployment"/ OR exp *"Income"/ OR exp *"Salaries and Fringe Benefits"/ OR exp *"Occupations"/ OR exp *"Poverty"/ OR exp *"Poverty Areas"/ OR exp *"Social Class"/ OR exp *"Social Conditions"/ OR "Economic Status".ti,ab OR "Educational Status".ti,ab OR "Employment".ti,ab OR "Income".ti,ab OR "Occupation".ti,ab OR "Occupations".ti,ab OR "Poverty".ti,ab OR "Poverty Area".ti,ab OR "Poverty Areas".ti,ab OR "Salaries".ti,ab OR "Salary".ti,ab OR "Social Class".ti,ab OR "Social Classes".ti,ab OR "Social Condition".ti,ab OR "Social Conditions".ti,ab OR "Unemployment".ti,ab OR "socioeconomic position".ti,ab OR "socioeconomic positions".ti,ab OR "socioeconomic posit*".ti,ab OR "socio economic position".ti,ab OR "socio economic positions".ti,ab OR "socio economic posit*".ti,ab OR "social economic position".ti,ab OR "social economic posit*".ti,ab OR "socioeconomic status".ti,ab OR "socio economic status".ti,ab OR "social economic status".ti,ab OR "deprived urban area".ti,ab OR "deprived suburban area".ti,ab OR "deprived area".ti,ab OR "blue collar".ti,ab OR "blue-collar".ti,ab OR "deprive*".ti,ab OR "deprived".ti,ab OR "disadvantaged".ti,ab OR "education level*".ti,ab OR "educational level".ti,ab OR "employment*".ti,ab OR "income*".ti,ab OR "job".ti,ab OR "jobs".ti,ab OR "low education".ti,ab OR "low income".ti,ab OR "low-educat*".ti,ab OR "low-income".ti,ab OR "social class*".ti,ab OR "social disparity".ti,ab OR "social disparities".ti,ab OR "social disparit*".ti,ab OR "social inequalit*".ti,ab OR "social inequalities".ti,ab OR "social inequality".ti,ab OR "social inequit*".ti,ab OR "social inequities".ti,ab OR "social inequity".ti,ab OR exp *"Health Disparity"/ OR "Health inequity".ti,ab OR "Health inequality".ti,ab OR "Health inequities".ti,ab OR "Health inequalities".ti,ab OR "Health inequit*".ti,ab OR "Health inequalit*".ti,ab OR exp *"Health care Disparities"/ OR "Healthcare Disparities".ti,ab OR "Healthcare Disparity".ti,ab OR "Health care Disparities".ti,ab OR "Health care Disparity".ti,ab OR "social position".ti,ab OR "social standing".ti,ab OR "social status".ti,ab OR "social strata".ti,ab OR "socioeconomic".ti,ab OR "socio-economic".ti,ab OR "socio-economic".ti,ab OR "socioeconomic factor".ti,ab OR "socioeconomic factors".ti,ab OR "Socio-economic factors".ti,ab OR "Socio-economic factor".ti,ab OR "socio-economic status".ti,ab OR "socioeconomically".ti,ab OR "socio-economically".ti,ab OR "underprivileged".ti,ab OR "unemployed".ti,ab OR "working class".ti,ab OR "working-class ".ti,ab OR "years of education".ti,ab OR "years of schooling".ti,ab OR "job status".ti,ab OR "occupational status".ti,ab OR "occupation status".ti,ab) AND (exp *"Exercise"/ OR "Physical activity".ti,ab OR "Physical activities".ti,ab OR "Exercise".ti,ab OR "Exercises".ti,ab OR "Exercising".ti,ab OR "Exercis*".ti,ab OR "Strength training".ti,ab OR "Aerobic".ti,ab OR "Aerobics".ti,ab OR "Resistance training".ti,ab OR "Walking".ti,ab OR "Endurance Training".ti,ab OR "Exergaming".ti,ab OR "Gymnastics".ti,ab OR "Interval Training".ti,ab OR "Jogging".ti,ab OR "Nordic Walking".ti,ab OR "Physical Conditioning".ti,ab OR "Running".ti,ab OR "Stair Climbing".ti,ab OR "Swimming".ti,ab OR "Sitting".ti,ab OR exp *"Sedentary Lifestyle"/ OR "Sedentary behaviour".ti,ab OR "Sedentary behavior".ti,ab OR "Activity".ti,ab OR "activities".ti,ab OR "Inactivity".ti,ab OR "inactivities".ti,ab OR "Inactivit*".ti,ab OR exp *"Diet"/ OR "diet".ti,ab OR "diets".ti,ab OR "diet*".ti,ab OR "nutrition".ti,ab OR "nutritional".ti,ab OR "nutrition*".ti,ab OR exp *"Eating"/ OR "eat".ti,ab OR "eating".ti,ab OR "consumption".ti,ab OR "food intake".ti,ab OR "food pattern".ti,ab OR "food habit".ti,ab OR "food patterns".ti,ab OR "food habits".ti,ab OR "food intake".ti,ab OR exp *"Food"/ OR "food".ti,ab OR "foods".ti,ab OR "food-related".ti,ab OR "vegetable".ti,ab OR "vegetables".ti,ab OR "fruit".ti,ab OR "fruits".ti,ab OR "wholegrain".ti,ab OR "wholegrains".ti,ab OR "legume*".ti,ab OR "nut".ti,ab OR "nuts".ti,ab OR "dairy".ti,ab OR "fish".ti,ab OR "tea".ti,ab OR "fat".ti,ab OR "fats".ti,ab OR "oil".ti,ab OR "oils".ti,ab OR "coffee".ti,ab OR "red meat".ti,ab OR "processed meat".ti,ab OR exp *"Beverage"/ OR "sweetened beverage*".ti,ab OR "juice*".ti,ab OR exp *"Drinking Behavior"/ OR "Drinking Behavior".ti,ab OR "Drinking Behaviour".ti,ab OR exp *"Alcohol"/ OR "Ethanol".ti,ab OR "alcohol".ti,ab OR "Alcohol consumption".ti,ab OR "Alcohol drinking".ti,ab OR "alcohol use".ti,ab OR exp *"Caloric Intake"/ OR exp *"Body Weight Gain"/ OR "Weight gain".ti,ab OR "gain weight".ti,ab OR "gaining weight".ti,ab OR "poor diet".ti,ab OR "poor diets".ti,ab OR "poor dietary".ti,ab OR "healthy eating".ti,ab OR exp *"Smoking"/ OR "Smoking".ti,ab OR exp *"Smoking Cessation"/ OR exp *"Smoking Reduction"/ OR exp *"Tobacco Use"/ OR "Smoking cessation".ti,ab OR exp *"Tobacco"/ OR "Tobacco use".ti,ab) AND (exp *"behavior change"/ OR exp *"behavior modification"/ OR "behavior change".ti,ab OR "behavior changes".ti,ab OR "behavior chang*".ti,ab OR "behavioral change".ti,ab OR "behavioral changes".ti,ab OR "behavioral chang*".ti,ab OR "behaviour change".ti,ab OR "behaviour changes".ti,ab OR "behaviour chang*".ti,ab OR "behavioural change".ti,ab OR "behavioural changes".ti,ab OR "behavioural chang*".ti,ab OR "behavior change interventions".ti,ab OR "behavior change intervention".ti,ab OR "behaviour change interventions".ti,ab OR "behaviour change intervention".ti,ab OR "behavioral change interventions".ti,ab OR "behavioral change intervention".ti,ab OR "behavioural change interventions".ti,ab OR "behavioural change intervention".ti,ab OR "behavior change techniques".ti,ab OR "behavior change technique".ti,ab OR "behaviour change techniques".ti,ab OR "behaviour change technique".ti,ab OR "behavioral change techniques".ti,ab OR "behavioral change technique".ti,ab OR "behavioural change techniques".ti,ab OR "behavioural change technique".ti,ab OR "Behavior Change".ti,ab OR "Behavior Changes".ti,ab OR "Behavior experiment".ti,ab OR "Behavior experiments".ti,ab OR "Behavior Intervention".ti,ab OR "Behavior Interventions".ti,ab OR "Behavior Modification".ti,ab OR "Behavior Modifications".ti,ab OR "Behavior Program".ti,ab OR "Behavior Programme".ti,ab OR "Behavior Programmes".ti,ab OR "Behavior Programs".ti,ab OR "Behavior Promotion".ti,ab OR "Behavior Promotions".ti,ab OR "Behavior Trial".ti,ab OR "Behavior Trials".ti,ab OR "Behavioral Change".ti,ab OR "Behavioral Changes".ti,ab OR "Behavioral experiment".ti,ab OR "Behavioral experiments".ti,ab OR "Behavioral Intervention".ti,ab OR "Behavioral Interventions".ti,ab OR "Behavioral Modification".ti,ab OR "Behavioral Modifications".ti,ab OR "Behavioral Program".ti,ab OR "Behavioral Programme".ti,ab OR "Behavioral Programmes".ti,ab OR "Behavioral Programs".ti,ab OR "Behavioral Promotion".ti,ab OR "Behavioral Promotions".ti,ab OR "Behavioral Trial".ti,ab OR "Behavioral Trials".ti,ab OR "Behaviors Change".ti,ab OR "Behaviors Changes".ti,ab OR "Behaviors experiment".ti,ab OR "Behaviors experiments".ti,ab OR "Behaviors Intervention".ti,ab OR "Behaviors Interventions".ti,ab OR "Behaviors Modification".ti,ab OR "Behaviors Modifications".ti,ab OR "Behaviors Program".ti,ab OR "Behaviors Programme".ti,ab OR "Behaviors Programmes".ti,ab OR "Behaviors Programs".ti,ab OR "Behaviors Promotion".ti,ab OR "Behaviors Promotions".ti,ab OR "Behaviors Trial".ti,ab OR "Behaviors Trials".ti,ab OR "Behaviour Change".ti,ab OR "Behaviour Changes".ti,ab OR "Behaviour experiment".ti,ab OR "Behaviour experiments".ti,ab OR "Behaviour Intervention".ti,ab OR "Behaviour Interventions".ti,ab OR "Behaviour Modification".ti,ab OR "Behaviour Modifications".ti,ab OR "Behaviour Program".ti,ab OR "Behaviour Programme".ti,ab OR "Behaviour Programmes".ti,ab OR "Behaviour Programs".ti,ab OR "Behaviour Promotion".ti,ab OR "Behaviour Promotions".ti,ab OR "Behaviour Trial".ti,ab OR "Behaviour Trials".ti,ab OR "Behavioural Change".ti,ab OR "Behavioural Changes".ti,ab OR "Behavioural experiment".ti,ab OR "Behavioural experiments".ti,ab OR "Behavioural Intervention".ti,ab OR "Behavioural Interventions".ti,ab OR "Behavioural Modification".ti,ab OR "Behavioural Modifications".ti,ab OR "Behavioural Program".ti,ab OR "Behavioural Programme".ti,ab OR "Behavioural Programmes".ti,ab OR "Behavioural Programs".ti,ab OR "Behavioural Promotion".ti,ab OR "Behavioural Promotions".ti,ab OR "Behavioural Trial".ti,ab OR "Behavioural Trials".ti,ab OR "Behaviours Change".ti,ab OR "Behaviours Changes".ti,ab OR "Behaviours experiment".ti,ab OR "Behaviours experiments".ti,ab OR "Behaviours Intervention".ti,ab OR "Behaviours Interventions".ti,ab OR "Behaviours Modification".ti,ab OR "Behaviours Modifications".ti,ab OR "Behaviours Program".ti,ab OR "Behaviours Programme".ti,ab OR "Behaviours Programmes".ti,ab OR "Behaviours Programs".ti,ab OR "Behaviours Promotion".ti,ab OR "Behaviours Promotions".ti,ab OR "Behaviours Trial".ti,ab OR "Behaviours Trials".ti,ab OR (("promotion" OR "promote" OR "promoting") AND ("intervention" OR "interventions")).ti OR (("change" OR "changes" OR "chang*" OR "experiment" OR "experiments" OR "Intervention" OR "Interventions" OR "Modification" OR "Modifications" OR "Program" OR "Programme" OR "Programmes" OR "Programs" OR "Promotion" OR "Promotions" OR "Trial" OR "Trials" OR "influence" OR "influences" OR "influencing" OR "influenced" OR "influenc*") ADJ3 ("behavior" OR "behaviors" OR "behavioral" OR "behavior*" OR "behaviour" OR "behaviours" OR "behavioural" OR "behaviour*") ADJ3 ("Exercise" OR "Physical activity" OR "Physical activities" OR "Exercise" OR "Exercises" OR "Exercising" OR "Exercis*" OR "Strength training" OR "Aerobic" OR "Aerobics" OR "Resistance training" OR "Walking" OR "Endurance Training" OR "Exergaming" OR "Gymnastics" OR "Interval Training" OR "Jogging" OR "Nordic Walking" OR "Physical Conditioning" OR "Running" OR "Stair Climbing" OR "Swimming" OR "Sitting" OR "Sedentary Behavior" OR "Sedentary behaviour" OR "Sedentary behavior" OR "Activity" OR "activities" OR "Inactivity" OR "inactivities" OR "Inactivit*" OR "Diet" OR "diet" OR "diets" OR "diet*" OR "nutrition" OR "nutritional" OR "nutrition*" OR "Eating" OR "eat" OR "eating" OR "consumption" OR "food intake" OR "food pattern" OR "food habit" OR "food patterns" OR "food habits" OR "food intake" OR "Food" OR "food" OR "foods" OR "food-related" OR "vegetable" OR "vegetables" OR "fruit" OR "fruits" OR "wholegrain" OR "wholegrains" OR "legume*" OR "nut" OR "nuts" OR "dairy" OR "fish" OR "tea" OR "fat" OR "fats" OR "oil" OR "oils" OR "coffee" OR "red meat" OR "processed meat" OR "Food and Beverages" OR "Beverages" OR "sweetened beverage*" OR "juice*" OR "Drinking Behavior" OR "Drinking Behavior" OR "Drinking Behaviour" OR "Alcohol Drinking" OR "Ethanol" OR "Ethanol" OR "alcohol" OR "Alcohol consumption" OR "Alcohol drinking" OR "alcohol use" OR "Energy Intake" OR "Weight Gain" OR "Weight gain" OR "gain weight" OR "gaining weight" OR "poor diet" OR "poor diets" OR "poor dietary" OR "healthy eating" OR "Smoking" OR "Smoking" OR "Smoking Cessation" OR "Smoking Reduction" OR "Tobacco Use Cessation" OR "Smoking cessation" OR "Tobacco" OR "Tobacco Products" OR "Tobacco use" OR "Tobacco use")).ti,ab) NOT (exp *"Telemedicine"/ OR "e-health".ti OR "m-health".ti OR "ehealth".ti OR "mhealth".ti OR "telehealth".ti OR "schoolbased".ti OR "school based".ti OR exp *"School Health Services"/ OR "digital intervention".ti OR "digital interventions".ti OR "digital".ti OR "online".ti OR "internet".ti OR "smartphone".ti OR "smartphones".ti OR "phone".ti OR "phones".ti) AND 2000:2023.(sa_year) NOT ((exp *"Infant"/ OR "infant".ti OR "infants".ti OR exp *"Child"/ OR "child".ti OR "children".ti OR "childhood".ti OR "girl".ti OR "girls".ti OR "girlhood".ti OR "boy".ti OR "boys".ti OR "boyhood".ti OR exp *"Adolescent"/ OR "adolescent".ti OR "adolescents".ti OR "adolescence".ti) NOT (exp *"Adult"/ OR "adult".ti OR "adults".ti OR "adulthood".ti OR "elderly".ti)))

**Web of Science (Core Collection)**

((TI=("Economic Status" OR "Educational Status" OR "Employment" OR "Unemployment" OR "Income" OR "Salaries and Fringe Benefits" OR "Occupations" OR "Poverty" OR "Poverty Areas" OR "Social Class" OR "Social Conditions" OR "Economic Status" OR "Educational Status" OR "Employment" OR "Income" OR "Occupation" OR "Occupations" OR "Poverty" OR "Poverty Area" OR "Poverty Areas" OR "Salaries" OR "Salary" OR "Social Class" OR "Social Classes" OR "Social Condition" OR "Social Conditions" OR "Unemployment" OR "socioeconomic position" OR "socioeconomic positions" OR "socioeconomic posit*" OR "socio economic position" OR "socio economic positions" OR "socio economic posit*" OR "social economic position" OR "social economic posit*" OR "socioeconomic status" OR "socio economic status" OR "social economic status" OR "deprived urban area" OR "deprived suburban area" OR "deprived area" OR "blue collar" OR "blue-collar" OR "deprive*" OR "deprived" OR "disadvantaged" OR "education level*" OR "educational level" OR "employment*" OR "income*" OR "job" OR "jobs" OR "low education" OR "low income" OR "low-educat*" OR "low-income" OR "social class*" OR "social disparity" OR "social disparities" OR "social disparit*" OR "social inequalit*" OR "social inequalities" OR "social inequality" OR "social inequit*" OR "social inequities" OR "social inequity" OR "health inequity" OR "health inequality" OR "health inequities" OR "health inequalities" OR "health inequit*" OR "health inequalit*" OR "Healthcare Disparity" OR "Healthcare Disparities" OR "Health care Disparity" OR "Health care Disparities" OR "social position" OR "social standing" OR "social status" OR "social strata" OR "socioeconomic" OR "socio-economic" OR "socio-economic" OR "socioeconomic factor" OR "socioeconomic factors" OR "Socio-economic factors" OR "Socio-economic factor" OR "socio-economic status" OR "socioeconomically" OR "socio-economically" OR "underprivileged" OR "unemployed" OR "working class" OR "working-class " OR "years of education" OR "years of schooling" OR "job status" OR "occupational status" OR "occupation status") OR AK=("Economic Status" OR "Educational Status" OR "Employment" OR "Unemployment" OR "Income" OR "Salaries and Fringe Benefits" OR "Occupations" OR "Poverty" OR "Poverty Areas" OR "Social Class" OR "Social Conditions" OR "Economic Status" OR "Educational Status" OR "Employment" OR "Income" OR "Occupation" OR "Occupations" OR "Poverty" OR "Poverty Area" OR "Poverty Areas" OR "Salaries" OR "Salary" OR "Social Class" OR "Social Classes" OR "Social Condition" OR "Social Conditions" OR "Unemployment" OR "socioeconomic position" OR "socioeconomic positions" OR "socioeconomic posit*" OR "socio economic position" OR "socio economic positions" OR "socio economic posit*" OR "social economic position" OR "social economic posit*" OR "socioeconomic status" OR "socio economic status" OR "social economic status" OR "deprived urban area" OR "deprived suburban area" OR "deprived area" OR "blue collar" OR "blue-collar" OR "deprive*" OR "deprived" OR "disadvantaged" OR "education level*" OR "educational level" OR "employment*" OR "income*" OR "job" OR "jobs" OR "low education" OR "low income" OR "low-educat*" OR "low-income" OR "social class*" OR "social disparity" OR "social disparities" OR "social disparit*" OR "social inequalit*" OR "social inequalities" OR "social inequality" OR "social inequit*" OR "social inequities" OR "social inequity" OR "health inequity" OR "health inequality" OR "health inequities" OR "health inequalities" OR "health inequit*" OR "health inequalit*" OR "Healthcare Disparity" OR "Healthcare Disparities" OR "Health care Disparity" OR "Health care Disparities" OR "social position" OR "social standing" OR "social status" OR "social strata" OR "socioeconomic" OR "socio-economic" OR "socio-economic" OR "socioeconomic factor" OR "socioeconomic factors" OR "Socio-economic factors" OR "Socio-economic factor" OR "socio-economic status" OR "socioeconomically" OR "socio-economically" OR "underprivileged" OR "unemployed" OR "working class" OR "working-class " OR "years of education" OR "years of schooling" OR "job status" OR "occupational status" OR "occupation status") OR AB=("Economic Status" OR "Educational Status" OR "Employment" OR "Unemployment" OR "Income" OR "Salaries and Fringe Benefits" OR "Occupations" OR "Poverty" OR "Poverty Areas" OR "Social Class" OR "Social Conditions" OR "Economic Status" OR "Educational Status" OR "Employment" OR "Income" OR "Occupation" OR "Occupations" OR "Poverty" OR "Poverty Area" OR "Poverty Areas" OR "Salaries" OR "Salary" OR "Social Class" OR "Social Classes" OR "Social Condition" OR "Social Conditions" OR "Unemployment" OR "socioeconomic position" OR "socioeconomic positions" OR "socioeconomic posit*" OR "socio economic position" OR "socio economic positions" OR "socio economic posit*" OR "social economic position" OR "social economic posit*" OR "socioeconomic status" OR "socio economic status" OR "social economic status" OR "deprived urban area" OR "deprived suburban area" OR "deprived area" OR "blue collar" OR "blue-collar" OR "deprive*" OR "deprived" OR "disadvantaged" OR "education level*" OR "educational level" OR "employment*" OR "income*" OR "job" OR "jobs" OR "low education" OR "low income" OR "low-educat*" OR "low-income" OR "social class*" OR "social disparity" OR "social disparities" OR "social disparit*" OR "social inequalit*" OR "social inequalities" OR "social inequality" OR "social inequit*" OR "social inequities" OR "social inequity" OR "health inequity" OR "health inequality" OR "health inequities" OR "health inequalities" OR "health inequit*" OR "health inequalit*" OR "Healthcare Disparity" OR "Healthcare Disparities" OR "Health care Disparity" OR "Health care Disparities" OR "social position" OR "social standing" OR "social status" OR "social strata" OR "socioeconomic" OR "socio-economic" OR "socio-economic" OR "socioeconomic factor" OR "socioeconomic factors" OR "Socio-economic factors" OR "Socio-economic factor" OR "socio-economic status" OR "socioeconomically" OR "socio-economically" OR "underprivileged" OR "unemployed" OR "working class" OR "working-class " OR "years of education" OR "years of schooling" OR "job status" OR "occupational status" OR "occupation status")) AND (TI=("Exercise" OR "Physical activity" OR "Physical activities" OR "Exercise" OR "Exercises" OR "Exercising" OR "Exercis*" OR "Strength training" OR "Aerobic" OR "Aerobics" OR "Resistance training" OR "Walking" OR "Endurance Training" OR "Exergaming" OR "Gymnastics" OR "Interval Training" OR "Jogging" OR "Nordic Walking" OR "Physical Conditioning" OR "Running" OR "Stair Climbing" OR "Swimming" OR "Sitting" OR "Sedentary Behavior" OR "Sedentary behaviour" OR "Sedentary behavior" OR "Activity" OR "activities" OR "Inactivity" OR "inactivities" OR "Inactivit*" OR "Diet" OR "diet" OR "diets" OR "diet*" OR "nutrition" OR "nutritional" OR "nutrition*" OR "Eating" OR "eat" OR "eating" OR "consumption" OR "food intake" OR "food pattern" OR "food habit" OR "food patterns" OR "food habits" OR "food intake" OR "Food" OR "food" OR "foods" OR "food-related" OR "vegetable" OR "vegetables" OR "fruit" OR "fruits" OR "wholegrain" OR "wholegrains" OR "legume*" OR "nut" OR "nuts" OR "dairy" OR "fish" OR "tea" OR "fat" OR "fats" OR "oil" OR "oils" OR "coffee" OR "red meat" OR "processed meat" OR "Food and Beverages" OR "Beverages" OR "sweetened beverage*" OR "juice*" OR "Drinking Behavior" OR "Drinking Behavior" OR "Drinking Behaviour" OR "Alcohol Drinking" OR "Ethanol" OR "Ethanol" OR "alcohol" OR "Alcohol consumption" OR "Alcohol drinking" OR "alcohol use" OR "Energy Intake" OR "Weight Gain" OR "Weight gain" OR "gain weight" OR "gaining weight" OR "poor diet" OR "poor diets" OR "poor dietary" OR "healthy eating" OR "Smoking" OR "Smoking" OR "Smoking Cessation" OR "Smoking Reduction" OR "Tobacco Use Cessation" OR "Smoking cessation" OR "Tobacco" OR "Tobacco Products" OR "Tobacco use" OR "Tobacco use") OR AK=("Exercise" OR "Physical activity" OR "Physical activities" OR "Exercise" OR "Exercises" OR "Exercising" OR "Exercis*" OR "Strength training" OR "Aerobic" OR "Aerobics" OR "Resistance training" OR "Walking" OR "Endurance Training" OR "Exergaming" OR "Gymnastics" OR "Interval Training" OR "Jogging" OR "Nordic Walking" OR "Physical Conditioning" OR "Running" OR "Stair Climbing" OR "Swimming" OR "Sitting" OR "Sedentary Behavior" OR "Sedentary behaviour" OR "Sedentary behavior" OR "Activity" OR "activities" OR "Inactivity" OR "inactivities" OR "Inactivit*" OR "Diet" OR "diet" OR "diets" OR "diet*" OR "nutrition" OR "nutritional" OR "nutrition*" OR "Eating" OR "eat" OR "eating" OR "consumption" OR "food intake" OR "food pattern" OR "food habit" OR "food patterns" OR "food habits" OR "food intake" OR "Food" OR "food" OR "foods" OR "food-related" OR "vegetable" OR "vegetables" OR "fruit" OR "fruits" OR "wholegrain" OR "wholegrains" OR "legume*" OR "nut" OR "nuts" OR "dairy" OR "fish" OR "tea" OR "fat" OR "fats" OR "oil" OR "oils" OR "coffee" OR "red meat" OR "processed meat" OR "Food and Beverages" OR "Beverages" OR "sweetened beverage*" OR "juice*" OR "Drinking Behavior" OR "Drinking Behavior" OR "Drinking Behaviour" OR "Alcohol Drinking" OR "Ethanol" OR "Ethanol" OR "alcohol" OR "Alcohol consumption" OR "Alcohol drinking" OR "alcohol use" OR "Energy Intake" OR "Weight Gain" OR "Weight gain" OR "gain weight" OR "gaining weight" OR "poor diet" OR "poor diets" OR "poor dietary" OR "healthy eating" OR "Smoking" OR "Smoking" OR "Smoking Cessation" OR "Smoking Reduction" OR "Tobacco Use Cessation" OR "Smoking cessation" OR "Tobacco" OR "Tobacco Products" OR "Tobacco use" OR "Tobacco use") OR AB=("Exercise" OR "Physical activity" OR "Physical activities" OR "Exercise" OR "Exercises" OR "Exercising" OR "Exercis*" OR "Strength training" OR "Aerobic" OR "Aerobics" OR "Resistance training" OR "Walking" OR "Endurance Training" OR "Exergaming" OR "Gymnastics" OR "Interval Training" OR "Jogging" OR "Nordic Walking" OR "Physical Conditioning" OR "Running" OR "Stair Climbing" OR "Swimming" OR "Sitting" OR "Sedentary Behavior" OR "Sedentary behaviour" OR "Sedentary behavior" OR "Activity" OR "activities" OR "Inactivity" OR "inactivities" OR "Inactivit*" OR "Diet" OR "diet" OR "diets" OR "diet*" OR "nutrition" OR "nutritional" OR "nutrition*" OR "Eating" OR "eat" OR "eating" OR "consumption" OR "food intake" OR "food pattern" OR "food habit" OR "food patterns" OR "food habits" OR "food intake" OR "Food" OR "food" OR "foods" OR "food-related" OR "vegetable" OR "vegetables" OR "fruit" OR "fruits" OR "wholegrain" OR "wholegrains" OR "legume*" OR "nut" OR "nuts" OR "dairy" OR "fish" OR "tea" OR "fat" OR "fats" OR "oil" OR "oils" OR "coffee" OR "red meat" OR "processed meat" OR "Food and Beverages" OR "Beverages" OR "sweetened beverage*" OR "juice*" OR "Drinking Behavior" OR "Drinking Behavior" OR "Drinking Behaviour" OR "Alcohol Drinking" OR "Ethanol" OR "Ethanol" OR "alcohol" OR "Alcohol consumption" OR "Alcohol drinking" OR "alcohol use" OR "Energy Intake" OR "Weight Gain" OR "Weight gain" OR "gain weight" OR "gaining weight" OR "poor diet" OR "poor diets" OR "poor dietary" OR "healthy eating" OR "Smoking" OR "Smoking" OR "Smoking Cessation" OR "Smoking Reduction" OR "Tobacco Use Cessation" OR "Smoking cessation" OR "Tobacco" OR "Tobacco Products" OR "Tobacco use" OR "Tobacco use")) AND (TI=("behavior change" OR "behavior changes" OR "behavior chang*" OR "behavioral change" OR "behavioral changes" OR "behavioral chang*" OR "behaviour change" OR "behaviour changes" OR "behaviour chang*" OR "behavioural change" OR "behavioural changes" OR "behavioural chang*" OR "behavior change interventions" OR "behavior change intervention" OR "behaviour change interventions" OR "behaviour change intervention" OR "behavioral change interventions" OR "behavioral change intervention" OR "behavioural change interventions" OR "behavioural change intervention" OR "behavior change techniques" OR "behavior change technique" OR "behaviour change techniques" OR "behaviour change technique" OR "behavioral change techniques" OR "behavioral change technique" OR "behavioural change techniques" OR "behavioural change technique" OR "Behavior Change" OR "Behavior Changes" OR "Behavior experiment" OR "Behavior experiments" OR "Behavior Intervention" OR "Behavior Interventions" OR "Behavior Modification" OR "Behavior Modifications" OR "Behavior Program" OR "Behavior Programme" OR "Behavior Programmes" OR "Behavior Programs" OR "Behavior Promotion" OR "Behavior Promotions" OR "Behavior Trial" OR "Behavior Trials" OR "Behavioral Change" OR "Behavioral Changes" OR "Behavioral experiment" OR "Behavioral experiments" OR "Behavioral Intervention" OR "Behavioral Interventions" OR "Behavioral Modification" OR "Behavioral Modifications" OR "Behavioral Program" OR "Behavioral Programme" OR "Behavioral Programmes" OR "Behavioral Programs" OR "Behavioral Promotion" OR "Behavioral Promotions" OR "Behavioral Trial" OR "Behavioral Trials" OR "Behaviors Change" OR "Behaviors Changes" OR "Behaviors experiment" OR "Behaviors experiments" OR "Behaviors Intervention" OR "Behaviors Interventions" OR "Behaviors Modification" OR "Behaviors Modifications" OR "Behaviors Program" OR "Behaviors Programme" OR "Behaviors Programmes" OR "Behaviors Programs" OR "Behaviors Promotion" OR "Behaviors Promotions" OR "Behaviors Trial" OR "Behaviors Trials" OR "Behaviour Change" OR "Behaviour Changes" OR "Behaviour experiment" OR "Behaviour experiments" OR "Behaviour Intervention" OR "Behaviour Interventions" OR "Behaviour Modification" OR "Behaviour Modifications" OR "Behaviour Program" OR "Behaviour Programme" OR "Behaviour Programmes" OR "Behaviour Programs" OR "Behaviour Promotion" OR "Behaviour Promotions" OR "Behaviour Trial" OR "Behaviour Trials" OR "Behavioural Change" OR "Behavioural Changes" OR "Behavioural experiment" OR "Behavioural experiments" OR "Behavioural Intervention" OR "Behavioural Interventions" OR "Behavioural Modification" OR "Behavioural Modifications" OR "Behavioural Program" OR "Behavioural Programme" OR "Behavioural Programmes" OR "Behavioural Programs" OR "Behavioural Promotion" OR "Behavioural Promotions" OR "Behavioural Trial" OR "Behavioural Trials" OR "Behaviours Change" OR "Behaviours Changes" OR "Behaviours experiment" OR "Behaviours experiments" OR "Behaviours Intervention" OR "Behaviours Interventions" OR "Behaviours Modification" OR "Behaviours Modifications" OR "Behaviours Program" OR "Behaviours Programme" OR "Behaviours Programmes" OR "Behaviours Programs" OR "Behaviours Promotion" OR "Behaviours Promotions" OR "Behaviours Trial" OR "Behaviours Trials") OR AK=("behavior change" OR "behavior changes" OR "behavior chang*" OR "behavioral change" OR "behavioral changes" OR "behavioral chang*" OR "behaviour change" OR "behaviour changes" OR "behaviour chang*" OR "behavioural change" OR "behavioural changes" OR "behavioural chang*" OR "behavior change interventions" OR "behavior change intervention" OR "behaviour change interventions" OR "behaviour change intervention" OR "behavioral change interventions" OR "behavioral change intervention" OR "behavioural change interventions" OR "behavioural change intervention" OR "behavior change techniques" OR "behavior change technique" OR "behaviour change techniques" OR "behaviour change technique" OR "behavioral change techniques" OR "behavioral change technique" OR "behavioural change techniques" OR "behavioural change technique" OR "Behavior Change" OR "Behavior Changes" OR "Behavior experiment" OR "Behavior experiments" OR "Behavior Intervention" OR "Behavior Interventions" OR "Behavior Modification" OR "Behavior Modifications" OR "Behavior Program" OR "Behavior Programme" OR "Behavior Programmes" OR "Behavior Programs" OR "Behavior Promotion" OR "Behavior Promotions" OR "Behavior Trial" OR "Behavior Trials" OR "Behavioral Change" OR "Behavioral Changes" OR "Behavioral experiment" OR "Behavioral experiments" OR "Behavioral Intervention" OR "Behavioral Interventions" OR "Behavioral Modification" OR "Behavioral Modifications" OR "Behavioral Program" OR "Behavioral Programme" OR "Behavioral Programmes" OR "Behavioral Programs" OR "Behavioral Promotion" OR "Behavioral Promotions" OR "Behavioral Trial" OR "Behavioral Trials" OR "Behaviors Change" OR "Behaviors Changes" OR "Behaviors experiment" OR "Behaviors experiments" OR "Behaviors Intervention" OR "Behaviors Interventions" OR "Behaviors Modification" OR "Behaviors Modifications" OR "Behaviors Program" OR "Behaviors Programme" OR "Behaviors Programmes" OR "Behaviors Programs" OR "Behaviors Promotion" OR "Behaviors Promotions" OR "Behaviors Trial" OR "Behaviors Trials" OR "Behaviour Change" OR "Behaviour Changes" OR "Behaviour experiment" OR "Behaviour experiments" OR "Behaviour Intervention" OR "Behaviour Interventions" OR "Behaviour Modification" OR "Behaviour Modifications" OR "Behaviour Program" OR "Behaviour Programme" OR "Behaviour Programmes" OR "Behaviour Programs" OR "Behaviour Promotion" OR "Behaviour Promotions" OR "Behaviour Trial" OR "Behaviour Trials" OR "Behavioural Change" OR "Behavioural Changes" OR "Behavioural experiment" OR "Behavioural experiments" OR "Behavioural Intervention" OR "Behavioural Interventions" OR "Behavioural Modification" OR "Behavioural Modifications" OR "Behavioural Program" OR "Behavioural Programme" OR "Behavioural Programmes" OR "Behavioural Programs" OR "Behavioural Promotion" OR "Behavioural Promotions" OR "Behavioural Trial" OR "Behavioural Trials" OR "Behaviours Change" OR "Behaviours Changes" OR "Behaviours experiment" OR "Behaviours experiments" OR "Behaviours Intervention" OR "Behaviours Interventions" OR "Behaviours Modification" OR "Behaviours Modifications" OR "Behaviours Program" OR "Behaviours Programme" OR "Behaviours Programmes" OR "Behaviours Programs" OR "Behaviours Promotion" OR "Behaviours Promotions" OR "Behaviours Trial" OR "Behaviours Trials") OR AB=("behavior change" OR "behavior changes" OR "behavior chang*" OR "behavioral change" OR "behavioral changes" OR "behavioral chang*" OR "behaviour change" OR "behaviour changes" OR "behaviour chang*" OR "behavioural change" OR "behavioural changes" OR "behavioural chang*" OR "behavior change interventions" OR "behavior change intervention" OR "behaviour change interventions" OR "behaviour change intervention" OR "behavioral change interventions" OR "behavioral change intervention" OR "behavioural change interventions" OR "behavioural change intervention" OR "behavior change techniques" OR "behavior change technique" OR "behaviour change techniques" OR "behaviour change technique" OR "behavioral change techniques" OR "behavioral change technique" OR "behavioural change techniques" OR "behavioural change technique" OR "Behavior Change" OR "Behavior Changes" OR "Behavior experiment" OR "Behavior experiments" OR "Behavior Intervention" OR "Behavior Interventions" OR "Behavior Modification" OR "Behavior Modifications" OR "Behavior Program" OR "Behavior Programme" OR "Behavior Programmes" OR "Behavior Programs" OR "Behavior Promotion" OR "Behavior Promotions" OR "Behavior Trial" OR "Behavior Trials" OR "Behavioral Change" OR "Behavioral Changes" OR "Behavioral experiment" OR "Behavioral experiments" OR "Behavioral Intervention" OR "Behavioral Interventions" OR "Behavioral Modification" OR "Behavioral Modifications" OR "Behavioral Program" OR "Behavioral Programme" OR "Behavioral Programmes" OR "Behavioral Programs" OR "Behavioral Promotion" OR "Behavioral Promotions" OR "Behavioral Trial" OR "Behavioral Trials" OR "Behaviors Change" OR "Behaviors Changes" OR "Behaviors experiment" OR "Behaviors experiments" OR "Behaviors Intervention" OR "Behaviors Interventions" OR "Behaviors Modification" OR "Behaviors Modifications" OR "Behaviors Program" OR "Behaviors Programme" OR "Behaviors Programmes" OR "Behaviors Programs" OR "Behaviors Promotion" OR "Behaviors Promotions" OR "Behaviors Trial" OR "Behaviors Trials" OR "Behaviour Change" OR "Behaviour Changes" OR "Behaviour experiment" OR "Behaviour experiments" OR "Behaviour Intervention" OR "Behaviour Interventions" OR "Behaviour Modification" OR "Behaviour Modifications" OR "Behaviour Program" OR "Behaviour Programme" OR "Behaviour Programmes" OR "Behaviour Programs" OR "Behaviour Promotion" OR "Behaviour Promotions" OR "Behaviour Trial" OR "Behaviour Trials" OR "Behavioural Change" OR "Behavioural Changes" OR "Behavioural experiment" OR "Behavioural experiments" OR "Behavioural Intervention" OR "Behavioural Interventions" OR "Behavioural Modification" OR "Behavioural Modifications" OR "Behavioural Program" OR "Behavioural Programme" OR "Behavioural Programmes" OR "Behavioural Programs" OR "Behavioural Promotion" OR "Behavioural Promotions" OR "Behavioural Trial" OR "Behavioural Trials" OR "Behaviours Change" OR "Behaviours Changes" OR "Behaviours experiment" OR "Behaviours experiments" OR "Behaviours Intervention" OR "Behaviours Interventions" OR "Behaviours Modification" OR "Behaviours Modifications" OR "Behaviours Program" OR "Behaviours Programme" OR "Behaviours Programmes" OR "Behaviours Programs" OR "Behaviours Promotion" OR "Behaviours Promotions" OR "Behaviours Trial" OR "Behaviours Trials") OR TI=(("promotion" OR "promote" OR "promoting") AND ("intervention" OR "interventions")) OR TI=(("change" OR "changes" OR "chang*" OR "experiment" OR "experiments" OR "Intervention" OR "Interventions" OR "Modification" OR "Modifications" OR "Program" OR "Programme" OR "Programmes" OR "Programs" OR "Promotion" OR "Promotions" OR "Trial" OR "Trials" OR "influence" OR "influences" OR "influencing" OR "influenced" OR "influenc*") AND ("behavior" OR "behaviors" OR "behavioral" OR "behavior*" OR "behaviour" OR "behaviours" OR "behavioural" OR "behaviour*") AND ("Exercise" OR "Physical activity" OR "Physical activities" OR "Exercise" OR "Exercises" OR "Exercising" OR "Exercis*" OR "Strength training" OR "Aerobic" OR "Aerobics" OR "Resistance training" OR "Walking" OR "Endurance Training" OR "Exergaming" OR "Gymnastics" OR "Interval Training" OR "Jogging" OR "Nordic Walking" OR "Physical Conditioning" OR "Running" OR "Stair Climbing" OR "Swimming" OR "Sitting" OR "Sedentary Behavior" OR "Sedentary behaviour" OR "Sedentary behavior" OR "Activity" OR "activities" OR "Inactivity" OR "inactivities" OR "Inactivit*" OR "Diet" OR "diet" OR "diets" OR "diet*" OR "nutrition" OR "nutritional" OR "nutrition*" OR "Eating" OR "eat" OR "eating" OR "consumption" OR "food intake" OR "food pattern" OR "food habit" OR "food patterns" OR "food habits" OR "food intake" OR "Food" OR "food" OR "foods" OR "food-related" OR "vegetable" OR "vegetables" OR "fruit" OR "fruits" OR "wholegrain" OR "wholegrains" OR "legume*" OR "nut" OR "nuts" OR "dairy" OR "fish" OR "tea" OR "fat" OR "fats" OR "oil" OR "oils" OR "coffee" OR "red meat" OR "processed meat" OR "Food and Beverages" OR "Beverages" OR "sweetened beverage*" OR "juice*" OR "Drinking Behavior" OR "Drinking Behavior" OR "Drinking Behaviour" OR "Alcohol Drinking" OR "Ethanol" OR "Ethanol" OR "alcohol" OR "Alcohol consumption" OR "Alcohol drinking" OR "alcohol use" OR "Energy Intake" OR "Weight Gain" OR "Weight gain" OR "gain weight" OR "gaining weight" OR "poor diet" OR "poor diets" OR "poor dietary" OR "healthy eating" OR "Smoking" OR "Smoking" OR "Smoking Cessation" OR "Smoking Reduction" OR "Tobacco Use Cessation" OR "Smoking cessation" OR "Tobacco" OR "Tobacco Products" OR "Tobacco use" OR "Tobacco use")) OR (AB=(("change" OR "changes" OR "chang*" OR "experiment" OR "experiments" OR "Intervention" OR "Interventions" OR "Modification" OR "Modifications" OR "Program" OR "Programme" OR "Programmes" OR "Programs" OR "Promotion" OR "Promotions" OR "Trial" OR "Trials" OR "influence" OR "influences" OR "influencing" OR "influenced" OR "influenc*") NEAR/2 ("behavior" OR "behaviors" OR "behavioral" OR "behavior*" OR "behaviour" OR "behaviours" OR "behavioural" OR "behaviour*") NEAR/2 ("Exercise" OR "Physical activity" OR "Physical activities" OR "Exercise" OR "Exercises" OR "Exercising" OR "Exercis*" OR "Strength training" OR "Aerobic" OR "Aerobics" OR "Resistance training" OR "Walking" OR "Endurance Training" OR "Exergaming" OR "Gymnastics" OR "Interval Training" OR "Jogging" OR "Nordic Walking" OR "Physical Conditioning" OR "Running" OR "Stair Climbing" OR "Swimming" OR "Sitting" OR "Sedentary Behavior" OR "Sedentary behaviour" OR "Sedentary behavior" OR "Activity" OR "activities" OR "Inactivity" OR "inactivities" OR "Inactivit*" OR "Diet" OR "diet" OR "diets" OR "diet*" OR "nutrition" OR "nutritional" OR "nutrition*" OR "Eating" OR "eat" OR "eating" OR "consumption" OR "food intake" OR "food pattern" OR "food habit" OR "food patterns" OR "food habits" OR "food intake" OR "Food" OR "food" OR "foods" OR "food-related" OR "vegetable" OR "vegetables" OR "fruit" OR "fruits" OR "wholegrain" OR "wholegrains" OR "legume*" OR "nut" OR "nuts" OR "dairy" OR "fish" OR "tea" OR "fat" OR "fats" OR "oil" OR "oils" OR "coffee" OR "red meat" OR "processed meat" OR "Food and Beverages" OR "Beverages" OR "sweetened beverage*" OR "juice*" OR "Drinking Behavior" OR "Drinking Behavior" OR "Drinking Behaviour" OR "Alcohol Drinking" OR "Ethanol" OR "Ethanol" OR "alcohol" OR "Alcohol consumption" OR "Alcohol drinking" OR "alcohol use" OR "Energy Intake" OR "Weight Gain" OR "Weight gain" OR "gain weight" OR "gaining weight" OR "poor diet" OR "poor diets" OR "poor dietary" OR "healthy eating" OR "Smoking" OR "Smoking" OR "Smoking Cessation" OR "Smoking Reduction" OR "Tobacco Use Cessation" OR "Smoking cessation" OR "Tobacco" OR "Tobacco Products" OR "Tobacco use" OR "Tobacco use")))) NOT TI=("Telemedicine" OR "e-health" OR "m-health" OR "ehealth" OR "mhealth" OR "telehealth" OR "schoolbased" OR "school based" OR "digital intervention" OR "digital interventions" OR "digital" OR "online" OR "internet" OR "smartphone" OR "smartphones" OR "phone" OR "phones") AND PY=(2000 OR 2001 OR 2002 OR 2003 OR 2004 OR 2005 OR 2006 OR 2007 OR 2008 OR 2009 OR 2010 OR 2011 OR 2012 OR 2013 OR 2014 OR 2015 OR 2016 OR 2017 OR 2018 OR 2019 OR 2020 OR 2021 OR 2022 OR 2023))

**Cochrane Library (Wiley)**

AND PY=(2000 OR 2001 OR 2002 OR 2003 OR 2004 OR 2005 OR 2006 OR 2007 OR 2008 OR 2009 OR 2010 OR 2011 OR 2012 OR 2013 OR 2014 OR 2015 OR 2016 OR 2017 OR 2018 OR 2019 OR 2020 OR 2021 OR 2022 OR 2023)

(("Economic Status" OR "Educational Status" OR "Employment" OR "Unemployment" OR "Income" OR "Salaries and Fringe Benefits" OR "Occupations" OR "Poverty" OR "Poverty Areas" OR "Social Class" OR "Social Conditions" OR "Economic Status" OR "Educational Status" OR "Employment" OR "Income" OR "Occupation" OR "Occupations" OR "Poverty" OR "Poverty Area" OR "Poverty Areas" OR "Salaries" OR "Salary" OR "Social Class" OR "Social Classes" OR "Social Condition" OR "Social Conditions" OR "Unemployment" OR "socioeconomic position" OR "socioeconomic positions" OR "socioeconomic posit*" OR "socio economic position" OR "socio economic positions" OR "socio economic posit*" OR "social economic position" OR "social economic posit*" OR "socioeconomic status" OR "socio economic status" OR "social economic status" OR "deprived urban area" OR "deprived suburban area" OR "deprived area" OR "blue collar" OR "blue collar" OR "deprive*" OR "deprived" OR "disadvantaged" OR "education level*" OR "educational level" OR "employment*" OR "income*" OR "job" OR "jobs" OR "low education" OR "low income" OR "low educat*" OR "low income" OR "social class*" OR "social disparity" OR "social disparities" OR "social disparit*" OR "social inequalit*" OR "social inequalities" OR "social inequality" OR "social inequit*" OR "social inequities" OR "social inequity" OR "health inequity" OR "health inequality" OR "health inequities" OR "health inequalities" OR "health inequit*" OR "health inequalit*" OR "Healthcare Disparity" OR "Healthcare Disparities" OR "Health care Disparity" OR "Health care Disparities" OR "social position" OR "social standing" OR "social status" OR "social strata" OR "socioeconomic" OR "socio economic" OR "socio economic" OR "socioeconomic factor" OR "socioeconomic factors" OR "Socio economic factors" OR "Socio economic factor" OR "socio economic status" OR "socioeconomically" OR "socio economically" OR "underprivileged" OR "unemployed" OR "working class" OR "working class " OR "years of education" OR "years of schooling" OR "job status" OR "occupational status" OR "occupation status"):ti,ab,kw AND ("Exercise" OR "Physical activity" OR "Physical activities" OR "Exercise" OR "Exercises" OR "Exercising" OR "Exercis*" OR "Strength training" OR "Aerobic" OR "Aerobics" OR "Resistance training" OR "Walking" OR "Endurance Training" OR "Exergaming" OR "Gymnastics" OR "Interval Training" OR "Jogging" OR "Nordic Walking" OR "Physical Conditioning" OR "Running" OR "Stair Climbing" OR "Swimming" OR "Sitting" OR "Sedentary Behavior" OR "Sedentary behaviour" OR "Sedentary behavior" OR "Activity" OR "activities" OR "Inactivity" OR "inactivities" OR "Inactivit*" OR "Diet" OR "diet" OR "diets" OR "diet*" OR "nutrition" OR "nutritional" OR "nutrition*" OR "Eating" OR "eat" OR "eating" OR "consumption" OR "food intake" OR "food pattern" OR "food habit" OR "food patterns" OR "food habits" OR "food intake" OR "Food" OR "food" OR "foods" OR "food related" OR "vegetable" OR "vegetables" OR "fruit" OR "fruits" OR "wholegrain" OR "wholegrains" OR "legume*" OR "nut" OR "nuts" OR "dairy" OR "fish" OR "tea" OR "fat" OR "fats" OR "oil" OR "oils" OR "coffee" OR "red meat" OR "processed meat" OR "Food and Beverages" OR "Beverages" OR "sweetened beverage*" OR "juice*" OR "Drinking Behavior" OR "Drinking Behavior" OR "Drinking Behaviour" OR "Alcohol Drinking" OR "Ethanol" OR "Ethanol" OR "alcohol" OR "Alcohol consumption" OR "Alcohol drinking" OR "alcohol use" OR "Energy Intake" OR "Weight Gain" OR "Weight gain" OR "gain weight" OR "gaining weight" OR "poor diet" OR "poor diets" OR "poor dietary" OR "healthy eating" OR "Smoking" OR "Smoking" OR "Smoking Cessation" OR "Smoking Reduction" OR "Tobacco Use Cessation" OR "Smoking cessation" OR "Tobacco" OR "Tobacco Products" OR "Tobacco use" OR "Tobacco use"):ti,ab,kw AND (("behavior change" OR "behavior changes" OR "behavior chang*" OR "behavioral change" OR "behavioral changes" OR "behavioral chang*" OR "behaviour change" OR "behaviour changes" OR "behaviour chang*" OR "behavioural change" OR "behavioural changes" OR "behavioural chang*" OR "behavior change interventions" OR "behavior change intervention" OR "behaviour change interventions" OR "behaviour change intervention" OR "behavioral change interventions" OR "behavioral change intervention" OR "behavioural change interventions" OR "behavioural change intervention" OR "behavior change techniques" OR "behavior change technique" OR "behaviour change techniques" OR "behaviour change technique" OR "behavioral change techniques" OR "behavioral change technique" OR "behavioural change techniques" OR "behavioural change technique" OR "Behavior Change" OR "Behavior Changes" OR "Behavior experiment" OR "Behavior experiments" OR "Behavior Intervention" OR "Behavior Interventions" OR "Behavior Modification" OR "Behavior Modifications" OR "Behavior Program" OR "Behavior Programme" OR "Behavior Programmes" OR "Behavior Programs" OR "Behavior Promotion" OR "Behavior Promotions" OR "Behavior Trial" OR "Behavior Trials" OR "Behavioral Change" OR "Behavioral Changes" OR "Behavioral experiment" OR "Behavioral experiments" OR "Behavioral Intervention" OR "Behavioral Interventions" OR "Behavioral Modification" OR "Behavioral Modifications" OR "Behavioral Program" OR "Behavioral Programme" OR "Behavioral Programmes" OR "Behavioral Programs" OR "Behavioral Promotion" OR "Behavioral Promotions" OR "Behavioral Trial" OR "Behavioral Trials" OR "Behaviors Change" OR "Behaviors Changes" OR "Behaviors experiment" OR "Behaviors experiments" OR "Behaviors Intervention" OR "Behaviors Interventions" OR "Behaviors Modification" OR "Behaviors Modifications" OR "Behaviors Program" OR "Behaviors Programme" OR "Behaviors Programmes" OR "Behaviors Programs" OR "Behaviors Promotion" OR "Behaviors Promotions" OR "Behaviors Trial" OR "Behaviors Trials" OR "Behaviour Change" OR "Behaviour Changes" OR "Behaviour experiment" OR "Behaviour experiments" OR "Behaviour Intervention" OR "Behaviour Interventions" OR "Behaviour Modification" OR "Behaviour Modifications" OR "Behaviour Program" OR "Behaviour Programme" OR "Behaviour Programmes" OR "Behaviour Programs" OR "Behaviour Promotion" OR "Behaviour Promotions" OR "Behaviour Trial" OR "Behaviour Trials" OR "Behavioural Change" OR "Behavioural Changes" OR "Behavioural experiment" OR "Behavioural experiments" OR "Behavioural Intervention" OR "Behavioural Interventions" OR "Behavioural Modification" OR "Behavioural Modifications" OR "Behavioural Program" OR "Behavioural Programme" OR "Behavioural Programmes" OR "Behavioural Programs" OR "Behavioural Promotion" OR "Behavioural Promotions" OR "Behavioural Trial" OR "Behavioural Trials" OR "Behaviours Change" OR "Behaviours Changes" OR "Behaviours experiment" OR "Behaviours experiments" OR "Behaviours Intervention" OR "Behaviours Interventions" OR "Behaviours Modification" OR "Behaviours Modifications" OR "Behaviours Program" OR "Behaviours Programme" OR "Behaviours Programmes" OR "Behaviours Programs" OR "Behaviours Promotion" OR "Behaviours Promotions" OR "Behaviours Trial" OR "Behaviours Trials"):ti,ab,kw OR (("promotion" OR "promote" OR "promoting") AND ("intervention" OR "interventions")):ti) NOT ("Telemedicine" OR "e health" OR "m health" OR "ehealth" OR "mhealth" OR "telehealth" OR "schoolbased" OR "school based" OR "digital intervention" OR "digital interventions" OR "digital" OR "online" OR "internet" OR "smartphone" OR "smartphones" OR "phone" OR "phones"):ti) OR (("Economic Status" OR "Educational Status" OR "Employment" OR "Unemployment" OR "Income" OR "Salaries and Fringe Benefits" OR "Occupations" OR "Poverty" OR "Poverty Areas" OR "Social Class" OR "Social Conditions" OR "Economic Status" OR "Educational Status" OR "Employment" OR "Income" OR "Occupation" OR "Occupations" OR "Poverty" OR "Poverty Area" OR "Poverty Areas" OR "Salaries" OR "Salary" OR "Social Class" OR "Social Classes" OR "Social Condition" OR "Social Conditions" OR "Unemployment" OR "socioeconomic position" OR "socioeconomic positions" OR "socioeconomic posit*" OR "socio economic position" OR "socio economic positions" OR "socio economic posit*" OR "social economic position" OR "social economic posit*" OR "socioeconomic status" OR "socio economic status" OR "social economic status" OR "deprived urban area" OR "deprived suburban area" OR "deprived area" OR "blue collar" OR "blue collar" OR "deprive*" OR "deprived" OR "disadvantaged" OR "education level*" OR "educational level" OR "employment*" OR "income*" OR "job" OR "jobs" OR "low education" OR "low income" OR "low educat*" OR "low income" OR "social class*" OR "social disparity" OR "social disparities" OR "social disparit*" OR "social inequalit*" OR "social inequalities" OR "social inequality" OR "social inequit*" OR "social inequities" OR "social inequity" OR "health inequity" OR "health inequality" OR "health inequities" OR "health inequalities" OR "health inequit*" OR "health inequalit*" OR "Healthcare Disparity" OR "Healthcare Disparities" OR "Health care Disparity" OR "Health care Disparities" OR "social position" OR "social standing" OR "social status" OR "social strata" OR "socioeconomic" OR "socio economic" OR "socio economic" OR "socioeconomic factor" OR "socioeconomic factors" OR "Socio economic factors" OR "Socio economic factor" OR "socio economic status" OR "socioeconomically" OR "socio economically" OR "underprivileged" OR "unemployed" OR "working class" OR "working class " OR "years of education" OR "years of schooling" OR "job status" OR "occupational status" OR "occupation status"):ti,ab,kw AND (("change" OR "changes" OR "chang*" OR "experiment" OR "experiments" OR "Intervention" OR "Interventions" OR "Modification" OR "Modifications" OR "Program" OR "Programme" OR "Programmes" OR "Programs" OR "Promotion" OR "Promotions" OR "Trial" OR "Trials" OR "influence" OR "influences" OR "influencing" OR "influenced" OR "influenc*") NEAR/2 ("behavior" OR "behaviors" OR "behavioral" OR "behavior*" OR "behaviour" OR "behaviours" OR "behavioural" OR "behaviour*") NEAR/2 ("Exercise" OR "Physical activity" OR "Physical activities" OR "Exercise" OR "Exercises" OR "Exercising" OR "Exercis*" OR "Strength training" OR "Aerobic" OR "Aerobics" OR "Resistance training" OR "Walking" OR "Endurance Training" OR "Exergaming" OR "Gymnastics" OR "Interval Training" OR "Jogging" OR "Nordic Walking" OR "Physical Conditioning" OR "Running" OR "Stair Climbing" OR "Swimming" OR "Sitting" OR "Sedentary Behavior" OR "Sedentary behaviour" OR "Sedentary behavior" OR "Activity" OR "activities" OR "Inactivity" OR "inactivities" OR "Inactivit*" OR "Diet" OR "diet" OR "diets" OR "diet*" OR "nutrition" OR "nutritional" OR "nutrition*" OR "Eating" OR "eat" OR "eating" OR "consumption" OR "food intake" OR "food pattern" OR "food habit" OR "food patterns" OR "food habits" OR "food intake" OR "Food" OR "food" OR "foods" OR "food-related" OR "vegetable" OR "vegetables" OR "fruit" OR "fruits" OR "wholegrain" OR "wholegrains" OR "legume*" OR "nut" OR "nuts" OR "dairy" OR "fish" OR "tea" OR "fat" OR "fats" OR "oil" OR "oils" OR "coffee" OR "red meat" OR "processed meat" OR "Food and Beverages" OR "Beverages" OR "sweetened beverage*" OR "juice*" OR "Drinking Behavior" OR "Drinking Behavior" OR "Drinking Behaviour" OR "Alcohol Drinking" OR "Ethanol" OR "Ethanol" OR "alcohol" OR "Alcohol consumption" OR "Alcohol drinking" OR "alcohol use" OR "Energy Intake" OR "Weight Gain" OR "Weight gain" OR "gain weight" OR "gaining weight" OR "poor diet" OR "poor diets" OR "poor dietary" OR "healthy eating" OR "Smoking" OR "Smoking" OR "Smoking Cessation" OR "Smoking Reduction" OR "Tobacco Use Cessation" OR "Smoking cessation" OR "Tobacco" OR "Tobacco Products" OR "Tobacco use" OR "Tobacco use")):ti,ab,kw NOT ("Telemedicine" OR "e health" OR "m health" OR "ehealth" OR "mhealth" OR "telehealth" OR "schoolbased" OR "school based" OR "digital intervention" OR "digital interventions" OR "digital" OR "online" OR "internet" OR "smartphone" OR "smartphones" OR "phone" OR "phones"):ti)

**PsycINFO / PsycArticles / Psychology and Behavioral Sciences Collection (EbscoHOST)**

AND PY=(2000 OR 2001 OR 2002 OR 2003 OR 2004 OR 2005 OR 2006 OR 2007 OR 2008 OR 2009 OR 2010 OR 2011 OR 2012 OR 2013 OR 2014 OR 2015 OR 2016 OR 2017 OR 2018 OR 2019 OR 2020 OR 2021 OR 2022 OR 2023)

((TI("Economic Status" OR "Educational Status" OR "Employment" OR "Unemployment" OR "Income" OR "Salaries and Fringe Benefits" OR "Occupations" OR "Poverty" OR "Poverty Areas" OR "Social Class" OR "Social Conditions" OR "Economic Status" OR "Educational Status" OR "Employment" OR "Income" OR "Occupation" OR "Occupations" OR "Poverty" OR "Poverty Area" OR "Poverty Areas" OR "Salaries" OR "Salary" OR "Social Class" OR "Social Classes" OR "Social Condition" OR "Social Conditions" OR "Unemployment" OR "socioeconomic position" OR "socioeconomic positions" OR "socioeconomic posit*" OR "socio economic position" OR "socio economic positions" OR "socio economic posit*" OR "social economic position" OR "social economic posit*" OR "socioeconomic status" OR "socio economic status" OR "social economic status" OR "deprived urban area" OR "deprived suburban area" OR "deprived area" OR "blue collar" OR "blue-collar" OR "deprive*" OR "deprived" OR "disadvantaged" OR "education level*" OR "educational level" OR "employment*" OR "income*" OR "job" OR "jobs" OR "low education" OR "low income" OR "low-educat*" OR "low-income" OR "social class*" OR "social disparity" OR "social disparities" OR "social disparit*" OR "social inequalit*" OR "social inequalities" OR "social inequality" OR "social inequit*" OR "social inequities" OR "social inequity" OR "health inequity" OR "health inequality" OR "health inequities" OR "health inequalities" OR "health inequit*" OR "health inequalit*" OR "Healthcare Disparity" OR "Healthcare Disparities" OR "Health care Disparity" OR "Health care Disparities" OR "social position" OR "social standing" OR "social status" OR "social strata" OR "socioeconomic" OR "socio-economic" OR "socio-economic" OR "socioeconomic factor" OR "socioeconomic factors" OR "Socio-economic factors" OR "Socio-economic factor" OR "socio-economic status" OR "socioeconomically" OR "socio-economically" OR "underprivileged" OR "unemployed" OR "working class" OR "working-class " OR "years of education" OR "years of schooling" OR "job status" OR "occupational status" OR "occupation status") OR MA("Economic Status" OR "Educational Status" OR "Employment" OR "Unemployment" OR "Income" OR "Salaries and Fringe Benefits" OR "Occupations" OR "Poverty" OR "Poverty Areas" OR "Social Class" OR "Social Conditions" OR "Economic Status" OR "Educational Status" OR "Employment" OR "Income" OR "Occupation" OR "Occupations" OR "Poverty" OR "Poverty Area" OR "Poverty Areas" OR "Salaries" OR "Salary" OR "Social Class" OR "Social Classes" OR "Social Condition" OR "Social Conditions" OR "Unemployment" OR "socioeconomic position" OR "socioeconomic positions" OR "socioeconomic posit*" OR "socio economic position" OR "socio economic positions" OR "socio economic posit*" OR "social economic position" OR "social economic posit*" OR "socioeconomic status" OR "socio economic status" OR "social economic status" OR "deprived urban area" OR "deprived suburban area" OR "deprived area" OR "blue collar" OR "blue-collar" OR "deprive*" OR "deprived" OR "disadvantaged" OR "education level*" OR "educational level" OR "employment*" OR "income*" OR "job" OR "jobs" OR "low education" OR "low income" OR "low-educat*" OR "low-income" OR "social class*" OR "social disparity" OR "social disparities" OR "social disparit*" OR "social inequalit*" OR "social inequalities" OR "social inequality" OR "social inequit*" OR "social inequities" OR "social inequity" OR "health inequity" OR "health inequality" OR "health inequities" OR "health inequalities" OR "health inequit*" OR "health inequalit*" OR "Healthcare Disparity" OR "Healthcare Disparities" OR "Health care Disparity" OR "Health care Disparities" OR "social position" OR "social standing" OR "social status" OR "social strata" OR "socioeconomic" OR "socio-economic" OR "socio-economic" OR "socioeconomic factor" OR "socioeconomic factors" OR "Socio-economic factors" OR "Socio-economic factor" OR "socio-economic status" OR "socioeconomically" OR "socio-economically" OR "underprivileged" OR "unemployed" OR "working class" OR "working-class " OR "years of education" OR "years of schooling" OR "job status" OR "occupational status" OR "occupation status") OR SU("Economic Status" OR "Educational Status" OR "Employment" OR "Unemployment" OR "Income" OR "Salaries and Fringe Benefits" OR "Occupations" OR "Poverty" OR "Poverty Areas" OR "Social Class" OR "Social Conditions" OR "Economic Status" OR "Educational Status" OR "Employment" OR "Income" OR "Occupation" OR "Occupations" OR "Poverty" OR "Poverty Area" OR "Poverty Areas" OR "Salaries" OR "Salary" OR "Social Class" OR "Social Classes" OR "Social Condition" OR "Social Conditions" OR "Unemployment" OR "socioeconomic position" OR "socioeconomic positions" OR "socioeconomic posit*" OR "socio economic position" OR "socio economic positions" OR "socio economic posit*" OR "social economic position" OR "social economic posit*" OR "socioeconomic status" OR "socio economic status" OR "social economic status" OR "deprived urban area" OR "deprived suburban area" OR "deprived area" OR "blue collar" OR "blue-collar" OR "deprive*" OR "deprived" OR "disadvantaged" OR "education level*" OR "educational level" OR "employment*" OR "income*" OR "job" OR "jobs" OR "low education" OR "low income" OR "low-educat*" OR "low-income" OR "social class*" OR "social disparity" OR "social disparities" OR "social disparit*" OR "social inequalit*" OR "social inequalities" OR "social inequality" OR "social inequit*" OR "social inequities" OR "social inequity" OR "health inequity" OR "health inequality" OR "health inequities" OR "health inequalities" OR "health inequit*" OR "health inequalit*" OR "Healthcare Disparity" OR "Healthcare Disparities" OR "Health care Disparity" OR "Health care Disparities" OR "social position" OR "social standing" OR "social status" OR "social strata" OR "socioeconomic" OR "socio-economic" OR "socio-economic" OR "socioeconomic factor" OR "socioeconomic factors" OR "Socio-economic factors" OR "Socio-economic factor" OR "socio-economic status" OR "socioeconomically" OR "socio-economically" OR "underprivileged" OR "unemployed" OR "working class" OR "working-class " OR "years of education" OR "years of schooling" OR "job status" OR "occupational status" OR "occupation status")) AND (TI("Exercise" OR "Physical activity" OR "Physical activities" OR "Exercise" OR "Exercises" OR "Exercising" OR "Exercis*" OR "Strength training" OR "Aerobic" OR "Aerobics" OR "Resistance training" OR "Walking" OR "Endurance Training" OR "Exergaming" OR "Gymnastics" OR "Interval Training" OR "Jogging" OR "Nordic Walking" OR "Physical Conditioning" OR "Running" OR "Stair Climbing" OR "Swimming" OR "Sitting" OR "Sedentary Behavior" OR "Sedentary behaviour" OR "Sedentary behavior" OR "Activity" OR "activities" OR "Inactivity" OR "inactivities" OR "Inactivit*" OR "Diet" OR "diet" OR "diets" OR "diet*" OR "nutrition" OR "nutritional" OR "nutrition*" OR "Eating" OR "eat" OR "eating" OR "consumption" OR "food intake" OR "food pattern" OR "food habit" OR "food patterns" OR "food habits" OR "food intake" OR "Food" OR "food" OR "foods" OR "food-related" OR "vegetable" OR "vegetables" OR "fruit" OR "fruits" OR "wholegrain" OR "wholegrains" OR "legume*" OR "nut" OR "nuts" OR "dairy" OR "fish" OR "tea" OR "fat" OR "fats" OR "oil" OR "oils" OR "coffee" OR "red meat" OR "processed meat" OR "Food and Beverages" OR "Beverages" OR "sweetened beverage*" OR "juice*" OR "Drinking Behavior" OR "Drinking Behavior" OR "Drinking Behaviour" OR "Alcohol Drinking" OR "Ethanol" OR "Ethanol" OR "alcohol" OR "Alcohol consumption" OR "Alcohol drinking" OR "alcohol use" OR "Energy Intake" OR "Weight Gain" OR "Weight gain" OR "gain weight" OR "gaining weight" OR "poor diet" OR "poor diets" OR "poor dietary" OR "healthy eating" OR "Smoking" OR "Smoking" OR "Smoking Cessation" OR "Smoking Reduction" OR "Tobacco Use Cessation" OR "Smoking cessation" OR "Tobacco" OR "Tobacco Products" OR "Tobacco use" OR "Tobacco use") OR MA("Exercise" OR "Physical activity" OR "Physical activities" OR "Exercise" OR "Exercises" OR "Exercising" OR "Exercis*" OR "Strength training" OR "Aerobic" OR "Aerobics" OR "Resistance training" OR "Walking" OR "Endurance Training" OR "Exergaming" OR "Gymnastics" OR "Interval Training" OR "Jogging" OR "Nordic Walking" OR "Physical Conditioning" OR "Running" OR "Stair Climbing" OR "Swimming" OR "Sitting" OR "Sedentary Behavior" OR "Sedentary behaviour" OR "Sedentary behavior" OR "Activity" OR "activities" OR "Inactivity" OR "inactivities" OR "Inactivit*" OR "Diet" OR "diet" OR "diets" OR "diet*" OR "nutrition" OR "nutritional" OR "nutrition*" OR "Eating" OR "eat" OR "eating" OR "consumption" OR "food intake" OR "food pattern" OR "food habit" OR "food patterns" OR "food habits" OR "food intake" OR "Food" OR "food" OR "foods" OR "food-related" OR "vegetable" OR "vegetables" OR "fruit" OR "fruits" OR "wholegrain" OR "wholegrains" OR "legume*" OR "nut" OR "nuts" OR "dairy" OR "fish" OR "tea" OR "fat" OR "fats" OR "oil" OR "oils" OR "coffee" OR "red meat" OR "processed meat" OR "Food and Beverages" OR "Beverages" OR "sweetened beverage*" OR "juice*" OR "Drinking Behavior" OR "Drinking Behavior" OR "Drinking Behaviour" OR "Alcohol Drinking" OR "Ethanol" OR "Ethanol" OR "alcohol" OR "Alcohol consumption" OR "Alcohol drinking" OR "alcohol use" OR "Energy Intake" OR "Weight Gain" OR "Weight gain" OR "gain weight" OR "gaining weight" OR "poor diet" OR "poor diets" OR "poor dietary" OR "healthy eating" OR "Smoking" OR "Smoking" OR "Smoking Cessation" OR "Smoking Reduction" OR "Tobacco Use Cessation" OR "Smoking cessation" OR "Tobacco" OR "Tobacco Products" OR "Tobacco use" OR "Tobacco use") OR SU("Exercise" OR "Physical activity" OR "Physical activities" OR "Exercise" OR "Exercises" OR "Exercising" OR "Exercis*" OR "Strength training" OR "Aerobic" OR "Aerobics" OR "Resistance training" OR "Walking" OR "Endurance Training" OR "Exergaming" OR "Gymnastics" OR "Interval Training" OR "Jogging" OR "Nordic Walking" OR "Physical Conditioning" OR "Running" OR "Stair Climbing" OR "Swimming" OR "Sitting" OR "Sedentary Behavior" OR "Sedentary behaviour" OR "Sedentary behavior" OR "Activity" OR "activities" OR "Inactivity" OR "inactivities" OR "Inactivit*" OR "Diet" OR "diet" OR "diets" OR "diet*" OR "nutrition" OR "nutritional" OR "nutrition*" OR "Eating" OR "eat" OR "eating" OR "consumption" OR "food intake" OR "food pattern" OR "food habit" OR "food patterns" OR "food habits" OR "food intake" OR "Food" OR "food" OR "foods" OR "food-related" OR "vegetable" OR "vegetables" OR "fruit" OR "fruits" OR "wholegrain" OR "wholegrains" OR "legume*" OR "nut" OR "nuts" OR "dairy" OR "fish" OR "tea" OR "fat" OR "fats" OR "oil" OR "oils" OR "coffee" OR "red meat" OR "processed meat" OR "Food and Beverages" OR "Beverages" OR "sweetened beverage*" OR "juice*" OR "Drinking Behavior" OR "Drinking Behavior" OR "Drinking Behaviour" OR "Alcohol Drinking" OR "Ethanol" OR "Ethanol" OR "alcohol" OR "Alcohol consumption" OR "Alcohol drinking" OR "alcohol use" OR "Energy Intake" OR "Weight Gain" OR "Weight gain" OR "gain weight" OR "gaining weight" OR "poor diet" OR "poor diets" OR "poor dietary" OR "healthy eating" OR "Smoking" OR "Smoking" OR "Smoking Cessation" OR "Smoking Reduction" OR "Tobacco Use Cessation" OR "Smoking cessation" OR "Tobacco" OR "Tobacco Products" OR "Tobacco use" OR "Tobacco use") OR AB("Exercise" OR "Physical activity" OR "Physical activities" OR "Exercise" OR "Exercises" OR "Exercising" OR "Exercis*" OR "Strength training" OR "Aerobic" OR "Aerobics" OR "Resistance training" OR "Walking" OR "Endurance Training" OR "Exergaming" OR "Gymnastics" OR "Interval Training" OR "Jogging" OR "Nordic Walking" OR "Physical Conditioning" OR "Running" OR "Stair Climbing" OR "Swimming" OR "Sitting" OR "Sedentary Behavior" OR "Sedentary behaviour" OR "Sedentary behavior" OR "Activity" OR "activities" OR "Inactivity" OR "inactivities" OR "Inactivit*" OR "Diet" OR "diet" OR "diets" OR "diet*" OR "nutrition" OR "nutritional" OR "nutrition*" OR "Eating" OR "eat" OR "eating" OR "consumption" OR "food intake" OR "food pattern" OR "food habit" OR "food patterns" OR "food habits" OR "food intake" OR "Food" OR "food" OR "foods" OR "food-related" OR "vegetable" OR "vegetables" OR "fruit" OR "fruits" OR "wholegrain" OR "wholegrains" OR "legume*" OR "nut" OR "nuts" OR "dairy" OR "fish" OR "tea" OR "fat" OR "fats" OR "oil" OR "oils" OR "coffee" OR "red meat" OR "processed meat" OR "Food and Beverages" OR "Beverages" OR "sweetened beverage*" OR "juice*" OR "Drinking Behavior" OR "Drinking Behavior" OR "Drinking Behaviour" OR "Alcohol Drinking" OR "Ethanol" OR "Ethanol" OR "alcohol" OR "Alcohol consumption" OR "Alcohol drinking" OR "alcohol use" OR "Energy Intake" OR "Weight Gain" OR "Weight gain" OR "gain weight" OR "gaining weight" OR "poor diet" OR "poor diets" OR "poor dietary" OR "healthy eating" OR "Smoking" OR "Smoking" OR "Smoking Cessation" OR "Smoking Reduction" OR "Tobacco Use Cessation" OR "Smoking cessation" OR "Tobacco" OR "Tobacco Products" OR "Tobacco use" OR "Tobacco use") OR SU("Exercise" OR "Physical activity" OR "Physical activities" OR "Exercise" OR "Exercises" OR "Exercising" OR "Exercis*" OR "Strength training" OR "Aerobic" OR "Aerobics" OR "Resistance training" OR "Walking" OR "Endurance Training" OR "Exergaming" OR "Gymnastics" OR "Interval Training" OR "Jogging" OR "Nordic Walking" OR "Physical Conditioning" OR "Running" OR "Stair Climbing" OR "Swimming" OR "Sitting" OR "Sedentary Behavior" OR "Sedentary behaviour" OR "Sedentary behavior" OR "Activity" OR "activities" OR "Inactivity" OR "inactivities" OR "Inactivit*" OR "Diet" OR "diet" OR "diets" OR "diet*" OR "nutrition" OR "nutritional" OR "nutrition*" OR "Eating" OR "eat" OR "eating" OR "consumption" OR "food intake" OR "food pattern" OR "food habit" OR "food patterns" OR "food habits" OR "food intake" OR "Food" OR "food" OR "foods" OR "food-related" OR "vegetable" OR "vegetables" OR "fruit" OR "fruits" OR "wholegrain" OR "wholegrains" OR "legume*" OR "nut" OR "nuts" OR "dairy" OR "fish" OR "tea" OR "fat" OR "fats" OR "oil" OR "oils" OR "coffee" OR "red meat" OR "processed meat" OR "Food and Beverages" OR "Beverages" OR "sweetened beverage*" OR "juice*" OR "Drinking Behavior" OR "Drinking Behavior" OR "Drinking Behaviour" OR "Alcohol Drinking" OR "Ethanol" OR "Ethanol" OR "alcohol" OR "Alcohol consumption" OR "Alcohol drinking" OR "alcohol use" OR "Energy Intake" OR "Weight Gain" OR "Weight gain" OR "gain weight" OR "gaining weight" OR "poor diet" OR "poor diets" OR "poor dietary" OR "healthy eating" OR "Smoking" OR "Smoking" OR "Smoking Cessation" OR "Smoking Reduction" OR "Tobacco Use Cessation" OR "Smoking cessation" OR "Tobacco" OR "Tobacco Products" OR "Tobacco use" OR "Tobacco use")) AND (TI("behavior change" OR "behavior changes" OR "behavior chang*" OR "behavioral change" OR "behavioral changes" OR "behavioral chang*" OR "behaviour change" OR "behaviour changes" OR "behaviour chang*" OR "behavioural change" OR "behavioural changes" OR "behavioural chang*" OR "behavior change interventions" OR "behavior change intervention" OR "behaviour change interventions" OR "behaviour change intervention" OR "behavioral change interventions" OR "behavioral change intervention" OR "behavioural change interventions" OR "behavioural change intervention" OR "behavior change techniques" OR "behavior change technique" OR "behaviour change techniques" OR "behaviour change technique" OR "behavioral change techniques" OR "behavioral change technique" OR "behavioural change techniques" OR "behavioural change technique" OR "Behavior Change" OR "Behavior Changes" OR "Behavior experiment" OR "Behavior experiments" OR "Behavior Intervention" OR "Behavior Interventions" OR "Behavior Modification" OR "Behavior Modifications" OR "Behavior Program" OR "Behavior Programme" OR "Behavior Programmes" OR "Behavior Programs" OR "Behavior Promotion" OR "Behavior Promotions" OR "Behavior Trial" OR "Behavior Trials" OR "Behavioral Change" OR "Behavioral Changes" OR "Behavioral experiment" OR "Behavioral experiments" OR "Behavioral Intervention" OR "Behavioral Interventions" OR "Behavioral Modification" OR "Behavioral Modifications" OR "Behavioral Program" OR "Behavioral Programme" OR "Behavioral Programmes" OR "Behavioral Programs" OR "Behavioral Promotion" OR "Behavioral Promotions" OR "Behavioral Trial" OR "Behavioral Trials" OR "Behaviors Change" OR "Behaviors Changes" OR "Behaviors experiment" OR "Behaviors experiments" OR "Behaviors Intervention" OR "Behaviors Interventions" OR "Behaviors Modification" OR "Behaviors Modifications" OR "Behaviors Program" OR "Behaviors Programme" OR "Behaviors Programmes" OR "Behaviors Programs" OR "Behaviors Promotion" OR "Behaviors Promotions" OR "Behaviors Trial" OR "Behaviors Trials" OR "Behaviour Change" OR "Behaviour Changes" OR "Behaviour experiment" OR "Behaviour experiments" OR "Behaviour Intervention" OR "Behaviour Interventions" OR "Behaviour Modification" OR "Behaviour Modifications" OR "Behaviour Program" OR "Behaviour Programme" OR "Behaviour Programmes" OR "Behaviour Programs" OR "Behaviour Promotion" OR "Behaviour Promotions" OR "Behaviour Trial" OR "Behaviour Trials" OR "Behavioural Change" OR "Behavioural Changes" OR "Behavioural experiment" OR "Behavioural experiments" OR "Behavioural Intervention" OR "Behavioural Interventions" OR "Behavioural Modification" OR "Behavioural Modifications" OR "Behavioural Program" OR "Behavioural Programme" OR "Behavioural Programmes" OR "Behavioural Programs" OR "Behavioural Promotion" OR "Behavioural Promotions" OR "Behavioural Trial" OR "Behavioural Trials" OR "Behaviours Change" OR "Behaviours Changes" OR "Behaviours experiment" OR "Behaviours experiments" OR "Behaviours Intervention" OR "Behaviours Interventions" OR "Behaviours Modification" OR "Behaviours Modifications" OR "Behaviours Program" OR "Behaviours Programme" OR "Behaviours Programmes" OR "Behaviours Programs" OR "Behaviours Promotion" OR "Behaviours Promotions" OR "Behaviours Trial" OR "Behaviours Trials") OR MA("behavior change" OR "behavior changes" OR "behavior chang*" OR "behavioral change" OR "behavioral changes" OR "behavioral chang*" OR "behaviour change" OR "behaviour changes" OR "behaviour chang*" OR "behavioural change" OR "behavioural changes" OR "behavioural chang*" OR "behavior change interventions" OR "behavior change intervention" OR "behaviour change interventions" OR "behaviour change intervention" OR "behavioral change interventions" OR "behavioral change intervention" OR "behavioural change interventions" OR "behavioural change intervention" OR "behavior change techniques" OR "behavior change technique" OR "behaviour change techniques" OR "behaviour change technique" OR "behavioral change techniques" OR "behavioral change technique" OR "behavioural change techniques" OR "behavioural change technique" OR "Behavior Change" OR "Behavior Changes" OR "Behavior experiment" OR "Behavior experiments" OR "Behavior Intervention" OR "Behavior Interventions" OR "Behavior Modification" OR "Behavior Modifications" OR "Behavior Program" OR "Behavior Programme" OR "Behavior Programmes" OR "Behavior Programs" OR "Behavior Promotion" OR "Behavior Promotions" OR "Behavior Trial" OR "Behavior Trials" OR "Behavioral Change" OR "Behavioral Changes" OR "Behavioral experiment" OR "Behavioral experiments" OR "Behavioral Intervention" OR "Behavioral Interventions" OR "Behavioral Modification" OR "Behavioral Modifications" OR "Behavioral Program" OR "Behavioral Programme" OR "Behavioral Programmes" OR "Behavioral Programs" OR "Behavioral Promotion" OR "Behavioral Promotions" OR "Behavioral Trial" OR "Behavioral Trials" OR "Behaviors Change" OR "Behaviors Changes" OR "Behaviors experiment" OR "Behaviors experiments" OR "Behaviors Intervention" OR "Behaviors Interventions" OR "Behaviors Modification" OR "Behaviors Modifications" OR "Behaviors Program" OR "Behaviors Programme" OR "Behaviors Programmes" OR "Behaviors Programs" OR "Behaviors Promotion" OR "Behaviors Promotions" OR "Behaviors Trial" OR "Behaviors Trials" OR "Behaviour Change" OR "Behaviour Changes" OR "Behaviour experiment" OR "Behaviour experiments" OR "Behaviour Intervention" OR "Behaviour Interventions" OR "Behaviour Modification" OR "Behaviour Modifications" OR "Behaviour Program" OR "Behaviour Programme" OR "Behaviour Programmes" OR "Behaviour Programs" OR "Behaviour Promotion" OR "Behaviour Promotions" OR "Behaviour Trial" OR "Behaviour Trials" OR "Behavioural Change" OR "Behavioural Changes" OR "Behavioural experiment" OR "Behavioural experiments" OR "Behavioural Intervention" OR "Behavioural Interventions" OR "Behavioural Modification" OR "Behavioural Modifications" OR "Behavioural Program" OR "Behavioural Programme" OR "Behavioural Programmes" OR "Behavioural Programs" OR "Behavioural Promotion" OR "Behavioural Promotions" OR "Behavioural Trial" OR "Behavioural Trials" OR "Behaviours Change" OR "Behaviours Changes" OR "Behaviours experiment" OR "Behaviours experiments" OR "Behaviours Intervention" OR "Behaviours Interventions" OR "Behaviours Modification" OR "Behaviours Modifications" OR "Behaviours Program" OR "Behaviours Programme" OR "Behaviours Programmes" OR "Behaviours Programs" OR "Behaviours Promotion" OR "Behaviours Promotions" OR "Behaviours Trial" OR "Behaviours Trials") OR AB("behavior change" OR "behavior changes" OR "behavior chang*" OR "behavioral change" OR "behavioral changes" OR "behavioral chang*" OR "behaviour change" OR "behaviour changes" OR "behaviour chang*" OR "behavioural change" OR "behavioural changes" OR "behavioural chang*" OR "behavior change interventions" OR "behavior change intervention" OR "behaviour change interventions" OR "behaviour change intervention" OR "behavioral change interventions" OR "behavioral change intervention" OR "behavioural change interventions" OR "behavioural change intervention" OR "behavior change techniques" OR "behavior change technique" OR "behaviour change techniques" OR "behaviour change technique" OR "behavioral change techniques" OR "behavioral change technique" OR "behavioural change techniques" OR "behavioural change technique" OR "Behavior Change" OR "Behavior Changes" OR "Behavior experiment" OR "Behavior experiments" OR "Behavior Intervention" OR "Behavior Interventions" OR "Behavior Modification" OR "Behavior Modifications" OR "Behavior Program" OR "Behavior Programme" OR "Behavior Programmes" OR "Behavior Programs" OR "Behavior Promotion" OR "Behavior Promotions" OR "Behavior Trial" OR "Behavior Trials" OR "Behavioral Change" OR "Behavioral Changes" OR "Behavioral experiment" OR "Behavioral experiments" OR "Behavioral Intervention" OR "Behavioral Interventions" OR "Behavioral Modification" OR "Behavioral Modifications" OR "Behavioral Program" OR "Behavioral Programme" OR "Behavioral Programmes" OR "Behavioral Programs" OR "Behavioral Promotion" OR "Behavioral Promotions" OR "Behavioral Trial" OR "Behavioral Trials" OR "Behaviors Change" OR "Behaviors Changes" OR "Behaviors experiment" OR "Behaviors experiments" OR "Behaviors Intervention" OR "Behaviors Interventions" OR "Behaviors Modification" OR "Behaviors Modifications" OR "Behaviors Program" OR "Behaviors Programme" OR "Behaviors Programmes" OR "Behaviors Programs" OR "Behaviors Promotion" OR "Behaviors Promotions" OR "Behaviors Trial" OR "Behaviors Trials" OR "Behaviour Change" OR "Behaviour Changes" OR "Behaviour experiment" OR "Behaviour experiments" OR "Behaviour Intervention" OR "Behaviour Interventions" OR "Behaviour Modification" OR "Behaviour Modifications" OR "Behaviour Program" OR "Behaviour Programme" OR "Behaviour Programmes" OR "Behaviour Programs" OR "Behaviour Promotion" OR "Behaviour Promotions" OR "Behaviour Trial" OR "Behaviour Trials" OR "Behavioural Change" OR "Behavioural Changes" OR "Behavioural experiment" OR "Behavioural experiments" OR "Behavioural Intervention" OR "Behavioural Interventions" OR "Behavioural Modification" OR "Behavioural Modifications" OR "Behavioural Program" OR "Behavioural Programme" OR "Behavioural Programmes" OR "Behavioural Programs" OR "Behavioural Promotion" OR "Behavioural Promotions" OR "Behavioural Trial" OR "Behavioural Trials" OR "Behaviours Change" OR "Behaviours Changes" OR "Behaviours experiment" OR "Behaviours experiments" OR "Behaviours Intervention" OR "Behaviours Interventions" OR "Behaviours Modification" OR "Behaviours Modifications" OR "Behaviours Program" OR "Behaviours Programme" OR "Behaviours Programmes" OR "Behaviours Programs" OR "Behaviours Promotion" OR "Behaviours Promotions" OR "Behaviours Trial" OR "Behaviours Trials") OR SU("behavior change" OR "behavior changes" OR "behavior chang*" OR "behavioral change" OR "behavioral changes" OR "behavioral chang*" OR "behaviour change" OR "behaviour changes" OR "behaviour chang*" OR "behavioural change" OR "behavioural changes" OR "behavioural chang*" OR "behavior change interventions" OR "behavior change intervention" OR "behaviour change interventions" OR "behaviour change intervention" OR "behavioral change interventions" OR "behavioral change intervention" OR "behavioural change interventions" OR "behavioural change intervention" OR "behavior change techniques" OR "behavior change technique" OR "behaviour change techniques" OR "behaviour change technique" OR "behavioral change techniques" OR "behavioral change technique" OR "behavioural change techniques" OR "behavioural change technique" OR "Behavior Change" OR "Behavior Changes" OR "Behavior experiment" OR "Behavior experiments" OR "Behavior Intervention" OR "Behavior Interventions" OR "Behavior Modification" OR "Behavior Modifications" OR "Behavior Program" OR "Behavior Programme" OR "Behavior Programmes" OR "Behavior Programs" OR "Behavior Promotion" OR "Behavior Promotions" OR "Behavior Trial" OR "Behavior Trials" OR "Behavioral Change" OR "Behavioral Changes" OR "Behavioral experiment" OR "Behavioral experiments" OR "Behavioral Intervention" OR "Behavioral Interventions" OR "Behavioral Modification" OR "Behavioral Modifications" OR "Behavioral Program" OR "Behavioral Programme" OR "Behavioral Programmes" OR "Behavioral Programs" OR "Behavioral Promotion" OR "Behavioral Promotions" OR "Behavioral Trial" OR "Behavioral Trials" OR "Behaviors Change" OR "Behaviors Changes" OR "Behaviors experiment" OR "Behaviors experiments" OR "Behaviors Intervention" OR "Behaviors Interventions" OR "Behaviors Modification" OR "Behaviors Modifications" OR "Behaviors Program" OR "Behaviors Programme" OR "Behaviors Programmes" OR "Behaviors Programs" OR "Behaviors Promotion" OR "Behaviors Promotions" OR "Behaviors Trial" OR "Behaviors Trials" OR "Behaviour Change" OR "Behaviour Changes" OR "Behaviour experiment" OR "Behaviour experiments" OR "Behaviour Intervention" OR "Behaviour Interventions" OR "Behaviour Modification" OR "Behaviour Modifications" OR "Behaviour Program" OR "Behaviour Programme" OR "Behaviour Programmes" OR "Behaviour Programs" OR "Behaviour Promotion" OR "Behaviour Promotions" OR "Behaviour Trial" OR "Behaviour Trials" OR "Behavioural Change" OR "Behavioural Changes" OR "Behavioural experiment" OR "Behavioural experiments" OR "Behavioural Intervention" OR "Behavioural Interventions" OR "Behavioural Modification" OR "Behavioural Modifications" OR "Behavioural Program" OR "Behavioural Programme" OR "Behavioural Programmes" OR "Behavioural Programs" OR "Behavioural Promotion" OR "Behavioural Promotions" OR "Behavioural Trial" OR "Behavioural Trials" OR "Behaviours Change" OR "Behaviours Changes" OR "Behaviours experiment" OR "Behaviours experiments" OR "Behaviours Intervention" OR "Behaviours Interventions" OR "Behaviours Modification" OR "Behaviours Modifications" OR "Behaviours Program" OR "Behaviours Programme" OR "Behaviours Programmes" OR "Behaviours Programs" OR "Behaviours Promotion" OR "Behaviours Promotions" OR "Behaviours Trial" OR "Behaviours Trials") OR TI(("promotion" OR "promote" OR "promoting") AND ("intervention" OR "interventions")) OR TI(("change" OR "changes" OR "chang*" OR "experiment" OR "experiments" OR "Intervention" OR "Interventions" OR "Modification" OR "Modifications" OR "Program" OR "Programme" OR "Programmes" OR "Programs" OR "Promotion" OR "Promotions" OR "Trial" OR "Trials" OR "influence" OR "influences" OR "influencing" OR "influenced" OR "influenc*") AND ("behavior" OR "behaviors" OR "behavioral" OR "behavior*" OR "behaviour" OR "behaviours" OR "behavioural" OR "behaviour*") AND ("Exercise" OR "Physical activity" OR "Physical activities" OR "Exercise" OR "Exercises" OR "Exercising" OR "Exercis*" OR "Strength training" OR "Aerobic" OR "Aerobics" OR "Resistance training" OR "Walking" OR "Endurance Training" OR "Exergaming" OR "Gymnastics" OR "Interval Training" OR "Jogging" OR "Nordic Walking" OR "Physical Conditioning" OR "Running" OR "Stair Climbing" OR "Swimming" OR "Sitting" OR "Sedentary Behavior" OR "Sedentary behaviour" OR "Sedentary behavior" OR "Activity" OR "activities" OR "Inactivity" OR "inactivities" OR "Inactivit*" OR "Diet" OR "diet" OR "diets" OR "diet*" OR "nutrition" OR "nutritional" OR "nutrition*" OR "Eating" OR "eat" OR "eating" OR "consumption" OR "food intake" OR "food pattern" OR "food habit" OR "food patterns" OR "food habits" OR "food intake" OR "Food" OR "food" OR "foods" OR "food-related" OR "vegetable" OR "vegetables" OR "fruit" OR "fruits" OR "wholegrain" OR "wholegrains" OR "legume*" OR "nut" OR "nuts" OR "dairy" OR "fish" OR "tea" OR "fat" OR "fats" OR "oil" OR "oils" OR "coffee" OR "red meat" OR "processed meat" OR "Food and Beverages" OR "Beverages" OR "sweetened beverage*" OR "juice*" OR "Drinking Behavior" OR "Drinking Behavior" OR "Drinking Behaviour" OR "Alcohol Drinking" OR "Ethanol" OR "Ethanol" OR "alcohol" OR "Alcohol consumption" OR "Alcohol drinking" OR "alcohol use" OR "Energy Intake" OR "Weight Gain" OR "Weight gain" OR "gain weight" OR "gaining weight" OR "poor diet" OR "poor diets" OR "poor dietary" OR "healthy eating" OR "Smoking" OR "Smoking" OR "Smoking Cessation" OR "Smoking Reduction" OR "Tobacco Use Cessation" OR "Smoking cessation" OR "Tobacco" OR "Tobacco Products" OR "Tobacco use" OR "Tobacco use")) OR (AB(("change" OR "changes" OR "chang*" OR "experiment" OR "experiments" OR "Intervention" OR "Interventions" OR "Modification" OR "Modifications" OR "Program" OR "Programme" OR "Programmes" OR "Programs" OR "Promotion" OR "Promotions" OR "Trial" OR "Trials" OR "influence" OR "influences" OR "influencing" OR "influenced" OR "influenc*") N2 ("behavior" OR "behaviors" OR "behavioral" OR "behavior*" OR "behaviour" OR "behaviours" OR "behavioural" OR "behaviour*") N2 ("Exercise" OR "Physical activity" OR "Physical activities" OR "Exercise" OR "Exercises" OR "Exercising" OR "Exercis*" OR "Strength training" OR "Aerobic" OR "Aerobics" OR "Resistance training" OR "Walking" OR "Endurance Training" OR "Exergaming" OR "Gymnastics" OR "Interval Training" OR "Jogging" OR "Nordic Walking" OR "Physical Conditioning" OR "Running" OR "Stair Climbing" OR "Swimming" OR "Sitting" OR "Sedentary Behavior" OR "Sedentary behaviour" OR "Sedentary behavior" OR "Activity" OR "activities" OR "Inactivity" OR "inactivities" OR "Inactivit*" OR "Diet" OR "diet" OR "diets" OR "diet*" OR "nutrition" OR "nutritional" OR "nutrition*" OR "Eating" OR "eat" OR "eating" OR "consumption" OR "food intake" OR "food pattern" OR "food habit" OR "food patterns" OR "food habits" OR "food intake" OR "Food" OR "food" OR "foods" OR "food-related" OR "vegetable" OR "vegetables" OR "fruit" OR "fruits" OR "wholegrain" OR "wholegrains" OR "legume*" OR "nut" OR "nuts" OR "dairy" OR "fish" OR "tea" OR "fat" OR "fats" OR "oil" OR "oils" OR "coffee" OR "red meat" OR "processed meat" OR "Food and Beverages" OR "Beverages" OR "sweetened beverage*" OR "juice*" OR "Drinking Behavior" OR "Drinking Behavior" OR "Drinking Behaviour" OR "Alcohol Drinking" OR "Ethanol" OR "Ethanol" OR "alcohol" OR "Alcohol consumption" OR "Alcohol drinking" OR "alcohol use" OR "Energy Intake" OR "Weight Gain" OR "Weight gain" OR "gain weight" OR "gaining weight" OR "poor diet" OR "poor diets" OR "poor dietary" OR "healthy eating" OR "Smoking" OR "Smoking" OR "Smoking Cessation" OR "Smoking Reduction" OR "Tobacco Use Cessation" OR "Smoking cessation" OR "Tobacco" OR "Tobacco Products" OR "Tobacco use" OR "Tobacco use")))) NOT TI("Telemedicine" OR "e-health" OR "m-health" OR "ehealth" OR "mhealth" OR "telehealth" OR "schoolbased" OR "school based" OR "digital intervention" OR "digital interventions" OR "digital" OR "online" OR "internet" OR "smartphone" OR "smartphones" OR "phone" OR "phones"))

**Academic Search Premier (EbscoHOST)**

AND PY=(2000 OR 2001 OR 2002 OR 2003 OR 2004 OR 2005 OR 2006 OR 2007 OR 2008 OR 2009 OR 2010 OR 2011 OR 2012 OR 2013 OR 2014 OR 2015 OR 2016 OR 2017 OR 2018 OR 2019 OR 2020 OR 2021 OR 2022 OR 2023)

((TI("Economic Status" OR "Educational Status" OR "Employment" OR "Unemployment" OR "Income" OR "Salaries and Fringe Benefits" OR "Occupations" OR "Poverty" OR "Poverty Areas" OR "Social Class" OR "Social Conditions" OR "Economic Status" OR "Educational Status" OR "Employment" OR "Income" OR "Occupation" OR "Occupations" OR "Poverty" OR "Poverty Area" OR "Poverty Areas" OR "Salaries" OR "Salary" OR "Social Class" OR "Social Classes" OR "Social Condition" OR "Social Conditions" OR "Unemployment" OR "socioeconomic position" OR "socioeconomic positions" OR "socioeconomic posit*" OR "socio economic position" OR "socio economic positions" OR "socio economic posit*" OR "social economic position" OR "social economic posit*" OR "socioeconomic status" OR "socio economic status" OR "social economic status" OR "deprived urban area" OR "deprived suburban area" OR "deprived area" OR "blue collar" OR "blue-collar" OR "deprive*" OR "deprived" OR "disadvantaged" OR "education level*" OR "educational level" OR "employment*" OR "income*" OR "job" OR "jobs" OR "low education" OR "low income" OR "low-educat*" OR "low-income" OR "social class*" OR "social disparity" OR "social disparities" OR "social disparit*" OR "social inequalit*" OR "social inequalities" OR "social inequality" OR "social inequit*" OR "social inequities" OR "social inequity" OR "health inequity" OR "health inequality" OR "health inequities" OR "health inequalities" OR "health inequit*" OR "health inequalit*" OR "Healthcare Disparity" OR "Healthcare Disparities" OR "Health care Disparity" OR "Health care Disparities" OR "social position" OR "social standing" OR "social status" OR "social strata" OR "socioeconomic" OR "socio-economic" OR "socio-economic" OR "socioeconomic factor" OR "socioeconomic factors" OR "Socio-economic factors" OR "Socio-economic factor" OR "socio-economic status" OR "socioeconomically" OR "socio-economically" OR "underprivileged" OR "unemployed" OR "working class" OR "working-class " OR "years of education" OR "years of schooling" OR "job status" OR "occupational status" OR "occupation status") OR KW("Economic Status" OR "Educational Status" OR "Employment" OR "Unemployment" OR "Income" OR "Salaries and Fringe Benefits" OR "Occupations" OR "Poverty" OR "Poverty Areas" OR "Social Class" OR "Social Conditions" OR "Economic Status" OR "Educational Status" OR "Employment" OR "Income" OR "Occupation" OR "Occupations" OR "Poverty" OR "Poverty Area" OR "Poverty Areas" OR "Salaries" OR "Salary" OR "Social Class" OR "Social Classes" OR "Social Condition" OR "Social Conditions" OR "Unemployment" OR "socioeconomic position" OR "socioeconomic positions" OR "socioeconomic posit*" OR "socio economic position" OR "socio economic positions" OR "socio economic posit*" OR "social economic position" OR "social economic posit*" OR "socioeconomic status" OR "socio economic status" OR "social economic status" OR "deprived urban area" OR "deprived suburban area" OR "deprived area" OR "blue collar" OR "blue-collar" OR "deprive*" OR "deprived" OR "disadvantaged" OR "education level*" OR "educational level" OR "employment*" OR "income*" OR "job" OR "jobs" OR "low education" OR "low income" OR "low-educat*" OR "low-income" OR "social class*" OR "social disparity" OR "social disparities" OR "social disparit*" OR "social inequalit*" OR "social inequalities" OR "social inequality" OR "social inequit*" OR "social inequities" OR "social inequity" OR "health inequity" OR "health inequality" OR "health inequities" OR "health inequalities" OR "health inequit*" OR "health inequalit*" OR "Healthcare Disparity" OR "Healthcare Disparities" OR "Health care Disparity" OR "Health care Disparities" OR "social position" OR "social standing" OR "social status" OR "social strata" OR "socioeconomic" OR "socio-economic" OR "socio-economic" OR "socioeconomic factor" OR "socioeconomic factors" OR "Socio-economic factors" OR "Socio-economic factor" OR "socio-economic status" OR "socioeconomically" OR "socio-economically" OR "underprivileged" OR "unemployed" OR "working class" OR "working-class " OR "years of education" OR "years of schooling" OR "job status" OR "occupational status" OR "occupation status") OR AB("Economic Status" OR "Educational Status" OR "Employment" OR "Unemployment" OR "Income" OR "Salaries and Fringe Benefits" OR "Occupations" OR "Poverty" OR "Poverty Areas" OR "Social Class" OR "Social Conditions" OR "Economic Status" OR "Educational Status" OR "Employment" OR "Income" OR "Occupation" OR "Occupations" OR "Poverty" OR "Poverty Area" OR "Poverty Areas" OR "Salaries" OR "Salary" OR "Social Class" OR "Social Classes" OR "Social Condition" OR "Social Conditions" OR "Unemployment" OR "socioeconomic position" OR "socioeconomic positions" OR "socioeconomic posit*" OR "socio economic position" OR "socio economic positions" OR "socio economic posit*" OR "social economic position" OR "social economic posit*" OR "socioeconomic status" OR "socio economic status" OR "social economic status" OR "deprived urban area" OR "deprived suburban area" OR "deprived area" OR "blue collar" OR "blue-collar" OR "deprive*" OR "deprived" OR "disadvantaged" OR "education level*" OR "educational level" OR "employment*" OR "income*" OR "job" OR "jobs" OR "low education" OR "low income" OR "low-educat*" OR "low-income" OR "social class*" OR "social disparity" OR "social disparities" OR "social disparit*" OR "social inequalit*" OR "social inequalities" OR "social inequality" OR "social inequit*" OR "social inequities" OR "social inequity" OR "health inequity" OR "health inequality" OR "health inequities" OR "health inequalities" OR "health inequit*" OR "health inequalit*" OR "Healthcare Disparity" OR "Healthcare Disparities" OR "Health care Disparity" OR "Health care Disparities" OR "social position" OR "social standing" OR "social status" OR "social strata" OR "socioeconomic" OR "socio-economic" OR "socio-economic" OR "socioeconomic factor" OR "socioeconomic factors" OR "Socio-economic factors" OR "Socio-economic factor" OR "socio-economic status" OR "socioeconomically" OR "socio-economically" OR "underprivileged" OR "unemployed" OR "working class" OR "working-class " OR "years of education" OR "years of schooling" OR "job status" OR "occupational status" OR "occupation status")) AND (TI("Exercise" OR "Physical activity" OR "Physical activities" OR "Exercise" OR "Exercises" OR "Exercising" OR "Exercis*" OR "Strength training" OR "Aerobic" OR "Aerobics" OR "Resistance training" OR "Walking" OR "Endurance Training" OR "Exergaming" OR "Gymnastics" OR "Interval Training" OR "Jogging" OR "Nordic Walking" OR "Physical Conditioning" OR "Running" OR "Stair Climbing" OR "Swimming" OR "Sitting" OR "Sedentary Behavior" OR "Sedentary behaviour" OR "Sedentary behavior" OR "Activity" OR "activities" OR "Inactivity" OR "inactivities" OR "Inactivit*" OR "Diet" OR "diet" OR "diets" OR "diet*" OR "nutrition" OR "nutritional" OR "nutrition*" OR "Eating" OR "eat" OR "eating" OR "consumption" OR "food intake" OR "food pattern" OR "food habit" OR "food patterns" OR "food habits" OR "food intake" OR "Food" OR "food" OR "foods" OR "food-related" OR "vegetable" OR "vegetables" OR "fruit" OR "fruits" OR "wholegrain" OR "wholegrains" OR "legume*" OR "nut" OR "nuts" OR "dairy" OR "fish" OR "tea" OR "fat" OR "fats" OR "oil" OR "oils" OR "coffee" OR "red meat" OR "processed meat" OR "Food and Beverages" OR "Beverages" OR "sweetened beverage*" OR "juice*" OR "Drinking Behavior" OR "Drinking Behavior" OR "Drinking Behaviour" OR "Alcohol Drinking" OR "Ethanol" OR "Ethanol" OR "alcohol" OR "Alcohol consumption" OR "Alcohol drinking" OR "alcohol use" OR "Energy Intake" OR "Weight Gain" OR "Weight gain" OR "gain weight" OR "gaining weight" OR "poor diet" OR "poor diets" OR "poor dietary" OR "healthy eating" OR "Smoking" OR "Smoking" OR "Smoking Cessation" OR "Smoking Reduction" OR "Tobacco Use Cessation" OR "Smoking cessation" OR "Tobacco" OR "Tobacco Products" OR "Tobacco use" OR "Tobacco use") OR KW("Exercise" OR "Physical activity" OR "Physical activities" OR "Exercise" OR "Exercises" OR "Exercising" OR "Exercis*" OR "Strength training" OR "Aerobic" OR "Aerobics" OR "Resistance training" OR "Walking" OR "Endurance Training" OR "Exergaming" OR "Gymnastics" OR "Interval Training" OR "Jogging" OR "Nordic Walking" OR "Physical Conditioning" OR "Running" OR "Stair Climbing" OR "Swimming" OR "Sitting" OR "Sedentary Behavior" OR "Sedentary behaviour" OR "Sedentary behavior" OR "Activity" OR "activities" OR "Inactivity" OR "inactivities" OR "Inactivit*" OR "Diet" OR "diet" OR "diets" OR "diet*" OR "nutrition" OR "nutritional" OR "nutrition*" OR "Eating" OR "eat" OR "eating" OR "consumption" OR "food intake" OR "food pattern" OR "food habit" OR "food patterns" OR "food habits" OR "food intake" OR "Food" OR "food" OR "foods" OR "food-related" OR "vegetable" OR "vegetables" OR "fruit" OR "fruits" OR "wholegrain" OR "wholegrains" OR "legume*" OR "nut" OR "nuts" OR "dairy" OR "fish" OR "tea" OR "fat" OR "fats" OR "oil" OR "oils" OR "coffee" OR "red meat" OR "processed meat" OR "Food and Beverages" OR "Beverages" OR "sweetened beverage*" OR "juice*" OR "Drinking Behavior" OR "Drinking Behavior" OR "Drinking Behaviour" OR "Alcohol Drinking" OR "Ethanol" OR "Ethanol" OR "alcohol" OR "Alcohol consumption" OR "Alcohol drinking" OR "alcohol use" OR "Energy Intake" OR "Weight Gain" OR "Weight gain" OR "gain weight" OR "gaining weight" OR "poor diet" OR "poor diets" OR "poor dietary" OR "healthy eating" OR "Smoking" OR "Smoking" OR "Smoking Cessation" OR "Smoking Reduction" OR "Tobacco Use Cessation" OR "Smoking cessation" OR "Tobacco" OR "Tobacco Products" OR "Tobacco use" OR "Tobacco use") OR AB("Exercise" OR "Physical activity" OR "Physical activities" OR "Exercise" OR "Exercises" OR "Exercising" OR "Exercis*" OR "Strength training" OR "Aerobic" OR "Aerobics" OR "Resistance training" OR "Walking" OR "Endurance Training" OR "Exergaming" OR "Gymnastics" OR "Interval Training" OR "Jogging" OR "Nordic Walking" OR "Physical Conditioning" OR "Running" OR "Stair Climbing" OR "Swimming" OR "Sitting" OR "Sedentary Behavior" OR "Sedentary behaviour" OR "Sedentary behavior" OR "Activity" OR "activities" OR "Inactivity" OR "inactivities" OR "Inactivit*" OR "Diet" OR "diet" OR "diets" OR "diet*" OR "nutrition" OR "nutritional" OR "nutrition*" OR "Eating" OR "eat" OR "eating" OR "consumption" OR "food intake" OR "food pattern" OR "food habit" OR "food patterns" OR "food habits" OR "food intake" OR "Food" OR "food" OR "foods" OR "food-related" OR "vegetable" OR "vegetables" OR "fruit" OR "fruits" OR "wholegrain" OR "wholegrains" OR "legume*" OR "nut" OR "nuts" OR "dairy" OR "fish" OR "tea" OR "fat" OR "fats" OR "oil" OR "oils" OR "coffee" OR "red meat" OR "processed meat" OR "Food and Beverages" OR "Beverages" OR "sweetened beverage*" OR "juice*" OR "Drinking Behavior" OR "Drinking Behavior" OR "Drinking Behaviour" OR "Alcohol Drinking" OR "Ethanol" OR "Ethanol" OR "alcohol" OR "Alcohol consumption" OR "Alcohol drinking" OR "alcohol use" OR "Energy Intake" OR "Weight Gain" OR "Weight gain" OR "gain weight" OR "gaining weight" OR "poor diet" OR "poor diets" OR "poor dietary" OR "healthy eating" OR "Smoking" OR "Smoking" OR "Smoking Cessation" OR "Smoking Reduction" OR "Tobacco Use Cessation" OR "Smoking cessation" OR "Tobacco" OR "Tobacco Products" OR "Tobacco use" OR "Tobacco use")) AND (TI("behavior change" OR "behavior changes" OR "behavior chang*" OR "behavioral change" OR "behavioral changes" OR "behavioral chang*" OR "behaviour change" OR "behaviour changes" OR "behaviour chang*" OR "behavioural change" OR "behavioural changes" OR "behavioural chang*" OR "behavior change interventions" OR "behavior change intervention" OR "behaviour change interventions" OR "behaviour change intervention" OR "behavioral change interventions" OR "behavioral change intervention" OR "behavioural change interventions" OR "behavioural change intervention" OR "behavior change techniques" OR "behavior change technique" OR "behaviour change techniques" OR "behaviour change technique" OR "behavioral change techniques" OR "behavioral change technique" OR "behavioural change techniques" OR "behavioural change technique" OR "Behavior Change" OR "Behavior Changes" OR "Behavior experiment" OR "Behavior experiments" OR "Behavior Intervention" OR "Behavior Interventions" OR "Behavior Modification" OR "Behavior Modifications" OR "Behavior Program" OR "Behavior Programme" OR "Behavior Programmes" OR "Behavior Programs" OR "Behavior Promotion" OR "Behavior Promotions" OR "Behavior Trial" OR "Behavior Trials" OR "Behavioral Change" OR "Behavioral Changes" OR "Behavioral experiment" OR "Behavioral experiments" OR "Behavioral Intervention" OR "Behavioral Interventions" OR "Behavioral Modification" OR "Behavioral Modifications" OR "Behavioral Program" OR "Behavioral Programme" OR "Behavioral Programmes" OR "Behavioral Programs" OR "Behavioral Promotion" OR "Behavioral Promotions" OR "Behavioral Trial" OR "Behavioral Trials" OR "Behaviors Change" OR "Behaviors Changes" OR "Behaviors experiment" OR "Behaviors experiments" OR "Behaviors Intervention" OR "Behaviors Interventions" OR "Behaviors Modification" OR "Behaviors Modifications" OR "Behaviors Program" OR "Behaviors Programme" OR "Behaviors Programmes" OR "Behaviors Programs" OR "Behaviors Promotion" OR "Behaviors Promotions" OR "Behaviors Trial" OR "Behaviors Trials" OR "Behaviour Change" OR "Behaviour Changes" OR "Behaviour experiment" OR "Behaviour experiments" OR "Behaviour Intervention" OR "Behaviour Interventions" OR "Behaviour Modification" OR "Behaviour Modifications" OR "Behaviour Program" OR "Behaviour Programme" OR "Behaviour Programmes" OR "Behaviour Programs" OR "Behaviour Promotion" OR "Behaviour Promotions" OR "Behaviour Trial" OR "Behaviour Trials" OR "Behavioural Change" OR "Behavioural Changes" OR "Behavioural experiment" OR "Behavioural experiments" OR "Behavioural Intervention" OR "Behavioural Interventions" OR "Behavioural Modification" OR "Behavioural Modifications" OR "Behavioural Program" OR "Behavioural Programme" OR "Behavioural Programmes" OR "Behavioural Programs" OR "Behavioural Promotion" OR "Behavioural Promotions" OR "Behavioural Trial" OR "Behavioural Trials" OR "Behaviours Change" OR "Behaviours Changes" OR "Behaviours experiment" OR "Behaviours experiments" OR "Behaviours Intervention" OR "Behaviours Interventions" OR "Behaviours Modification" OR "Behaviours Modifications" OR "Behaviours Program" OR "Behaviours Programme" OR "Behaviours Programmes" OR "Behaviours Programs" OR "Behaviours Promotion" OR "Behaviours Promotions" OR "Behaviours Trial" OR "Behaviours Trials") OR KW("behavior change" OR "behavior changes" OR "behavior chang*" OR "behavioral change" OR "behavioral changes" OR "behavioral chang*" OR "behaviour change" OR "behaviour changes" OR "behaviour chang*" OR "behavioural change" OR "behavioural changes" OR "behavioural chang*" OR "behavior change interventions" OR "behavior change intervention" OR "behaviour change interventions" OR "behaviour change intervention" OR "behavioral change interventions" OR "behavioral change intervention" OR "behavioural change interventions" OR "behavioural change intervention" OR "behavior change techniques" OR "behavior change technique" OR "behaviour change techniques" OR "behaviour change technique" OR "behavioral change techniques" OR "behavioral change technique" OR "behavioural change techniques" OR "behavioural change technique" OR "Behavior Change" OR "Behavior Changes" OR "Behavior experiment" OR "Behavior experiments" OR "Behavior Intervention" OR "Behavior Interventions" OR "Behavior Modification" OR "Behavior Modifications" OR "Behavior Program" OR "Behavior Programme" OR "Behavior Programmes" OR "Behavior Programs" OR "Behavior Promotion" OR "Behavior Promotions" OR "Behavior Trial" OR "Behavior Trials" OR "Behavioral Change" OR "Behavioral Changes" OR "Behavioral experiment" OR "Behavioral experiments" OR "Behavioral Intervention" OR "Behavioral Interventions" OR "Behavioral Modification" OR "Behavioral Modifications" OR "Behavioral Program" OR "Behavioral Programme" OR "Behavioral Programmes" OR "Behavioral Programs" OR "Behavioral Promotion" OR "Behavioral Promotions" OR "Behavioral Trial" OR "Behavioral Trials" OR "Behaviors Change" OR "Behaviors Changes" OR "Behaviors experiment" OR "Behaviors experiments" OR "Behaviors Intervention" OR "Behaviors Interventions" OR "Behaviors Modification" OR "Behaviors Modifications" OR "Behaviors Program" OR "Behaviors Programme" OR "Behaviors Programmes" OR "Behaviors Programs" OR "Behaviors Promotion" OR "Behaviors Promotions" OR "Behaviors Trial" OR "Behaviors Trials" OR "Behaviour Change" OR "Behaviour Changes" OR "Behaviour experiment" OR "Behaviour experiments" OR "Behaviour Intervention" OR "Behaviour Interventions" OR "Behaviour Modification" OR "Behaviour Modifications" OR "Behaviour Program" OR "Behaviour Programme" OR "Behaviour Programmes" OR "Behaviour Programs" OR "Behaviour Promotion" OR "Behaviour Promotions" OR "Behaviour Trial" OR "Behaviour Trials" OR "Behavioural Change" OR "Behavioural Changes" OR "Behavioural experiment" OR "Behavioural experiments" OR "Behavioural Intervention" OR "Behavioural Interventions" OR "Behavioural Modification" OR "Behavioural Modifications" OR "Behavioural Program" OR "Behavioural Programme" OR "Behavioural Programmes" OR "Behavioural Programs" OR "Behavioural Promotion" OR "Behavioural Promotions" OR "Behavioural Trial" OR "Behavioural Trials" OR "Behaviours Change" OR "Behaviours Changes" OR "Behaviours experiment" OR "Behaviours experiments" OR "Behaviours Intervention" OR "Behaviours Interventions" OR "Behaviours Modification" OR "Behaviours Modifications" OR "Behaviours Program" OR "Behaviours Programme" OR "Behaviours Programmes" OR "Behaviours Programs" OR "Behaviours Promotion" OR "Behaviours Promotions" OR "Behaviours Trial" OR "Behaviours Trials") OR SU("behavior change" OR "behavior changes" OR "behavior chang*" OR "behavioral change" OR "behavioral changes" OR "behavioral chang*" OR "behaviour change" OR "behaviour changes" OR "behaviour chang*" OR "behavioural change" OR "behavioural changes" OR "behavioural chang*" OR "behavior change interventions" OR "behavior change intervention" OR "behaviour change interventions" OR "behaviour change intervention" OR "behavioral change interventions" OR "behavioral change intervention" OR "behavioural change interventions" OR "behavioural change intervention" OR "behavior change techniques" OR "behavior change technique" OR "behaviour change techniques" OR "behaviour change technique" OR "behavioral change techniques" OR "behavioral change technique" OR "behavioural change techniques" OR "behavioural change technique" OR "Behavior Change" OR "Behavior Changes" OR "Behavior experiment" OR "Behavior experiments" OR "Behavior Intervention" OR "Behavior Interventions" OR "Behavior Modification" OR "Behavior Modifications" OR "Behavior Program" OR "Behavior Programme" OR "Behavior Programmes" OR "Behavior Programs" OR "Behavior Promotion" OR "Behavior Promotions" OR "Behavior Trial" OR "Behavior Trials" OR "Behavioral Change" OR "Behavioral Changes" OR "Behavioral experiment" OR "Behavioral experiments" OR "Behavioral Intervention" OR "Behavioral Interventions" OR "Behavioral Modification" OR "Behavioral Modifications" OR "Behavioral Program" OR "Behavioral Programme" OR "Behavioral Programmes" OR "Behavioral Programs" OR "Behavioral Promotion" OR "Behavioral Promotions" OR "Behavioral Trial" OR "Behavioral Trials" OR "Behaviors Change" OR "Behaviors Changes" OR "Behaviors experiment" OR "Behaviors experiments" OR "Behaviors Intervention" OR "Behaviors Interventions" OR "Behaviors Modification" OR "Behaviors Modifications" OR "Behaviors Program" OR "Behaviors Programme" OR "Behaviors Programmes" OR "Behaviors Programs" OR "Behaviors Promotion" OR "Behaviors Promotions" OR "Behaviors Trial" OR "Behaviors Trials" OR "Behaviour Change" OR "Behaviour Changes" OR "Behaviour experiment" OR "Behaviour experiments" OR "Behaviour Intervention" OR "Behaviour Interventions" OR "Behaviour Modification" OR "Behaviour Modifications" OR "Behaviour Program" OR "Behaviour Programme" OR "Behaviour Programmes" OR "Behaviour Programs" OR "Behaviour Promotion" OR "Behaviour Promotions" OR "Behaviour Trial" OR "Behaviour Trials" OR "Behavioural Change" OR "Behavioural Changes" OR "Behavioural experiment" OR "Behavioural experiments" OR "Behavioural Intervention" OR "Behavioural Interventions" OR "Behavioural Modification" OR "Behavioural Modifications" OR "Behavioural Program" OR "Behavioural Programme" OR "Behavioural Programmes" OR "Behavioural Programs" OR "Behavioural Promotion" OR "Behavioural Promotions" OR "Behavioural Trial" OR "Behavioural Trials" OR "Behaviours Change" OR "Behaviours Changes" OR "Behaviours experiment" OR "Behaviours experiments" OR "Behaviours Intervention" OR "Behaviours Interventions" OR "Behaviours Modification" OR "Behaviours Modifications" OR "Behaviours Program" OR "Behaviours Programme" OR "Behaviours Programmes" OR "Behaviours Programs" OR "Behaviours Promotion" OR "Behaviours Promotions" OR "Behaviours Trial" OR "Behaviours Trials") OR TI(("promotion" OR "promote" OR "promoting") AND ("intervention" OR "interventions")) OR TI(("change" OR "changes" OR "chang*" OR "experiment" OR "experiments" OR "Intervention" OR "Interventions" OR "Modification" OR "Modifications" OR "Program" OR "Programme" OR "Programmes" OR "Programs" OR "Promotion" OR "Promotions" OR "Trial" OR "Trials" OR "influence" OR "influences" OR "influencing" OR "influenced" OR "influenc*") AND ("behavior" OR "behaviors" OR "behavioral" OR "behavior*" OR "behaviour" OR "behaviours" OR "behavioural" OR "behaviour*") AND ("Exercise" OR "Physical activity" OR "Physical activities" OR "Exercise" OR "Exercises" OR "Exercising" OR "Exercis*" OR "Strength training" OR "Aerobic" OR "Aerobics" OR "Resistance training" OR "Walking" OR "Endurance Training" OR "Exergaming" OR "Gymnastics" OR "Interval Training" OR "Jogging" OR "Nordic Walking" OR "Physical Conditioning" OR "Running" OR "Stair Climbing" OR "Swimming" OR "Sitting" OR "Sedentary Behavior" OR "Sedentary behaviour" OR "Sedentary behavior" OR "Activity" OR "activities" OR "Inactivity" OR "inactivities" OR "Inactivit*" OR "Diet" OR "diet" OR "diets" OR "diet*" OR "nutrition" OR "nutritional" OR "nutrition*" OR "Eating" OR "eat" OR "eating" OR "consumption" OR "food intake" OR "food pattern" OR "food habit" OR "food patterns" OR "food habits" OR "food intake" OR "Food" OR "food" OR "foods" OR "food-related" OR "vegetable" OR "vegetables" OR "fruit" OR "fruits" OR "wholegrain" OR "wholegrains" OR "legume*" OR "nut" OR "nuts" OR "dairy" OR "fish" OR "tea" OR "fat" OR "fats" OR "oil" OR "oils" OR "coffee" OR "red meat" OR "processed meat" OR "Food and Beverages" OR "Beverages" OR "sweetened beverage*" OR "juice*" OR "Drinking Behavior" OR "Drinking Behavior" OR "Drinking Behaviour" OR "Alcohol Drinking" OR "Ethanol" OR "Ethanol" OR "alcohol" OR "Alcohol consumption" OR "Alcohol drinking" OR "alcohol use" OR "Energy Intake" OR "Weight Gain" OR "Weight gain" OR "gain weight" OR "gaining weight" OR "poor diet" OR "poor diets" OR "poor dietary" OR "healthy eating" OR "Smoking" OR "Smoking" OR "Smoking Cessation" OR "Smoking Reduction" OR "Tobacco Use Cessation" OR "Smoking cessation" OR "Tobacco" OR "Tobacco Products" OR "Tobacco use" OR "Tobacco use")) OR (AB(("change" OR "changes" OR "chang*" OR "experiment" OR "experiments" OR "Intervention" OR "Interventions" OR "Modification" OR "Modifications" OR "Program" OR "Programme" OR "Programmes" OR "Programs" OR "Promotion" OR "Promotions" OR "Trial" OR "Trials" OR "influence" OR "influences" OR "influencing" OR "influenced" OR "influenc*") N2 ("behavior" OR "behaviors" OR "behavioral" OR "behavior*" OR "behaviour" OR "behaviours" OR "behavioural" OR "behaviour*") N2 ("Exercise" OR "Physical activity" OR "Physical activities" OR "Exercise" OR "Exercises" OR "Exercising" OR "Exercis*" OR "Strength training" OR "Aerobic" OR "Aerobics" OR "Resistance training" OR "Walking" OR "Endurance Training" OR "Exergaming" OR "Gymnastics" OR "Interval Training" OR "Jogging" OR "Nordic Walking" OR "Physical Conditioning" OR "Running" OR "Stair Climbing" OR "Swimming" OR "Sitting" OR "Sedentary Behavior" OR "Sedentary behaviour" OR "Sedentary behavior" OR "Activity" OR "activities" OR "Inactivity" OR "inactivities" OR "Inactivit*" OR "Diet" OR "diet" OR "diets" OR "diet*" OR "nutrition" OR "nutritional" OR "nutrition*" OR "Eating" OR "eat" OR "eating" OR "consumption" OR "food intake" OR "food pattern" OR "food habit" OR "food patterns" OR "food habits" OR "food intake" OR "Food" OR "food" OR "foods" OR "food-related" OR "vegetable" OR "vegetables" OR "fruit" OR "fruits" OR "wholegrain" OR "wholegrains" OR "legume*" OR "nut" OR "nuts" OR "dairy" OR "fish" OR "tea" OR "fat" OR "fats" OR "oil" OR "oils" OR "coffee" OR "red meat" OR "processed meat" OR "Food and Beverages" OR "Beverages" OR "sweetened beverage*" OR "juice*" OR "Drinking Behavior" OR "Drinking Behavior" OR "Drinking Behaviour" OR "Alcohol Drinking" OR "Ethanol" OR "Ethanol" OR "alcohol" OR "Alcohol consumption" OR "Alcohol drinking" OR "alcohol use" OR "Energy Intake" OR "Weight Gain" OR "Weight gain" OR "gain weight" OR "gaining weight" OR "poor diet" OR "poor diets" OR "poor dietary" OR "healthy eating" OR "Smoking" OR "Smoking" OR "Smoking Cessation" OR "Smoking Reduction" OR "Tobacco Use Cessation" OR "Smoking cessation" OR "Tobacco" OR "Tobacco Products" OR "Tobacco use" OR "Tobacco use")))) NOT TI("Telemedicine" OR "e-health" OR "m-health" OR "ehealth" OR "mhealth" OR "telehealth" OR "schoolbased" OR "school based" OR "digital intervention" OR "digital interventions" OR "digital" OR "online" OR "internet" OR "smartphone" OR "smartphones" OR "phone" OR "phones"))

**ProQuest Dissertations and Theses Global**

AND PY=(2000 OR 2001 OR 2002 OR 2003 OR 2004 OR 2005 OR 2006 OR 2007 OR 2008 OR 2009 OR 2010 OR 2011 OR 2012 OR 2013 OR 2014 OR 2015 OR 2016 OR 2017 OR 2018 OR 2019 OR 2020 OR 2021 OR 2022 OR 2023)

((TITLE("Economic Status" OR "Educational Status" OR "Employment" OR "Unemployment" OR "Income" OR "Salaries and Fringe Benefits" OR "Occupations" OR "Poverty" OR "Poverty Areas" OR "Social Class" OR "Social Conditions" OR "Economic Status" OR "Educational Status" OR "Employment" OR "Income" OR "Occupation" OR "Occupations" OR "Poverty" OR "Poverty Area" OR "Poverty Areas" OR "Salaries" OR "Salary" OR "Social Class" OR "Social Classes" OR "Social Condition" OR "Social Conditions" OR "Unemployment" OR "socioeconomic position" OR "socioeconomic positions" OR "socioeconomic posit*" OR "socio economic position" OR "socio economic positions" OR "socio economic posit*" OR "social economic position" OR "social economic posit*" OR "socioeconomic status" OR "socio economic status" OR "social economic status" OR "deprived urban area" OR "deprived suburban area" OR "deprived area" OR "blue collar" OR "blue-collar" OR "deprive*" OR "deprived" OR "disadvantaged" OR "education level*" OR "educational level" OR "employment*" OR "income*" OR "job" OR "jobs" OR "low education" OR "low income" OR "low-educat*" OR "low-income" OR "social class*" OR "social disparity" OR "social disparities" OR "social disparit*" OR "social inequalit*" OR "social inequalities" OR "social inequality" OR "social inequit*" OR "social inequities" OR "social inequity" OR "health inequity" OR "health inequality" OR "health inequities" OR "health inequalities" OR "health inequit*" OR "health inequalit*" OR "Healthcare Disparity" OR "Healthcare Disparities" OR "Health care Disparity" OR "Health care Disparities" OR "social position" OR "social standing" OR "social status" OR "social strata" OR "socioeconomic" OR "socio-economic" OR "socio-economic" OR "socioeconomic factor" OR "socioeconomic factors" OR "Socio-economic factors" OR "Socio-economic factor" OR "socio-economic status" OR "socioeconomically" OR "socio-economically" OR "underprivileged" OR "unemployed" OR "working class" OR "working-class " OR "years of education" OR "years of schooling" OR "job status" OR "occupational status" OR "occupation status") OR ABSTRACT("Economic Status" OR "Educational Status" OR "Employment" OR "Unemployment" OR "Income" OR "Salaries and Fringe Benefits" OR "Occupations" OR "Poverty" OR "Poverty Areas" OR "Social Class" OR "Social Conditions" OR "Economic Status" OR "Educational Status" OR "Employment" OR "Income" OR "Occupation" OR "Occupations" OR "Poverty" OR "Poverty Area" OR "Poverty Areas" OR "Salaries" OR "Salary" OR "Social Class" OR "Social Classes" OR "Social Condition" OR "Social Conditions" OR "Unemployment" OR "socioeconomic position" OR "socioeconomic positions" OR "socioeconomic posit*" OR "socio economic position" OR "socio economic positions" OR "socio economic posit*" OR "social economic position" OR "social economic posit*" OR "socioeconomic status" OR "socio economic status" OR "social economic status" OR "deprived urban area" OR "deprived suburban area" OR "deprived area" OR "blue collar" OR "blue-collar" OR "deprive*" OR "deprived" OR "disadvantaged" OR "education level*" OR "educational level" OR "employment*" OR "income*" OR "job" OR "jobs" OR "low education" OR "low income" OR "low-educat*" OR "low-income" OR "social class*" OR "social disparity" OR "social disparities" OR "social disparit*" OR "social inequalit*" OR "social inequalities" OR "social inequality" OR "social inequit*" OR "social inequities" OR "social inequity" OR "health inequity" OR "health inequality" OR "health inequities" OR "health inequalities" OR "health inequit*" OR "health inequalit*" OR "Healthcare Disparity" OR "Healthcare Disparities" OR "Health care Disparity" OR "Health care Disparities" OR "social position" OR "social standing" OR "social status" OR "social strata" OR "socioeconomic" OR "socio-economic" OR "socio-economic" OR "socioeconomic factor" OR "socioeconomic factors" OR "Socio-economic factors" OR "Socio-economic factor" OR "socio-economic status" OR "socioeconomically" OR "socio-economically" OR "underprivileged" OR "unemployed" OR "working class" OR "working-class " OR "years of education" OR "years of schooling" OR "job status" OR "occupational status" OR "occupation status") OR DISKW("Economic Status" OR "Educational Status" OR "Employment" OR "Unemployment" OR "Income" OR "Salaries and Fringe Benefits" OR "Occupations" OR "Poverty" OR "Poverty Areas" OR "Social Class" OR "Social Conditions" OR "Economic Status" OR "Educational Status" OR "Employment" OR "Income" OR "Occupation" OR "Occupations" OR "Poverty" OR "Poverty Area" OR "Poverty Areas" OR "Salaries" OR "Salary" OR "Social Class" OR "Social Classes" OR "Social Condition" OR "Social Conditions" OR "Unemployment" OR "socioeconomic position" OR "socioeconomic positions" OR "socioeconomic posit*" OR "socio economic position" OR "socio economic positions" OR "socio economic posit*" OR "social economic position" OR "social economic posit*" OR "socioeconomic status" OR "socio economic status" OR "social economic status" OR "deprived urban area" OR "deprived suburban area" OR "deprived area" OR "blue collar" OR "blue-collar" OR "deprive*" OR "deprived" OR "disadvantaged" OR "education level*" OR "educational level" OR "employment*" OR "income*" OR "job" OR "jobs" OR "low education" OR "low income" OR "low-educat*" OR "low-income" OR "social class*" OR "social disparity" OR "social disparities" OR "social disparit*" OR "social inequalit*" OR "social inequalities" OR "social inequality" OR "social inequit*" OR "social inequities" OR "social inequity" OR "health inequity" OR "health inequality" OR "health inequities" OR "health inequalities" OR "health inequit*" OR "health inequalit*" OR "Healthcare Disparity" OR "Healthcare Disparities" OR "Health care Disparity" OR "Health care Disparities" OR "social position" OR "social standing" OR "social status" OR "social strata" OR "socioeconomic" OR "socio-economic" OR "socio-economic" OR "socioeconomic factor" OR "socioeconomic factors" OR "Socio-economic factors" OR "Socio-economic factor" OR "socio-economic status" OR "socioeconomically" OR "socio-economically" OR "underprivileged" OR "unemployed" OR "working class" OR "working-class " OR "years of education" OR "years of schooling" OR "job status" OR "occupational status" OR "occupation status")) AND (TITLE("Exercise" OR "Physical activity" OR "Physical activities" OR "Exercise" OR "Exercises" OR "Exercising" OR "Exercis*" OR "Strength training" OR "Aerobic" OR "Aerobics" OR "Resistance training" OR "Walking" OR "Endurance Training" OR "Exergaming" OR "Gymnastics" OR "Interval Training" OR "Jogging" OR "Nordic Walking" OR "Physical Conditioning" OR "Running" OR "Stair Climbing" OR "Swimming" OR "Sitting" OR "Sedentary Behavior" OR "Sedentary behaviour" OR "Sedentary behavior" OR "Activity" OR "activities" OR "Inactivity" OR "inactivities" OR "Inactivit*" OR "Diet" OR "diet" OR "diets" OR "diet*" OR "nutrition" OR "nutritional" OR "nutrition*" OR "Eating" OR "eat" OR "eating" OR "consumption" OR "food intake" OR "food pattern" OR "food habit" OR "food patterns" OR "food habits" OR "food intake" OR "Food" OR "food" OR "foods" OR "food-related" OR "vegetable" OR "vegetables" OR "fruit" OR "fruits" OR "wholegrain" OR "wholegrains" OR "legume*" OR "nut" OR "nuts" OR "dairy" OR "fish" OR "tea" OR "fat" OR "fats" OR "oil" OR "oils" OR "coffee" OR "red meat" OR "processed meat" OR "Food and Beverages" OR "Beverages" OR "sweetened beverage*" OR "juice*" OR "Drinking Behavior" OR "Drinking Behavior" OR "Drinking Behaviour" OR "Alcohol Drinking" OR "Ethanol" OR "Ethanol" OR "alcohol" OR "Alcohol consumption" OR "Alcohol drinking" OR "alcohol use" OR "Energy Intake" OR "Weight Gain" OR "Weight gain" OR "gain weight" OR "gaining weight" OR "poor diet" OR "poor diets" OR "poor dietary" OR "healthy eating" OR "Smoking" OR "Smoking" OR "Smoking Cessation" OR "Smoking Reduction" OR "Tobacco Use Cessation" OR "Smoking cessation" OR "Tobacco" OR "Tobacco Products" OR "Tobacco use" OR "Tobacco use") OR ABSTRACT("Exercise" OR "Physical activity" OR "Physical activities" OR "Exercise" OR "Exercises" OR "Exercising" OR "Exercis*" OR "Strength training" OR "Aerobic" OR "Aerobics" OR "Resistance training" OR "Walking" OR "Endurance Training" OR "Exergaming" OR "Gymnastics" OR "Interval Training" OR "Jogging" OR "Nordic Walking" OR "Physical Conditioning" OR "Running" OR "Stair Climbing" OR "Swimming" OR "Sitting" OR "Sedentary Behavior" OR "Sedentary behaviour" OR "Sedentary behavior" OR "Activity" OR "activities" OR "Inactivity" OR "inactivities" OR "Inactivit*" OR "Diet" OR "diet" OR "diets" OR "diet*" OR "nutrition" OR "nutritional" OR "nutrition*" OR "Eating" OR "eat" OR "eating" OR "consumption" OR "food intake" OR "food pattern" OR "food habit" OR "food patterns" OR "food habits" OR "food intake" OR "Food" OR "food" OR "foods" OR "food-related" OR "vegetable" OR "vegetables" OR "fruit" OR "fruits" OR "wholegrain" OR "wholegrains" OR "legume*" OR "nut" OR "nuts" OR "dairy" OR "fish" OR "tea" OR "fat" OR "fats" OR "oil" OR "oils" OR "coffee" OR "red meat" OR "processed meat" OR "Food and Beverages" OR "Beverages" OR "sweetened beverage*" OR "juice*" OR "Drinking Behavior" OR "Drinking Behavior" OR "Drinking Behaviour" OR "Alcohol Drinking" OR "Ethanol" OR "Ethanol" OR "alcohol" OR "Alcohol consumption" OR "Alcohol drinking" OR "alcohol use" OR "Energy Intake" OR "Weight Gain" OR "Weight gain" OR "gain weight" OR "gaining weight" OR "poor diet" OR "poor diets" OR "poor dietary" OR "healthy eating" OR "Smoking" OR "Smoking" OR "Smoking Cessation" OR "Smoking Reduction" OR "Tobacco Use Cessation" OR "Smoking cessation" OR "Tobacco" OR "Tobacco Products" OR "Tobacco use" OR "Tobacco use") OR DISKW("Exercise" OR "Physical activity" OR "Physical activities" OR "Exercise" OR "Exercises" OR "Exercising" OR "Exercis*" OR "Strength training" OR "Aerobic" OR "Aerobics" OR "Resistance training" OR "Walking" OR "Endurance Training" OR "Exergaming" OR "Gymnastics" OR "Interval Training" OR "Jogging" OR "Nordic Walking" OR "Physical Conditioning" OR "Running" OR "Stair Climbing" OR "Swimming" OR "Sitting" OR "Sedentary Behavior" OR "Sedentary behaviour" OR "Sedentary behavior" OR "Activity" OR "activities" OR "Inactivity" OR "inactivities" OR "Inactivit*" OR "Diet" OR "diet" OR "diets" OR "diet*" OR "nutrition" OR "nutritional" OR "nutrition*" OR "Eating" OR "eat" OR "eating" OR "consumption" OR "food intake" OR "food pattern" OR "food habit" OR "food patterns" OR "food habits" OR "food intake" OR "Food" OR "food" OR "foods" OR "food-related" OR "vegetable" OR "vegetables" OR "fruit" OR "fruits" OR "wholegrain" OR "wholegrains" OR "legume*" OR "nut" OR "nuts" OR "dairy" OR "fish" OR "tea" OR "fat" OR "fats" OR "oil" OR "oils" OR "coffee" OR "red meat" OR "processed meat" OR "Food and Beverages" OR "Beverages" OR "sweetened beverage*" OR "juice*" OR "Drinking Behavior" OR "Drinking Behavior" OR "Drinking Behaviour" OR "Alcohol Drinking" OR "Ethanol" OR "Ethanol" OR "alcohol" OR "Alcohol consumption" OR "Alcohol drinking" OR "alcohol use" OR "Energy Intake" OR "Weight Gain" OR "Weight gain" OR "gain weight" OR "gaining weight" OR "poor diet" OR "poor diets" OR "poor dietary" OR "healthy eating" OR "Smoking" OR "Smoking" OR "Smoking Cessation" OR "Smoking Reduction" OR "Tobacco Use Cessation" OR "Smoking cessation" OR "Tobacco" OR "Tobacco Products" OR "Tobacco use" OR "Tobacco use")) AND (TITLE("behavior change" OR "behavior changes" OR "behavior chang*" OR "behavioral change" OR "behavioral changes" OR "behavioral chang*" OR "behaviour change" OR "behaviour changes" OR "behaviour chang*" OR "behavioural change" OR "behavioural changes" OR "behavioural chang*" OR "behavior change interventions" OR "behavior change intervention" OR "behaviour change interventions" OR "behaviour change intervention" OR "behavioral change interventions" OR "behavioral change intervention" OR "behavioural change interventions" OR "behavioural change intervention" OR "behavior change techniques" OR "behavior change technique" OR "behaviour change techniques" OR "behaviour change technique" OR "behavioral change techniques" OR "behavioral change technique" OR "behavioural change techniques" OR "behavioural change technique" OR "Behavior Change" OR "Behavior Changes" OR "Behavior experiment" OR "Behavior experiments" OR "Behavior Intervention" OR "Behavior Interventions" OR "Behavior Modification" OR "Behavior Modifications" OR "Behavior Program" OR "Behavior Programme" OR "Behavior Programmes" OR "Behavior Programs" OR "Behavior Promotion" OR "Behavior Promotions" OR "Behavior Trial" OR "Behavior Trials" OR "Behavioral Change" OR "Behavioral Changes" OR "Behavioral experiment" OR "Behavioral experiments" OR "Behavioral Intervention" OR "Behavioral Interventions" OR "Behavioral Modification" OR "Behavioral Modifications" OR "Behavioral Program" OR "Behavioral Programme" OR "Behavioral Programmes" OR "Behavioral Programs" OR "Behavioral Promotion" OR "Behavioral Promotions" OR "Behavioral Trial" OR "Behavioral Trials" OR "Behaviors Change" OR "Behaviors Changes" OR "Behaviors experiment" OR "Behaviors experiments" OR "Behaviors Intervention" OR "Behaviors Interventions" OR "Behaviors Modification" OR "Behaviors Modifications" OR "Behaviors Program" OR "Behaviors Programme" OR "Behaviors Programmes" OR "Behaviors Programs" OR "Behaviors Promotion" OR "Behaviors Promotions" OR "Behaviors Trial" OR "Behaviors Trials" OR "Behaviour Change" OR "Behaviour Changes" OR "Behaviour experiment" OR "Behaviour experiments" OR "Behaviour Intervention" OR "Behaviour Interventions" OR "Behaviour Modification" OR "Behaviour Modifications" OR "Behaviour Program" OR "Behaviour Programme" OR "Behaviour Programmes" OR "Behaviour Programs" OR "Behaviour Promotion" OR "Behaviour Promotions" OR "Behaviour Trial" OR "Behaviour Trials" OR "Behavioural Change" OR "Behavioural Changes" OR "Behavioural experiment" OR "Behavioural experiments" OR "Behavioural Intervention" OR "Behavioural Interventions" OR "Behavioural Modification" OR "Behavioural Modifications" OR "Behavioural Program" OR "Behavioural Programme" OR "Behavioural Programmes" OR "Behavioural Programs" OR "Behavioural Promotion" OR "Behavioural Promotions" OR "Behavioural Trial" OR "Behavioural Trials" OR "Behaviours Change" OR "Behaviours Changes" OR "Behaviours experiment" OR "Behaviours experiments" OR "Behaviours Intervention" OR "Behaviours Interventions" OR "Behaviours Modification" OR "Behaviours Modifications" OR "Behaviours Program" OR "Behaviours Programme" OR "Behaviours Programmes" OR "Behaviours Programs" OR "Behaviours Promotion" OR "Behaviours Promotions" OR "Behaviours Trial" OR "Behaviours Trials") OR DISKW("behavior change" OR "behavior changes" OR "behavior chang*" OR "behavioral change" OR "behavioral changes" OR "behavioral chang*" OR "behaviour change" OR "behaviour changes" OR "behaviour chang*" OR "behavioural change" OR "behavioural changes" OR "behavioural chang*" OR "behavior change interventions" OR "behavior change intervention" OR "behaviour change interventions" OR "behaviour change intervention" OR "behavioral change interventions" OR "behavioral change intervention" OR "behavioural change interventions" OR "behavioural change intervention" OR "behavior change techniques" OR "behavior change technique" OR "behaviour change techniques" OR "behaviour change technique" OR "behavioral change techniques" OR "behavioral change technique" OR "behavioural change techniques" OR "behavioural change technique" OR "Behavior Change" OR "Behavior Changes" OR "Behavior experiment" OR "Behavior experiments" OR "Behavior Intervention" OR "Behavior Interventions" OR "Behavior Modification" OR "Behavior Modifications" OR "Behavior Program" OR "Behavior Programme" OR "Behavior Programmes" OR "Behavior Programs" OR "Behavior Promotion" OR "Behavior Promotions" OR "Behavior Trial" OR "Behavior Trials" OR "Behavioral Change" OR "Behavioral Changes" OR "Behavioral experiment" OR "Behavioral experiments" OR "Behavioral Intervention" OR "Behavioral Interventions" OR "Behavioral Modification" OR "Behavioral Modifications" OR "Behavioral Program" OR "Behavioral Programme" OR "Behavioral Programmes" OR "Behavioral Programs" OR "Behavioral Promotion" OR "Behavioral Promotions" OR "Behavioral Trial" OR "Behavioral Trials" OR "Behaviors Change" OR "Behaviors Changes" OR "Behaviors experiment" OR "Behaviors experiments" OR "Behaviors Intervention" OR "Behaviors Interventions" OR "Behaviors Modification" OR "Behaviors Modifications" OR "Behaviors Program" OR "Behaviors Programme" OR "Behaviors Programmes" OR "Behaviors Programs" OR "Behaviors Promotion" OR "Behaviors Promotions" OR "Behaviors Trial" OR "Behaviors Trials" OR "Behaviour Change" OR "Behaviour Changes" OR "Behaviour experiment" OR "Behaviour experiments" OR "Behaviour Intervention" OR "Behaviour Interventions" OR "Behaviour Modification" OR "Behaviour Modifications" OR "Behaviour Program" OR "Behaviour Programme" OR "Behaviour Programmes" OR "Behaviour Programs" OR "Behaviour Promotion" OR "Behaviour Promotions" OR "Behaviour Trial" OR "Behaviour Trials" OR "Behavioural Change" OR "Behavioural Changes" OR "Behavioural experiment" OR "Behavioural experiments" OR "Behavioural Intervention" OR "Behavioural Interventions" OR "Behavioural Modification" OR "Behavioural Modifications" OR "Behavioural Program" OR "Behavioural Programme" OR "Behavioural Programmes" OR "Behavioural Programs" OR "Behavioural Promotion" OR "Behavioural Promotions" OR "Behavioural Trial" OR "Behavioural Trials" OR "Behaviours Change" OR "Behaviours Changes" OR "Behaviours experiment" OR "Behaviours experiments" OR "Behaviours Intervention" OR "Behaviours Interventions" OR "Behaviours Modification" OR "Behaviours Modifications" OR "Behaviours Program" OR "Behaviours Programme" OR "Behaviours Programmes" OR "Behaviours Programs" OR "Behaviours Promotion" OR "Behaviours Promotions" OR "Behaviours Trial" OR "Behaviours Trials")) NOT TITLE("Telemedicine" OR "e-health" OR "m-health" OR "ehealth" OR "mhealth" OR "telehealth" OR "schoolbased" OR "school based" OR "digital intervention" OR "digital interventions" OR "digital" OR "online" OR "internet" OR "smartphone" OR "smartphones" OR "phone" OR "phones")) OR ((TITLE("Economic Status" OR "Educational Status" OR "Employment" OR "Unemployment" OR "Income" OR "Salaries and Fringe Benefits" OR "Occupations" OR "Poverty" OR "Poverty Areas" OR "Social Class" OR "Social Conditions" OR "Economic Status" OR "Educational Status" OR "Employment" OR "Income" OR "Occupation" OR "Occupations" OR "Poverty" OR "Poverty Area" OR "Poverty Areas" OR "Salaries" OR "Salary" OR "Social Class" OR "Social Classes" OR "Social Condition" OR "Social Conditions" OR "Unemployment" OR "socioeconomic position" OR "socioeconomic positions" OR "socioeconomic posit*" OR "socio economic position" OR "socio economic positions" OR "socio economic posit*" OR "social economic position" OR "social economic posit*" OR "socioeconomic status" OR "socio economic status" OR "social economic status" OR "deprived urban area" OR "deprived suburban area" OR "deprived area" OR "blue collar" OR "blue-collar" OR "deprive*" OR "deprived" OR "disadvantaged" OR "education level*" OR "educational level" OR "employment*" OR "income*" OR "job" OR "jobs" OR "low education" OR "low income" OR "low-educat*" OR "low-income" OR "social class*" OR "social disparity" OR "social disparities" OR "social disparit*" OR "social inequalit*" OR "social inequalities" OR "social inequality" OR "social inequit*" OR "social inequities" OR "social inequity" OR "health inequity" OR "health inequality" OR "health inequities" OR "health inequalities" OR "health inequit*" OR "health inequalit*" OR "Healthcare Disparity" OR "Healthcare Disparities" OR "Health care Disparity" OR "Health care Disparities" OR "social position" OR "social standing" OR "social status" OR "social strata" OR "socioeconomic" OR "socio-economic" OR "socio-economic" OR "socioeconomic factor" OR "socioeconomic factors" OR "Socio-economic factors" OR "Socio-economic factor" OR "socio-economic status" OR "socioeconomically" OR "socio-economically" OR "underprivileged" OR "unemployed" OR "working class" OR "working-class " OR "years of education" OR "years of schooling" OR "job status" OR "occupational status" OR "occupation status") OR ABSTRACT("Economic Status" OR "Educational Status" OR "Employment" OR "Unemployment" OR "Income" OR "Salaries and Fringe Benefits" OR "Occupations" OR "Poverty" OR "Poverty Areas" OR "Social Class" OR "Social Conditions" OR "Economic Status" OR "Educational Status" OR "Employment" OR "Income" OR "Occupation" OR "Occupations" OR "Poverty" OR "Poverty Area" OR "Poverty Areas" OR "Salaries" OR "Salary" OR "Social Class" OR "Social Classes" OR "Social Condition" OR "Social Conditions" OR "Unemployment" OR "socioeconomic position" OR "socioeconomic positions" OR "socioeconomic posit*" OR "socio economic position" OR "socio economic positions" OR "socio economic posit*" OR "social economic position" OR "social economic posit*" OR "socioeconomic status" OR "socio economic status" OR "social economic status" OR "deprived urban area" OR "deprived suburban area" OR "deprived area" OR "blue collar" OR "blue-collar" OR "deprive*" OR "deprived" OR "disadvantaged" OR "education level*" OR "educational level" OR "employment*" OR "income*" OR "job" OR "jobs" OR "low education" OR "low income" OR "low-educat*" OR "low-income" OR "social class*" OR "social disparity" OR "social disparities" OR "social disparit*" OR "social inequalit*" OR "social inequalities" OR "social inequality" OR "social inequit*" OR "social inequities" OR "social inequity" OR "health inequity" OR "health inequality" OR "health inequities" OR "health inequalities" OR "health inequit*" OR "health inequalit*" OR "Healthcare Disparity" OR "Healthcare Disparities" OR "Health care Disparity" OR "Health care Disparities" OR "social position" OR "social standing" OR "social status" OR "social strata" OR "socioeconomic" OR "socio-economic" OR "socio-economic" OR "socioeconomic factor" OR "socioeconomic factors" OR "Socio-economic factors" OR "Socio-economic factor" OR "socio-economic status" OR "socioeconomically" OR "socio-economically" OR "underprivileged" OR "unemployed" OR "working class" OR "working-class " OR "years of education" OR "years of schooling" OR "job status" OR "occupational status" OR "occupation status") OR DISKW("Economic Status" OR "Educational Status" OR "Employment" OR "Unemployment" OR "Income" OR "Salaries and Fringe Benefits" OR "Occupations" OR "Poverty" OR "Poverty Areas" OR "Social Class" OR "Social Conditions" OR "Economic Status" OR "Educational Status" OR "Employment" OR "Income" OR "Occupation" OR "Occupations" OR "Poverty" OR "Poverty Area" OR "Poverty Areas" OR "Salaries" OR "Salary" OR "Social Class" OR "Social Classes" OR "Social Condition" OR "Social Conditions" OR "Unemployment" OR "socioeconomic position" OR "socioeconomic positions" OR "socioeconomic posit*" OR "socio economic position" OR "socio economic positions" OR "socio economic posit*" OR "social economic position" OR "social economic posit*" OR "socioeconomic status" OR "socio economic status" OR "social economic status" OR "deprived urban area" OR "deprived suburban area" OR "deprived area" OR "blue collar" OR "blue-collar" OR "deprive*" OR "deprived" OR "disadvantaged" OR "education level*" OR "educational level" OR "employment*" OR "income*" OR "job" OR "jobs" OR "low education" OR "low income" OR "low-educat*" OR "low-income" OR "social class*" OR "social disparity" OR "social disparities" OR "social disparit*" OR "social inequalit*" OR "social inequalities" OR "social inequality" OR "social inequit*" OR "social inequities" OR "social inequity" OR "health inequity" OR "health inequality" OR "health inequities" OR "health inequalities" OR "health inequit*" OR "health inequalit*" OR "Healthcare Disparity" OR "Healthcare Disparities" OR "Health care Disparity" OR "Health care Disparities" OR "social position" OR "social standing" OR "social status" OR "social strata" OR "socioeconomic" OR "socio-economic" OR "socio-economic" OR "socioeconomic factor" OR "socioeconomic factors" OR "Socio-economic factors" OR "Socio-economic factor" OR "socio-economic status" OR "socioeconomically" OR "socio-economically" OR "underprivileged" OR "unemployed" OR "working class" OR "working-class " OR "years of education" OR "years of schooling" OR "job status" OR "occupational status" OR "occupation status")) AND (TITLE("Exercise" OR "Physical activity" OR "Physical activities" OR "Exercise" OR "Exercises" OR "Exercising" OR "Exercis*" OR "Strength training" OR "Aerobic" OR "Aerobics" OR "Resistance training" OR "Walking" OR "Endurance Training" OR "Exergaming" OR "Gymnastics" OR "Interval Training" OR "Jogging" OR "Nordic Walking" OR "Physical Conditioning" OR "Running" OR "Stair Climbing" OR "Swimming" OR "Sitting" OR "Sedentary Behavior" OR "Sedentary behaviour" OR "Sedentary behavior" OR "Activity" OR "activities" OR "Inactivity" OR "inactivities" OR "Inactivit*" OR "Diet" OR "diet" OR "diets" OR "diet*" OR "nutrition" OR "nutritional" OR "nutrition*" OR "Eating" OR "eat" OR "eating" OR "consumption" OR "food intake" OR "food pattern" OR "food habit" OR "food patterns" OR "food habits" OR "food intake" OR "Food" OR "food" OR "foods" OR "food-related" OR "vegetable" OR "vegetables" OR "fruit" OR "fruits" OR "wholegrain" OR "wholegrains" OR "legume*" OR "nut" OR "nuts" OR "dairy" OR "fish" OR "tea" OR "fat" OR "fats" OR "oil" OR "oils" OR "coffee" OR "red meat" OR "processed meat" OR "Food and Beverages" OR "Beverages" OR "sweetened beverage*" OR "juice*" OR "Drinking Behavior" OR "Drinking Behavior" OR "Drinking Behaviour" OR "Alcohol Drinking" OR "Ethanol" OR "Ethanol" OR "alcohol" OR "Alcohol consumption" OR "Alcohol drinking" OR "alcohol use" OR "Energy Intake" OR "Weight Gain" OR "Weight gain" OR "gain weight" OR "gaining weight" OR "poor diet" OR "poor diets" OR "poor dietary" OR "healthy eating" OR "Smoking" OR "Smoking" OR "Smoking Cessation" OR "Smoking Reduction" OR "Tobacco Use Cessation" OR "Smoking cessation" OR "Tobacco" OR "Tobacco Products" OR "Tobacco use" OR "Tobacco use") OR DISKW("Exercise" OR "Physical activity" OR "Physical activities" OR "Exercise" OR "Exercises" OR "Exercising" OR "Exercis*" OR "Strength training" OR "Aerobic" OR "Aerobics" OR "Resistance training" OR "Walking" OR "Endurance Training" OR "Exergaming" OR "Gymnastics" OR "Interval Training" OR "Jogging" OR "Nordic Walking" OR "Physical Conditioning" OR "Running" OR "Stair Climbing" OR "Swimming" OR "Sitting" OR "Sedentary Behavior" OR "Sedentary behaviour" OR "Sedentary behavior" OR "Activity" OR "activities" OR "Inactivity" OR "inactivities" OR "Inactivit*" OR "Diet" OR "diet" OR "diets" OR "diet*" OR "nutrition" OR "nutritional" OR "nutrition*" OR "Eating" OR "eat" OR "eating" OR "consumption" OR "food intake" OR "food pattern" OR "food habit" OR "food patterns" OR "food habits" OR "food intake" OR "Food" OR "food" OR "foods" OR "food-related" OR "vegetable" OR "vegetables" OR "fruit" OR "fruits" OR "wholegrain" OR "wholegrains" OR "legume*" OR "nut" OR "nuts" OR "dairy" OR "fish" OR "tea" OR "fat" OR "fats" OR "oil" OR "oils" OR "coffee" OR "red meat" OR "processed meat" OR "Food and Beverages" OR "Beverages" OR "sweetened beverage*" OR "juice*" OR "Drinking Behavior" OR "Drinking Behavior" OR "Drinking Behaviour" OR "Alcohol Drinking" OR "Ethanol" OR "Ethanol" OR "alcohol" OR "Alcohol consumption" OR "Alcohol drinking" OR "alcohol use" OR "Energy Intake" OR "Weight Gain" OR "Weight gain" OR "gain weight" OR "gaining weight" OR "poor diet" OR "poor diets" OR "poor dietary" OR "healthy eating" OR "Smoking" OR "Smoking" OR "Smoking Cessation" OR "Smoking Reduction" OR "Tobacco Use Cessation" OR "Smoking cessation" OR "Tobacco" OR "Tobacco Products" OR "Tobacco use" OR "Tobacco use")) AND (TITLE("behavior change" OR "behavior changes" OR "behavior chang*" OR "behavioral change" OR "behavioral changes" OR "behavioral chang*" OR "behaviour change" OR "behaviour changes" OR "behaviour chang*" OR "behavioural change" OR "behavioural changes" OR "behavioural chang*" OR "behavior change interventions" OR "behavior change intervention" OR "behaviour change interventions" OR "behaviour change intervention" OR "behavioral change interventions" OR "behavioral change intervention" OR "behavioural change interventions" OR "behavioural change intervention" OR "behavior change techniques" OR "behavior change technique" OR "behaviour change techniques" OR "behaviour change technique" OR "behavioral change techniques" OR "behavioral change technique" OR "behavioural change techniques" OR "behavioural change technique" OR "Behavior Change" OR "Behavior Changes" OR "Behavior experiment" OR "Behavior experiments" OR "Behavior Intervention" OR "Behavior Interventions" OR "Behavior Modification" OR "Behavior Modifications" OR "Behavior Program" OR "Behavior Programme" OR "Behavior Programmes" OR "Behavior Programs" OR "Behavior Promotion" OR "Behavior Promotions" OR "Behavior Trial" OR "Behavior Trials" OR "Behavioral Change" OR "Behavioral Changes" OR "Behavioral experiment" OR "Behavioral experiments" OR "Behavioral Intervention" OR "Behavioral Interventions" OR "Behavioral Modification" OR "Behavioral Modifications" OR "Behavioral Program" OR "Behavioral Programme" OR "Behavioral Programmes" OR "Behavioral Programs" OR "Behavioral Promotion" OR "Behavioral Promotions" OR "Behavioral Trial" OR "Behavioral Trials" OR "Behaviors Change" OR "Behaviors Changes" OR "Behaviors experiment" OR "Behaviors experiments" OR "Behaviors Intervention" OR "Behaviors Interventions" OR "Behaviors Modification" OR "Behaviors Modifications" OR "Behaviors Program" OR "Behaviors Programme" OR "Behaviors Programmes" OR "Behaviors Programs" OR "Behaviors Promotion" OR "Behaviors Promotions" OR "Behaviors Trial" OR "Behaviors Trials" OR "Behaviour Change" OR "Behaviour Changes" OR "Behaviour experiment" OR "Behaviour experiments" OR "Behaviour Intervention" OR "Behaviour Interventions" OR "Behaviour Modification" OR "Behaviour Modifications" OR "Behaviour Program" OR "Behaviour Programme" OR "Behaviour Programmes" OR "Behaviour Programs" OR "Behaviour Promotion" OR "Behaviour Promotions" OR "Behaviour Trial" OR "Behaviour Trials" OR "Behavioural Change" OR "Behavioural Changes" OR "Behavioural experiment" OR "Behavioural experiments" OR "Behavioural Intervention" OR "Behavioural Interventions" OR "Behavioural Modification" OR "Behavioural Modifications" OR "Behavioural Program" OR "Behavioural Programme" OR "Behavioural Programmes" OR "Behavioural Programs" OR "Behavioural Promotion" OR "Behavioural Promotions" OR "Behavioural Trial" OR "Behavioural Trials" OR "Behaviours Change" OR "Behaviours Changes" OR "Behaviours experiment" OR "Behaviours experiments" OR "Behaviours Intervention" OR "Behaviours Interventions" OR "Behaviours Modification" OR "Behaviours Modifications" OR "Behaviours Program" OR "Behaviours Programme" OR "Behaviours Programmes" OR "Behaviours Programs" OR "Behaviours Promotion" OR "Behaviours Promotions" OR "Behaviours Trial" OR "Behaviours Trials") OR DISKW("behavior change" OR "behavior changes" OR "behavior chang*" OR "behavioral change" OR "behavioral changes" OR "behavioral chang*" OR "behaviour change" OR "behaviour changes" OR "behaviour chang*" OR "behavioural change" OR "behavioural changes" OR "behavioural chang*" OR "behavior change interventions" OR "behavior change intervention" OR "behaviour change interventions" OR "behaviour change intervention" OR "behavioral change interventions" OR "behavioral change intervention" OR "behavioural change interventions" OR "behavioural change intervention" OR "behavior change techniques" OR "behavior change technique" OR "behaviour change techniques" OR "behaviour change technique" OR "behavioral change techniques" OR "behavioral change technique" OR "behavioural change techniques" OR "behavioural change technique" OR "Behavior Change" OR "Behavior Changes" OR "Behavior experiment" OR "Behavior experiments" OR "Behavior Intervention" OR "Behavior Interventions" OR "Behavior Modification" OR "Behavior Modifications" OR "Behavior Program" OR "Behavior Programme" OR "Behavior Programmes" OR "Behavior Programs" OR "Behavior Promotion" OR "Behavior Promotions" OR "Behavior Trial" OR "Behavior Trials" OR "Behavioral Change" OR "Behavioral Changes" OR "Behavioral experiment" OR "Behavioral experiments" OR "Behavioral Intervention" OR "Behavioral Interventions" OR "Behavioral Modification" OR "Behavioral Modifications" OR "Behavioral Program" OR "Behavioral Programme" OR "Behavioral Programmes" OR "Behavioral Programs" OR "Behavioral Promotion" OR "Behavioral Promotions" OR "Behavioral Trial" OR "Behavioral Trials" OR "Behaviors Change" OR "Behaviors Changes" OR "Behaviors experiment" OR "Behaviors experiments" OR "Behaviors Intervention" OR "Behaviors Interventions" OR "Behaviors Modification" OR "Behaviors Modifications" OR "Behaviors Program" OR "Behaviors Programme" OR "Behaviors Programmes" OR "Behaviors Programs" OR "Behaviors Promotion" OR "Behaviors Promotions" OR "Behaviors Trial" OR "Behaviors Trials" OR "Behaviour Change" OR "Behaviour Changes" OR "Behaviour experiment" OR "Behaviour experiments" OR "Behaviour Intervention" OR "Behaviour Interventions" OR "Behaviour Modification" OR "Behaviour Modifications" OR "Behaviour Program" OR "Behaviour Programme" OR "Behaviour Programmes" OR "Behaviour Programs" OR "Behaviour Promotion" OR "Behaviour Promotions" OR "Behaviour Trial" OR "Behaviour Trials" OR "Behavioural Change" OR "Behavioural Changes" OR "Behavioural experiment" OR "Behavioural experiments" OR "Behavioural Intervention" OR "Behavioural Interventions" OR "Behavioural Modification" OR "Behavioural Modifications" OR "Behavioural Program" OR "Behavioural Programme" OR "Behavioural Programmes" OR "Behavioural Programs" OR "Behavioural Promotion" OR "Behavioural Promotions" OR "Behavioural Trial" OR "Behavioural Trials" OR "Behaviours Change" OR "Behaviours Changes" OR "Behaviours experiment" OR "Behaviours experiments" OR "Behaviours Intervention" OR "Behaviours Interventions" OR "Behaviours Modification" OR "Behaviours Modifications" OR "Behaviours Program" OR "Behaviours Programme" OR "Behaviours Programmes" OR "Behaviours Programs" OR "Behaviours Promotion" OR "Behaviours Promotions" OR "Behaviours Trial" OR "Behaviours Trials") OR ABSTRACT("behavior change" OR "behavior changes" OR "behavior chang*" OR "behavioral change" OR "behavioral changes" OR "behavioral chang*" OR "behaviour change" OR "behaviour changes" OR "behaviour chang*" OR "behavioural change" OR "behavioural changes" OR "behavioural chang*" OR "behavior change interventions" OR "behavior change intervention" OR "behaviour change interventions" OR "behaviour change intervention" OR "behavioral change interventions" OR "behavioral change intervention" OR "behavioural change interventions" OR "behavioural change intervention" OR "behavior change techniques" OR "behavior change technique" OR "behaviour change techniques" OR "behaviour change technique" OR "behavioral change techniques" OR "behavioral change technique" OR "behavioural change techniques" OR "behavioural change technique" OR "Behavior Change" OR "Behavior Changes" OR "Behavior experiment" OR "Behavior experiments" OR "Behavior Intervention" OR "Behavior Interventions" OR "Behavior Modification" OR "Behavior Modifications" OR "Behavior Program" OR "Behavior Programme" OR "Behavior Programmes" OR "Behavior Programs" OR "Behavior Promotion" OR "Behavior Promotions" OR "Behavior Trial" OR "Behavior Trials" OR "Behavioral Change" OR "Behavioral Changes" OR "Behavioral experiment" OR "Behavioral experiments" OR "Behavioral Intervention" OR "Behavioral Interventions" OR "Behavioral Modification" OR "Behavioral Modifications" OR "Behavioral Program" OR "Behavioral Programme" OR "Behavioral Programmes" OR "Behavioral Programs" OR "Behavioral Promotion" OR "Behavioral Promotions" OR "Behavioral Trial" OR "Behavioral Trials" OR "Behaviors Change" OR "Behaviors Changes" OR "Behaviors experiment" OR "Behaviors experiments" OR "Behaviors Intervention" OR "Behaviors Interventions" OR "Behaviors Modification" OR "Behaviors Modifications" OR "Behaviors Program" OR "Behaviors Programme" OR "Behaviors Programmes" OR "Behaviors Programs" OR "Behaviors Promotion" OR "Behaviors Promotions" OR "Behaviors Trial" OR "Behaviors Trials" OR "Behaviour Change" OR "Behaviour Changes" OR "Behaviour experiment" OR "Behaviour experiments" OR "Behaviour Intervention" OR "Behaviour Interventions" OR "Behaviour Modification" OR "Behaviour Modifications" OR "Behaviour Program" OR "Behaviour Programme" OR "Behaviour Programmes" OR "Behaviour Programs" OR "Behaviour Promotion" OR "Behaviour Promotions" OR "Behaviour Trial" OR "Behaviour Trials" OR "Behavioural Change" OR "Behavioural Changes" OR "Behavioural experiment" OR "Behavioural experiments" OR "Behavioural Intervention" OR "Behavioural Interventions" OR "Behavioural Modification" OR "Behavioural Modifications" OR "Behavioural Program" OR "Behavioural Programme" OR "Behavioural Programmes" OR "Behavioural Programs" OR "Behavioural Promotion" OR "Behavioural Promotions" OR "Behavioural Trial" OR "Behavioural Trials" OR "Behaviours Change" OR "Behaviours Changes" OR "Behaviours experiment" OR "Behaviours experiments" OR "Behaviours Intervention" OR "Behaviours Interventions" OR "Behaviours Modification" OR "Behaviours Modifications" OR "Behaviours Program" OR "Behaviours Programme" OR "Behaviours Programmes" OR "Behaviours Programs" OR "Behaviours Promotion" OR "Behaviours Promotions" OR "Behaviours Trial" OR "Behaviours Trials")) NOT TITLE("Telemedicine" OR "e-health" OR "m-health" OR "ehealth" OR "mhealth" OR "telehealth" OR "schoolbased" OR "school based" OR "digital intervention" OR "digital interventions" OR "digital" OR "online" OR "internet" OR "smartphone" OR "smartphones" OR "phone" OR "phones"))

**Google Scholar**

"behavior intervention"|"behavior intervention" "low social economic" "exercise"|"eating"|"smoking"|"alcohol"

"behaviour interventions"|"behaviour interventions" "low social economic" "exercise"|"eating"|"smoking"|"alcohol"

"behavior intervention"|"behavior intervention" "low socio economic" "exercise"|"eating"|"smoking"|"alcohol"

"behaviour interventions"|"behaviour interventions" "low socio economic" "exercise"|"eating"|"smoking"|"alcohol"

"behavior intervention"|"behavior intervention" "low socioeconomic" "exercise"|"eating"|"smoking"|"alcohol"

"behaviour interventions"|"behaviour interventions" "low socioeconomic" "exercise"|"eating"|"smoking"|"alcohol"

"behavior intervention"|"behavior intervention" "low SES" "exercise"|"eating"|"smoking"|"alcohol"

"behaviour interventions"|"behaviour interventions" "low SES" "exercise"|"eating"|"smoking"|"alcohol"

"behavior intervention"|"behavior intervention" "lower social economic" "exercise"|"eating"|"smoking"|"alcohol"

"behaviour interventions"|"behaviour interventions" "lower social economic" "exercise"|"eating"|"smoking"|"alcohol"

"behavior intervention"|"behavior intervention" "lower socio economic" "exercise"|"eating"|"smoking"|"alcohol"

"behaviour interventions"|"behaviour interventions" "lower socio economic" "exercise"|"eating"|"smoking"|"alcohol"

"behavior intervention"|"behavior intervention" "lower socioeconomic" "exercise"|"eating"|"smoking"|"alcohol"

"behaviour interventions"|"behaviour interventions" "lower socioeconomic" "exercise"|"eating"|"smoking"|"alcohol"

"behavior intervention"|"behavior intervention" "lower SES" "exercise"|"eating"|"smoking"|"alcohol"

"behaviour interventions"|"behaviour interventions" "lower SES" "exercise"|"eating"|"smoking"|"alcohol"

"gedrag" "interventie"|"interventies" "laag sociaal economisch" "bewegen"|"eten"|"dieet"|"roken"|"alcohol"|"drinken"

"gedrag" "interventie"|"interventies" "lager sociaal economisch" "bewegen"|"eten"|"dieet"|"roken"|"alcohol"|"drinken"

"gedragsinterventie"|"gedragsinterventies" "sociaal economisch" "bewegen"|"eten"|"dieet"|"roken"|"alcohol"|"drinken"

"gedrag" "interventie"|"interventies" "laag sociaaleconomisch" "bewegen"|"eten"|"dieet"|"roken"|"alcohol"|"drinken"

"gedrag" "interventie"|"interventies" "lager sociaaleconomisch" "bewegen"|"eten"|"dieet"|"roken"|"alcohol"|"drinken"

"gedragsinterventie"|"gedragsinterventies" "sociaaleconomisch" "bewegen"|"eten"|"dieet"|"roken"|"alcohol"|"drinken"

"gedrag" "interventie"|"interventies" "laag socio economisch" "bewegen"|"eten"|"dieet"|"roken"|"alcohol"|"drinken"

"gedrag" "interventie"|"interventies" "lager socio economisch" "bewegen"|"eten"|"dieet"|"roken"|"alcohol"|"drinken"

"gedragsinterventie"|"gedragsinterventies" "socio economisch" "bewegen"|"eten"|"dieet"|"roken"|"alcohol"|"drinken"

"gedrag" "interventie"|"interventies" "laag socioeconomisch" "bewegen"|"eten"|"dieet"|"roken"|"alcohol"|"drinken"

"gedrag" "interventie"|"interventies" "lager socioeconomisch" "bewegen"|"eten"|"dieet"|"roken"|"alcohol"|"drinken"

"gedragsinterventie"|"gedragsinterventies" "socioeconomisch" "bewegen"|"eten"|"dieet"|"roken"|"alcohol"|"drinken"

"gedrag" "interventie"|"interventies" "laag SES" "bewegen"|"eten"|"dieet"|"roken"|"alcohol"|"drinken"

"gedrag" "interventie"|"interventies" "lager SES" "bewegen"|"eten"|"dieet"|"roken"|"alcohol"|"drinken"

"gedragsinterventie"|"gedragsinterventies" "SES" "bewegen"|"eten"|"dieet"|"roken"|"alcohol"|"drinken"
